# Screening traditional Chinese medicine monomers for premature ovarian insufficiency: from network meta-analysis to the effects of a promising candidate on oxidative stress biomarkers

**DOI:** 10.3389/fphar.2026.1934959

**Published:** 2026-09-18

**Authors:** Yanping Tan, Mei Zhao, Lianwei Xu

**Affiliations:** Department of Gynecology, Longhua Hospital, Shanghai University of Traditional Chinese Medicine, Shanghai, China

**Keywords:** network meta-analysis, oxidative stress, premature ovarian insufficiency, quercetin, traditional Chinese medicine monomers

## Abstract

**Background:**

Therapeutic options for premature ovarian insufficiency (POI) are limited, and the comparative efficacy of traditional Chinese medicine (TCM) monomers remains unclear. This study aimed to systematically evaluate and rank the efficacy of various TCM monomers in animal models of POI and to explored the potential role of oxidative stress modulation by a promising candidate.

**Methods:**

Eight electronic databases (PubMed, Embase, the Cochrane Library, Web of Science, CNKI, VIP, Wanfang Database, and SinoMed) were searched from inception to 3 April 2026, for randomized controlled animal trials evaluating single TCM monomers in POI models. The SYRCLE tool and CINeMA were employed to assess the risk of bias and the certainty of evidence, respectively. A frequentist network meta-analysis was conducted to compare primary and secondary ovarian function outcomes, with relative efficacy ranked by the surface under the cumulative ranking curve (SUCRA) values. Pairwise meta-analyses were then performed to evaluate the effects of a candidate monomer on oxidative stress biomarkers. Three-dimensional scatter plots were used to visualize potential trends in primary outcomes regarding the relationships among dose, treatment duration, and therapeutic efficacy.

**Results:**

Thirty-two studies involving 676 animals and six TCM monomers were included. Compared with controls, all monomers significantly reduced serum follicle-stimulating hormone and increased estradiol levels. Furthermore, quercetin and icariin promoted follicular development and suppressed atresia., although the certainty of evidence was very low. Based on global mean SUCRA values, quercetin ranked first in the main analysis and emerged as a promising candidate overall. Pairwise analyses showed that quercetin increased serum superoxide dismutase and catalase while decreasing malondialdehyde levels. Exploratory three-dimensional visualization suggested a potential enhanced therapeutic trend for quercetin within the dosage range of 25–100 mg/kg over 10–30 days.

**Conclusion:**

Several TCM monomers improved ovarian function in POI models. Quercetin was considered a promising candidate, with its potential effect likely associated with the modulation of oxidative stress biomarkers. Given the substantial heterogeneity and very low evidence certainty, these findings should be interpreted with caution. Further rigorous, standardized preclinical dose-response studies and well-designed clinical trials are warranted to facilitate the clinical translation of these findings.

**Systematic Review Registration:**

https://www.crd.york.ac.uk/PROSPERO/view/CRD420261359490, identifier CRD420261359490.

## 1 Introduction

Premature ovarian insufficiency (POI), previously termed premature ovarian failure (POF), is characterized by oligomenorrhea or amenorrhea, elevated follicle-stimulating hormone (FSH) and depleted estradiol (E_2_) before age 40 (The ESHRE Guideline Group on POI et al., 2016). It affects 1%–3.7% of women and arises from multifactorial etiologies, including genetic variants, autoimmune disorders, and iatrogenic injury (Guo et al., 2025). Untreated POI causes psychological distress and increases the long-term risks of cardiovascular disease, osteoporosis, and dementia (Nash and Davies, 2024). Current clinical management relies mainly on hormone replacement therapy (HRT) and assisted reproductive technology (ART). However, HRT is limited by contraindications and adverse effects, whereas ART fails to restore ovarian function (Touraine et al., 2024). Thus, exploring safer and more effective therapeutic strategies is of great significance, which necessitates a deeper understanding of the molecular mechanisms underlying ovarian decline.

Although the pathogenesis of POI remains incompletely understood, apoptosis, inflammation, and oxidative stress are considered important contributors to disease progression and ovarian aging (Camp et al., 2025; Sharata et al., 2025). Oxidative stress refers to a state in which the production of reactive oxygen species (ROS) exceeds the capacity of endogenous antioxidant defenses, resulting in a disruption of cellular redox homeostasis (Shi et al., 2023). Maintaining this balance is essential for ovarian function, as physiological ROS levels participate in follicular growth, ovulation, and corpus luteum regression (Sugino, 2005). In contrast, ROS overaccumulation can cause cellular injury, impair oocyte development, and induce granulosa cell (GC) apoptosis (Wang et al., 2021). Specifically, this oxidative insult inflicts DNA damage and mitochondrial dysfunction, activates apoptotic signaling, and may impair ovarian angiogenesis (Shi et al., 2023). Given that GCs provide metabolic and survival support to oocytes, their dysfunction or apoptosis triggers follicle atresia and ultimately depletes the ovarian reserve (Liu et al., 2023). Therefore, evaluating oxidative stress biomarkers, such as antioxidant enzymes activities and lipid peroxidation products, provides critical insights into ovarian injury and therapeutic efficacy.

Traditional Chinese medicine (TCM) monomers have attracted increasing interest for their ovarian-protective effects. Recent preclinical studies suggest that these compounds improve serum sex hormone levels and ovarian morphology through multiple pathways (Feng et al., 2025; Li M et al., 2025), among which antioxidant activity plays a pivotal role (Shi et al., 2023). However, evidence remains largely confined to animal studies, where substantial heterogeneity in modeling methods, dosages, and treatment durations complicates direct comparison of relative efficacy. Furthermore, current clinical data are severely limited, precluding a high-quality clinical network meta-analysis of TCM monomers for POI. Consequently, a rigorous synthesis of preclinical evidence is warranted to compare TCM monomers and inform future clinical research.

Although a previous meta-analysis of POF animal models provided valuable evidence, it pooled the effects of diverse natural products and did not compare the relative efficacy of individual compounds (Hu et al., 2024). To address this gap, we treated each eligible TCM monomer as a separate intervention and conducted a frequentist network meta-analysis to evaluate and rank their efficacy in animal models of POI. We also conducted subgroup and sensitivity analyses to explore potential sources of heterogeneity and assess the robustness of the findings. Given the pivotal role of oxidative stress in POI and the antioxidant properties of TCM monomers, we subsequently performed a pairwise meta-analysis on oxidative stress biomarkers for a candidate monomer, aiming to provide a reference for the rational design of future preclinical and clinical therapeutic strategies.

## 2 Methods

### 2.1 Search strategy and study selection

The study adhered to the PRISMA guidelines and was registered in PROSPERO (CRD420261359490; available from https://www.crd.york.ac.uk/PROSPERO/view/CRD420261359490). We systematically searched PubMed, Embase, the Cochrane Library, Web of Science, Chinese National Knowledge Infrastructure Database (CNKI), Chinese Science and Technique Journals Database (VIP), Wanfang Database, and SinoMed from their inception to 3 April 2026, without language restrictions. Detailed search strategies are provided in Supplementary Table S1.

We included randomized controlled animal studies evaluating single TCM monomers in female mammalian models explicitly described as POI/POF and validated by estrous cycle, serum hormone levels, or follicle counts. Control groups received vehicle, normal saline, distilled water, or no treatment. To ensure reliable effect estimation and meaningful comparison, monomers evaluated in fewer than three independent publications or those involving local administration were excluded. Eligible studies were required to report at least one ovarian function outcome. Primary outcomes included serum FSH, anti-Müllerian hormone (AMH), and antral follicle count (AFC); secondary outcomes included E_2_, luteinizing hormone (LH), as well as the number of atretic follicles and early-stage follicles (primordial, primary, and secondary follicles).

The main network meta-analysis aimed to rank TCM monomers according to their most favorable observed experimental effects (Lin et al., 2024; Luo et al., 2025). When multiple dose arms for the same monomer were reported in a study, the best-performing dose arm was selected based on the primary ovarian function outcomes. To characterize the oxidative stress profile of the identified candidate monomer, we subsequently conducted a pairwise meta-analysis on oxidative stress biomarkers across all eligible dose arms. The analyzed biomarkers included superoxide dismutase (SOD), catalase (CAT), and glutathione peroxidase (GPx) as representative antioxidant enzymes, and malondialdehyde (MDA) as a marker of lipid peroxidation (Chen et al., 2024).

Two reviewers independently screened literature titles and abstracts, followed by a full-text review for final eligibility. Any discrepancies were resolved through consensus or consultation with a third senior reviewer.

### 2.2 Data extraction

Two reviewers independently extracted and cross-checked data from eligible articles, including the first author, publication year, animal characteristics (species, weight, age, and sample sizes), induction method, intervention details (drug, dosage, approach and duration) and final outcomes. Missing or purely graphical data were requested from corresponding authors; when unavailable, values were digitized from figures using WebPlotDigitizer (version 4.8).

### 2.3 Quality assessment

Two reviewers independently assessed risk of bias using the SYRCLE’s RoB tool, which comprises 10 items across six domains (Hooijmans et al., 2014). Each item was categorized as low, high, or unclear risk. Disagreements were resolved by discussion. The certainty of evidence for network meta-analysis estimates was evaluated using CINeMA, which appraises six domains: within-study bias, reporting bias, indirectness, imprecision, heterogeneity, and incoherence (Nikolakopoulou et al., 2020).

### 2.4 Statistical analysis

Frequentist network meta-analyses were performed using the netmeta package in R (version 4.4.0). Continuous outcomes were pooled as standardized mean differences (SMDs) with 95% confidence intervals (CIs) to account for diverse scales across studies. Heterogeneity was quantified using the *I* ^2^ statistic and Cochran’s *Q* test, and the between-study variance (τ^2^) was estimated using the restricted maximum likelihood (REML) estimator under a random-effects model. To estimate the dispersion of true effects in future settings, 95% prediction intervals (PIs) were also calculated (Archer et al., 2018). Global and local inconsistency assessments were inapplicable because the only closed loop was formed by a single multi-arm trial. Forest plots and league tables presented treatment effects, while surface under the cumulative ranking curve (SUCRA) values and cumulative probability plots ranked the relative efficacy of the monomers. Subgroup analyses of the primary outcomes were conducted according to animal species, induction methods, and treatment duration. Sensitivity analyses were performed by excluding small-sample studies (*n* < 5 animals per group) to assess the robustness of the results. Publication bias was evaluated using comparison-adjusted funnel plots and Egger’s test when at least ten studies were available.

Radar charts were used to visualize SUCRA values of different interventions across multiple outcomes. For multidimensional evaluation, the overall efficacy of interventions was ranked using the global mean SUCRA, calculated as the sum of SUCRA values across all outcomes divided by the total number of predefined endpoints. Missing SUCRA values for individual endpoints were assigned a value of zero to penalize incomplete outcome reporting (Daly et al., 2020; Riley et al., 2017). The monomer with the highest overall ranking in the main all-dose network meta-analysis was selected as a candidate monomer. To evaluate the impact of assigning zero to missing SUCRA values on treatment rankings, sensitivity analyses were performed using available-case data. Additionally, we conducted an all-dose network meta-analysis including all eligible dose arms, restricted to SUCRA rankings to assess the sensitivity of monomer ranking. Pairwise meta-analyses were then performed with the meta package to assess the effects of the candidate monomer on oxidative stress biomarkers for improving ovarian function. Shared control groups were split equally following the Cochrane Handbook to prevent double-counting. Finally, three-dimensional scatter plots were used to visualize potential trends in primary outcomes regarding the relationships among dose, treatment duration, and therapeutic efficacy.

## 3 Results

### 3.1 Study characteristics

Of the 2,246 records retrieved, 115 full-text articles were reviewed, yielding 32 eligible studies (Ai, 2007; Chen, 2022; Chen et al., 2025; Ding and Xu, 2025; Eren et al., 2024; Fang et al., 2025; Guo et al., 2024; Haddad et al., 2020; Huang et al., 2020; Ji et al., 2025; Jiang et al., 2025; Li et al., 2024; Li, 2013; Li, 2018; Li et al., 2019; Li M et al., 2025; Mai et al., 2025; Mobasher et al., 2022; Moradi et al., 2023; Özatik et al., 2016; Said et al., 2016; Tong, 2019; Wang et al., 2025; Xing et al., 2023; Xue and Zhao, 2023; Yang et al., 2026; Yang et al., 2024; Yu et al., 2026; Zhang et al., 2021; Zheng et al., 2021; Zhou, 2024; Zhu et al., 2023) for the final analysis (Figure 1). The eligible studies involved 676 animals, comprising 332 in the model group and 344 in the treatment group. Table 1 outlines six investigated monomers: quercetin (10 studies) (Eren et al., 2024; Guo et al., 2024; Huang et al., 2020; Ji et al., 2025; Jiang et al., 2025; Li, 2013; Mai et al., 2025; Moradi et al., 2023; Wang et al., 2025; Zheng et al., 2021), icariin (Chen, 2022; Li et al., 2024; Li et al., 2019; Tong, 2019; Xue and Zhao, 2023; Yang et al., 2026; Zhu et al., 2023) and resveratrol (Chen et al., 2025; Li, 2018; Mobasher et al., 2022; Özatik et al., 2016; Said et al., 2016; Yu et al., 2026; Zhou, 2024) (7 studies each), as well as astragaloside IV (Li M et al., 2025; Xing et al., 2023; Yang et al., 2024), genistein (Ai, 2007; Haddad et al., 2020; Li, 2013), and puerarin (Ding and Xu, 2025; Fang et al., 2025; Zhang et al., 2021) (3 studies each). Methodological quality assessment indicated that the risk of bias across all included studies ranged from low to unclear (Figure 2; Supplementary Table S2). The certainty of evidence was rated as low to very low, primarily due to risk of bias, substantial heterogeneity, indirectness, and incoherence (Supplementary Table S3). Furthermore, potential publication bias was observed for most outcomes (Supplementary Figures S1-S2), and detailed heterogeneity assessments are summarized in Supplementary Tables S4-S5.

**FIGURE 1:**
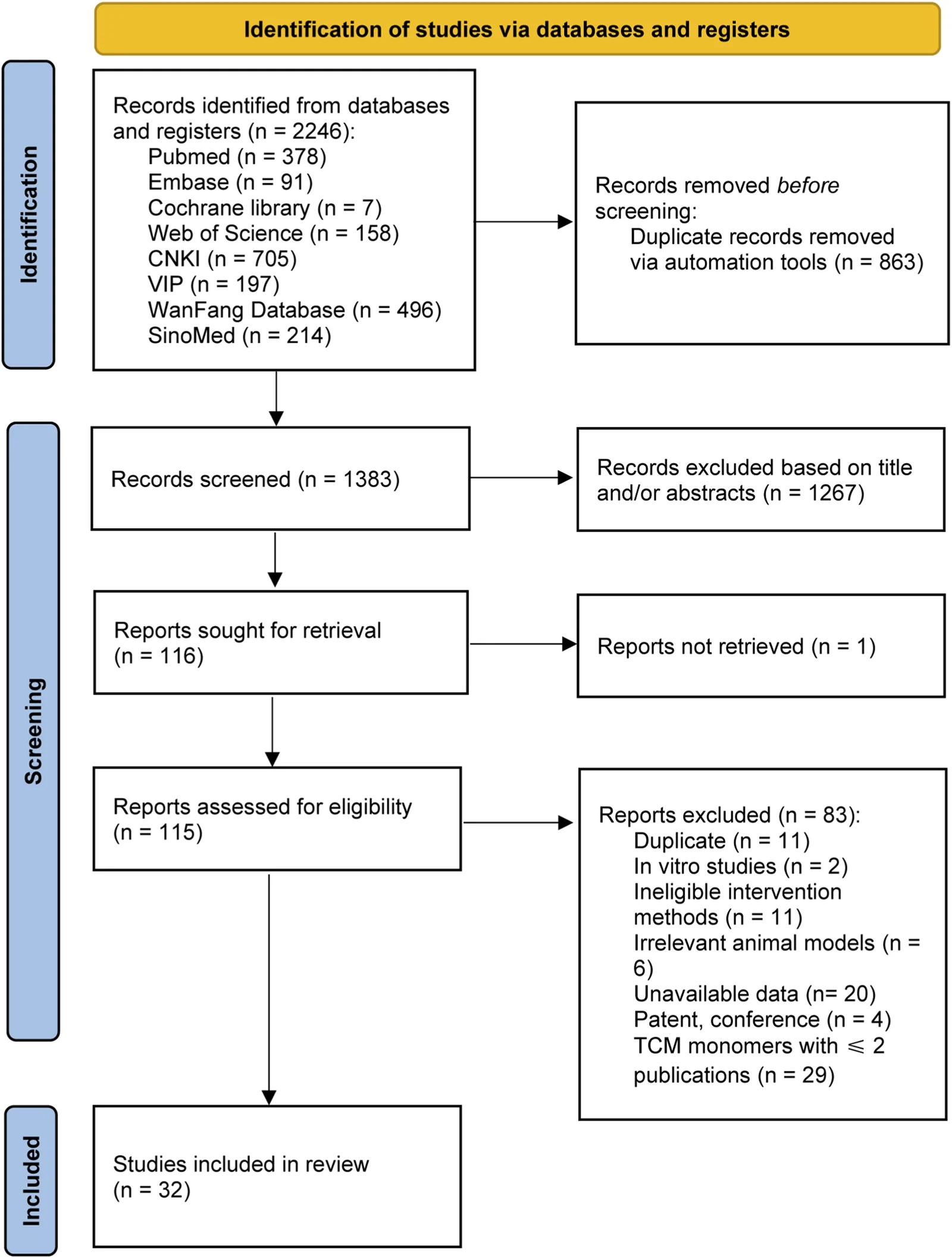
Flowchart of study selection.

**TABLE 1:** Basic characteristics of the included studies.

Study ID (first author, year)	Species (weight, age)	Modeling induction drug (dosage, approach, frequency, duration)	Sample sizes (T/M)	Control group	Treatment drug (dosage, approach, frequency, duration)	Outcomes
Li, 2018	Sprague-Dawley rats (200 ± 20 g, 5 weeks)	Tripterygium glycosides (50 mg/kg, i.g., qd, 14 days)	18/18	0.5% CMC-Na solution	Resveratrol (80 mg/kg, i.g., qd, 42 days)	①②④⑤⑨
Chen et al., 2025	ICR mice (30 ± 0.5 g, 8–9 weeks)	D-galactose (200 mg/kg, s.c., qd, 42 days)	15/15	NS	Resveratrol (30 mg/kg, i.g., qd, 42 days)	①②④⑤⑦⑨
Zhou, 2024	C57BL/6 mice (18–21g, 8–9 weeks)	D-galactose (450 mg/kg, i.p., qd, 63 days)	10/10	NS	Resveratrol (100 mg/kg, i.g., qd, 28 days)	①④⑤
Zhang et al., 2021	C57BL/6 mice (19–24 g, 6 weeks)	Cisplatin (4.5 mg/kg, i.p., qd, 5 days)	10/10	NS	Puerarin (200 mg/kg, i.p., qd, 14 days)	①②④
Ding and Xu, 2025	Sprague-Dawley rats (180–200 g, NR)	Cyclophosphamide (D1: 200 mg/kg, D2–D15: 8 mg/kg; i.p., qd, 15 days)	6/6	NS	Puerarin (50 mg/kg, i.p., qd, 21 days)	①②④⑤⑨
Jiang et al., 2025	Sprague-Dawley rats (170 ± 10 g, 8 weeks)	Cyclophosphamide (D1: 50 mg/kg, D2–D7: 5 mg/kg; i.p., qd, 7 days)	10/10	NS	Quercetin (100 mg/kg, i.g., qd, 30 days)	①②③④⑦⑧⑨
Guo et al., 2024	Sprague-Dawley rats (210 ± 20 g, NR)	Cyclophosphamide (D1: 50 mg/kg, D2–D15: 8 mg/kg; i.p., qd, 15 days)	12/12	NS	Quercetin (100 mg/kg, i.g., qd, 14 days)	①②④⑨
Li, 2013	BALB/c mice (21–23 g, 7–9 weeks)	Peptide zona pellucida 3 (D0: 0.15mL, CFA-pZP3, D14: 0.15mL, IFA-pZP3; s.c., D0, D14, 14 days)	12/12	DMSO	Quercetin (45 mg/kg, i.g., qd, 50 days)	①④⑤
Li, 2013	BALB/c mice (21–23 g, 7–9 weeks)	Peptide zona pellucida 3 (D0: 0.15mL, CFA-pZP3, D14: 0.15mL, IFA-pZP3; s.c., D0, D14, 14 days)	12/12	DMSO	Genistein (45 mg/kg, i.g., qd, 50 days)	①④⑤
Mai et al., 2025	C57BL/6 mice (20 ± 5 g, 5 weeks)	Cyclophosphamide (90 mg/kg, i.p., once, 1 day)	10/10	NS	Quercetin (50 mg/kg, i.g., qd, 21 days)	①②④
Yang et al., 2024	Sprague-Dawley rats (180–220 g, NR)	Cyclophosphamide (50 mg/kg, i.p., qd, 15 days)	10/10	NR	Astragaloside IV (40 mg/kg, i.g., qd, 24 days)	①②④
Xing et al., 2023	Sprague-Dawley rats (200.35 ± 20.87 g, NR)	Cyclophosphamide (D1: 50 mg/kg, D2–D15: 8 mg/kg; i.p., qd, 15 days)	12/12	1% CMC-Na solution	Astragaloside IV (40 mg/kg, i.g., qd, 28 days)	①②③④⑤⑦⑨
Chen, 2022	BALB/c mice (18 ± 2 g, 7 weeks)	Peptide zona pellucida 3 (7.5 mg/kg, s.c., D0, D14, 14 days)	21/21	PBS	Icariin (40 mg/kg, i.g., qd, 28 days)	①②⑤⑥⑦⑧⑨
Li Y et al., 2025	Sprague-Dawley rats (205–230 g, 7–8 weeks)	Cyclophosphamide (D1: 50 mg/kg, D2–D15: 5 mg/kg; i.p., qd, 15 days)	10/10	NS	Astragaloside IV (80 mg/kg, i.g., qd, 28 days)	①②③④⑤⑥⑦
Ai, 2007	ICR mice (28–30 g, NR)	Cisplatin (3.0 mg/kg, i.p., qd, 7 days)	20/20	NR	Genistein (100 mg/kg, i.g., qd, 14 days)	④
Huang et al., 2020	Sprague-Dawley rats (220–250 g, NR)	Tripterygium glycosides (75 mg/kg, i.g., qd, 30 days)	10/10	NS	Quercetin (600 mg/kg, i.g., qd, 30 days)	①②④⑤
Zhu et al., 2023	Sprague-Dawley rats (240 ± 20 g, 10 weeks)	Cyclophosphamide (D1: 200 mg/kg, D2–D16: 8 mg/kg; i.p., qd, 16 days)	10/10	NS	Icariin (60 mg/kg, i.g., qd, 28 days)	①②④⑨
Xue and Zhao, 2023	C57BL/6 mice (18 ± 1 g, 6 weeks)	Cyclophosphamide (40 mg/kg, i.p., qd, 14 days)	10/10	NS	Icariin (25 mg/kg, i.g., qd, 28 days)	①④⑥⑦⑧
Tong, 2019	Wistar rats (NR, 10 weeks)	Cisplatin (4 mg/kg, i.p., D14, D21, 7 days)	10/10	NR	Icariin (25 mg/kg, i.p., q3d, 28 days)	②⑥
Mobasher et al., 2022	Wistar albino rats (200–240 g, 12–16 weeks)	Cyclophosphamide (D1: 200 mg/kg, D2–D14: 8 mg/kg; i.p., qd, 14 days)	7/7	NR	Resveratrol (10 mg/kg, i.g., qd, 21 days)	①④⑤
Eren et al., 2024	Wistar rats (200 ± 40 g, 10–12 weeks)	Cyclophosphamide (100 mg/kg, i.p., qd, 7 days)	12/12	NS	Quercetin (100 mg/kg, i.p., q7d, 15 days)	②
Zheng et al., 2021	Sprague-Dawley rats (260 ± 20 g, 12 weeks)	Cyclophosphamide (100 mg/kg, i.p., once, 1 day)	10/10	Distilled water	Quercetin (100 mg/kg, i.g., qd, 30 days)	①②③④⑤⑥⑦⑧⑨
Özatik et al., 2016	Sprague-Dawley rats (40–50 g, 28 days)	4-Vinylcyclohexene diepoxide (60 mg/kg, i.p., qd, 15 days)	6/6	NR	Resveratrol (10 mg/kg, i.g., qd, 3 days)	④
Li et al., 2024	ICR mice (NR, 6–8 weeks)	Cisplatin (5 mg/kg, i.p., q5d, 21 days)	6/6	NR	Icariin (30 mg/kg, i.p., qd, 21 days)	③⑥⑦⑧
Yang et al., 2026	C57BL/6 mice (NR, 6–7 weeks)	Cisplatin (2 mg/kg, i.p., qd, 7 days)	6/6	Distilled water	Icariin (160 mg/kg, i.g., qd, 21 days)	①②④⑤
Li et al., 2019	C57BL/6 mice (NR, NR)	D-galactose (200 mg/kg, s.c., qd, 42 days)	10/10	NR	Icariin (100 mg/kg, i.p., qd, 42 days)	①②③④⑤⑥⑦⑧⑨
Wang et al., 2025	Sprague-Dawley rats (200 g, 8 weeks)	Cyclophosphamide (D1: 200 mg/kg, D2–D15: 8 mg/kg; i.p., qd, 15 days)	6/6	NR	Quercetin (100 mg/kg, i.g., qd, 30 days)	①②④⑤
Yu et al., 2026	Sprague-Dawley rats (180–200 g, NR)	Cyclophosphamide (D1: 60 mg/kg, D2–D14: 8 mg/kg; i.p., qd, 14 days)	6/6	NR	Resveratrol (10 mg/kg, i.g., qd, 14 days)	①②④
Haddad et al., 2020	Sprague-Dawley rats (30–40 g, 3 weeks)	Radiation (3.2 Gy, whole-body gamma radiation, once, 1 day)	20/20	DMSO and corn oil at a ratio of 1:10	Genistein (5 mg/kg, i.p., qd, 10 days)	②③④⑥⑨
Fang et al., 2025	Sprague-Dawley rats (180–200 g, 6 weeks)	Cyclophosphamide (D1: 200 mg/kg, D2–D15: 8 mg/kg; i.p., qd, 15 days)	6/6	NS	Puerarin (50 mg/kg, i.p., qd, 21 days)	①②④⑤⑨
Said et al., 2016	Sprague-Dawley rats (30–40 g, 23 days)	Radiation (3.2 Gy, whole-body gamma radiation, once, 1 day)	10/10	DMSO and cornoil at a ratio of 5:95	Resveratrol (25 mg/kg, i.g., qd, 17 days)	②③⑥⑨
Ji et al., 2025	BALB/c mice (20–22 g, 5 weeks)	Imatinib (120 mg/kg, i.g., qd, 42 days)	5/5	NR	Quercetin (100 mg/kg, i.g., qd, 42 days)	②④⑤⑥⑦⑧⑨
Moradi et al., 2023	Wistar albino rats (210 ± 10 g, 12–16 weeks)	Cyclophosphamide (D1: 200 mg/kg, D2–D4: 8 mg/kg; i.p., qd, 4 days)	6/6	NR	Quercetin (100 mg/kg, i.p., qd, 14 days)	②③④⑦

M: model group; T: treatment group; ①: follicle-stimulating hormone (FSH); ②: anti-Müllerian hormone (AMH); ③: antral follicle count (AFC); ④: estradiol (E_2_); ⑤: luteinizing hormone (LH); ⑥: primordial follicle; ⑦: primary follicle; ⑧: secondary follicle; ⑨: atretic follicle; CMC-Na: sodium carboxymethyl cellulose; DMSO: dimethyl sulfoxide; PBS: phosphate buffered saline; NS: normal saline; i. g.: intragastric administration; i. p.: intraperitoneal injection; s. c.: subcutaneous injection; qd: once daily; q3d: once every 3 days; q7d: once every 7 days; NR: not reported.

**FIGURE 2:**
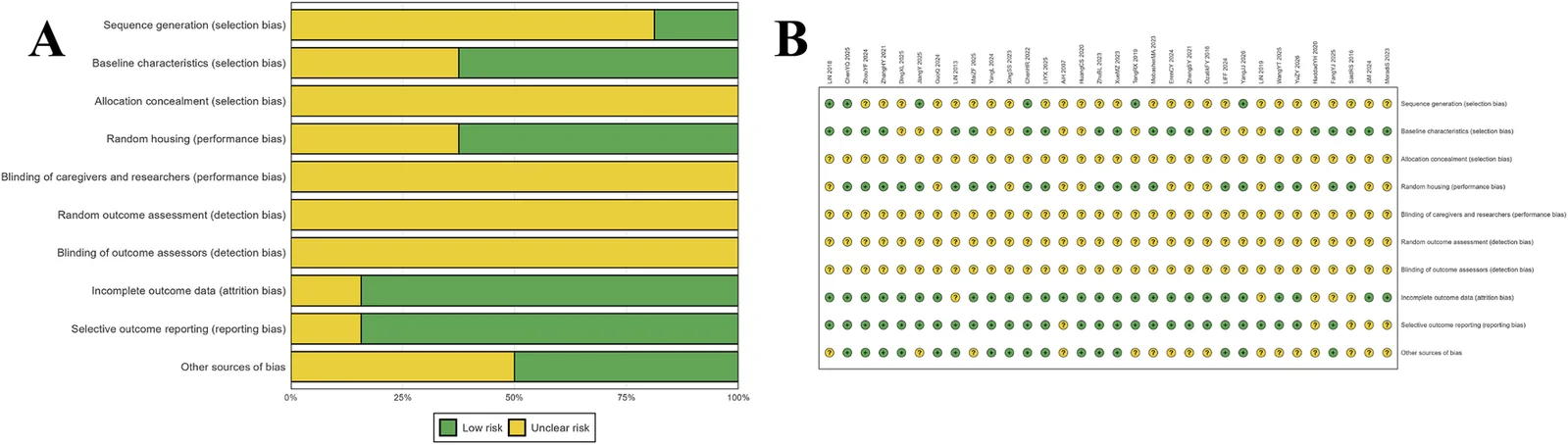
Risk of bias of the included studies. **(A)** Risk of bias graph. **(B)** Risk of bias summary.

### 3.2 Primary outcomes

Network plots for primary outcomes illustrated 23 studies on FSH (Chen, 2022; Chen et al., 2025; Ding and Xu, 2025; Fang et al., 2025; Guo et al., 2024; Huang et al., 2020; Jiang et al., 2025; Li, 2013; Li, 2018; Li et al., 2019; Li M et al., 2025; Mai et al., 2025; Mobasher et al., 2022; Wang et al., 2025; Xing et al., 2023; Xue and Zhao, 2023; Yang et al., 2026; Yang et al., 2024; Yu et al., 2026; Zhang et al., 2021; Zheng et al., 2021; Zhou, 2024; Zhu et al., 2023) (Figure 3A), 25 on AMH (Chen, 2022; Chen et al., 2025; Ding and Xu, 2025; Eren et al., 2024; Fang et al., 2025; Guo et al., 2024; Haddad et al., 2020; Huang et al., 2020; Ji et al., 2025; Jiang et al., 2025; Li, 2018; Li et al., 2019; Li M et al., 2025; Mai et al., 2025; Moradi et al., 2023; Said et al., 2016; Tong, 2019; Wang et al., 2025; Xing et al., 2023; Yang et al., 2026; Yang et al., 2024; Yu et al., 2026; Zhang et al., 2021; Zheng et al., 2021; Zhu et al., 2023) (Supplementary Figure S3A), and 9 on AFC (Haddad et al., 2020; Jiang et al., 2025; Li et al., 2024; Li et al., 2019; Li M et al., 2025; Moradi et al., 2023; Said et al., 2016; Xing et al., 2023; Zheng et al., 2021) (Supplementary Figures S4A). League tables and forest plots showed that all six monomers significantly reduced serum FSH compared with controls (Figures 3B,D). For increasing AMH levels, icariin (SMD = 10.18, 95% CI: 7.22 to 13.13, 95% PI: 3.47 to 16.88; heterogeneity: *I* ^2^ = 92.80%, *P* < 0.001), puerarin (SMD = 6.93, 95% CI: 3.29 to 10.57, 95% PI: −0.15 to 14.01; heterogeneity: *I* ^2^ = 91.12%, *P* < 0.001), quercetin (SMD = 4.96, 95% CI: 2.96 to 6.95, 95% PI: −1.33 to 11.24; heterogeneity: *I* ^2^ = 92.92%, *P* < 0.001), and astragaloside IV (SMD = 4.08, 95% CI: 0.76 to 7.40, 95% PI: −2.82 to 10.98; heterogeneity: *I* ^2^ = 61.84%, *P* = 0.073) were effective (Supplementary Figures S1-S3B, D). Quercetin (SMD = 5.33, 95% CI: 1.78 to 8.88, 95% PI: −4.01 to 14.67; heterogeneity: *I* ^2^ = 95.14%, *P* < 0.001) and astragaloside IV (SMD = 5.06, 95% CI: 0.94 to 9.18, 95% PI: −4.74 to 14.85; heterogeneity: *I* ^2^ = 66.80%, *P* = 0.083) also substantially increased AFC (Supplementary Figures S4B, D). Although the pooled effect was statistically significant, the high heterogeneity and 95% PIs encompassing zero suggest that the true intervention effects may vary substantially across different experimental settings. Cumulative ranking probabilities identified puerarin, icariin, and quercetin as the potential candidate monomers for reducing FSH, increasing AMH and elevating AFC, respectively (Figure 3C; Supplementary Figure S3C; Supplementary Figure S4C).

**FIGURE 3:**
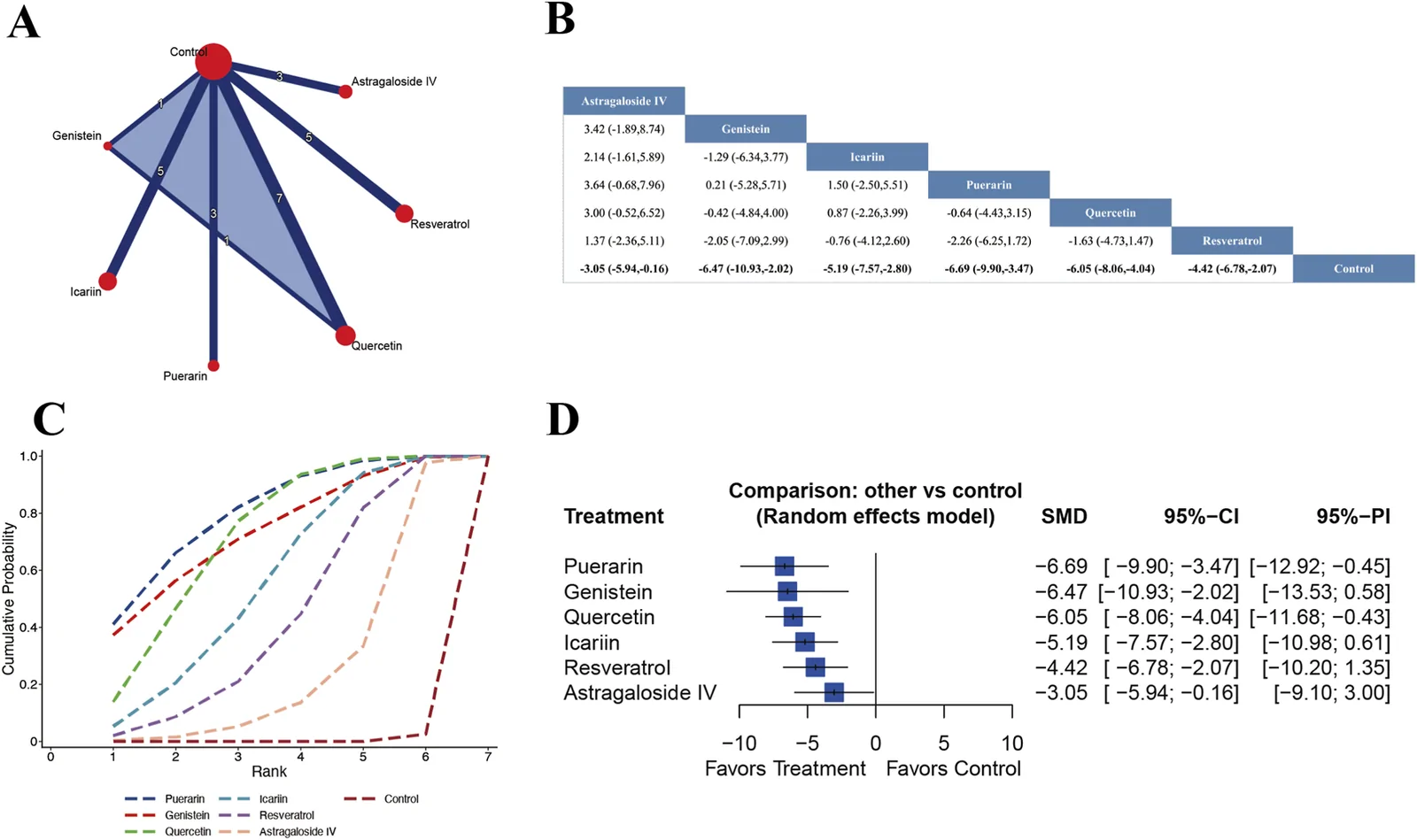
Network meta-analysis of FSH levels. **(A)** Network plot of eligible comparisons, where node sizes are proportional to the total animal sample sizes, and line widths correspond to the number of direct comparisons. The numbers on the lines represent the number of trials. The shaded area denotes a multi-arm trial. **(B)** League table summarizing all pairwise comparisons. Data are expressed as standardized mean differences (SMDs) with 95% confidence intervals (CIs). Bold values indicate statistical significance. **(C)** SUCRA plots showing cumulative ranking probabilities. A larger area under the curve indicates a higher probability of the intervention being top-ranked. **(D)** Forest plot comparing the effects of TCM monomers with the control group based on a random-effects model.

### 3.3 Secondary outcomes

Network analyses for secondary outcomes illustrated 27 studies on E_2_ (Ai, 2007; Chen et al., 2025; Ding and Xu, 2025; Fang et al., 2025; Guo et al., 2024; Haddad et al., 2020; Huang et al., 2020; Ji et al., 2025; Jiang et al., 2025; Li, 2013; Li, 2018; Li et al., 2019; Li M et al., 2025; Mai et al., 2025; Mobasher et al., 2022; Moradi et al., 2023; Özatik et al., 2016; Wang et al., 2025; Xing et al., 2023; Xue and Zhao, 2023; Yang et al., 2026; Yang et al., 2024; Yu et al., 2026; Zhang et al., 2021; Zheng et al., 2021; Zhou, 2024; Zhu et al., 2023), 16 on LH (Chen, 2022; Chen et al., 2025; Ding and Xu, 2025; Fang et al., 2025; Huang et al., 2020; Ji et al., 2025; Li, 2013; Li, 2018; Li et al., 2019; Li M et al., 2025; Mobasher et al., 2022; Wang et al., 2025; Xing et al., 2023; Yang et al., 2026; Zheng et al., 2021; Zhou, 2024), 10 on primordial follicles (Chen, 2022; Haddad et al., 2020; Ji et al., 2025; Li et al., 2024; Li et al., 2019; Li M et al., 2025; Said et al., 2016; Tong, 2019; Xue and Zhao, 2023; Zheng et al., 2021), 11 on primary follicles (Chen, 2022; Chen et al., 2025; Ji et al., 2025; Jiang et al., 2025; Li et al., 2024; Li et al., 2019; Li M et al., 2025; Moradi et al., 2023; Xing et al., 2023; Xue and Zhao, 2023; Zheng et al., 2021), 7 on secondary follicles (Chen, 2022; Ji et al., 2025; Jiang et al., 2025; Li et al., 2024; Li et al., 2019; Xue and Zhao, 2023; Zheng et al., 2021), and 14 on atretic follicles (Chen, 2022; Chen et al., 2025; Ding and Xu, 2025; Fang et al., 2025; Guo et al., 2024; Haddad et al., 2020; Ji et al., 2025; Jiang et al., 2025; Li, 2018; Li et al., 2019; Said et al., 2016; Xing et al., 2023; Zheng et al., 2021; Zhu et al., 2023) (Supplementary Figures S5A, S6A, S7A, S8A, S9A, S10A). All monomers significantly increased serum E_2_ levels (Supplementary Figures S5B, D). Several monomers also reduced LH levels, led by icariin (SMD = −8.61, 95% CI: −11.15 to −6.08, 95% PI: −13.46 to −3.77; heterogeneity: *I* ^2^ = 63.35%, *P* = 0.065), followed by resveratrol (SMD = −4.28, 95% CI: −6.24 to −2.32, 95% PI: −8.77 to 0.21; heterogeneity: *I* ^2^ = 94.97%, *P* < 0.001), puerarin (SMD = −4.27, 95% CI: −7.13 to −1.40, 95% PI: −9.33 to 0.80; heterogeneity: *I* ^2^ = 0, *P* = 0.846), and quercetin (SMD = −2.04, 95% CI: −3.70 to −0.39, 95% PI: −6.38 to 2.29; heterogeneity: *I* ^2^ = 71.19%, *P* = 0.008) (Supplementary Figures S6B, D). Histopathologically, quercetin and icariin promoted follicular development and mitigated atresia. They restored primordial follicle counts (quercetin: SMD = 4.30, 95% CI: 2.48 to 6.13, 95% PI: 0.97 to 7.64; heterogeneity: *I* ^2^ = 0, *P* = 0.511; icariin: SMD = 1.87, 95% CI: 0.92 to 2.81, 95% PI: −0.77 to 4.50; heterogeneity: *I* ^2^ = 77.19%, *P* = 0.002), with parallel improvements in primary and secondary stages alongside a concurrent reduction in atretic cohorts. However, the wide 95% PIs for most comparisons indicate considerable variability in the expected treatment effects. Cumulative ranking probabilities suggested icariin, astragaloside IV, and quercetin as potential candidate monomers for increasing E_2_ and reducing LH, elevating primordial and primary follicles, and increasing secondary follicles and reducing atretic follicles, respectively (Supplementary Figures S5C, S6C, S7C, S8C, S9C, S10C).

### 3.4 Overall SUCRA ranking

Based on the global mean SUCRA values across all outcomes, quercetin secured the highest overall ranking in the main best-dose network meta-analysis and its corresponding sensitivity analyses. It was therefore selected as the candidate monomer for subsequent pairwise meta-analyses of oxidative stress biomarkers (Figure 4; Supplementary Tables S6-S7). In the additional all-dose network meta-analysis, astragaloside IV ranked first and quercetin ranked second; furthermore, the exploratory sensitivity analysis showed that puerarin ascended from fourth to first place, while astragaloside IV and quercetin adjusted to second and third, respectively. Although the exact ranking varied according to dose handling, quercetin remained among the more highly ranked monomers across the complementary analyses. Taken together with the main analysis findings, quercetin was considered a promising candidate.

**FIGURE 4:**
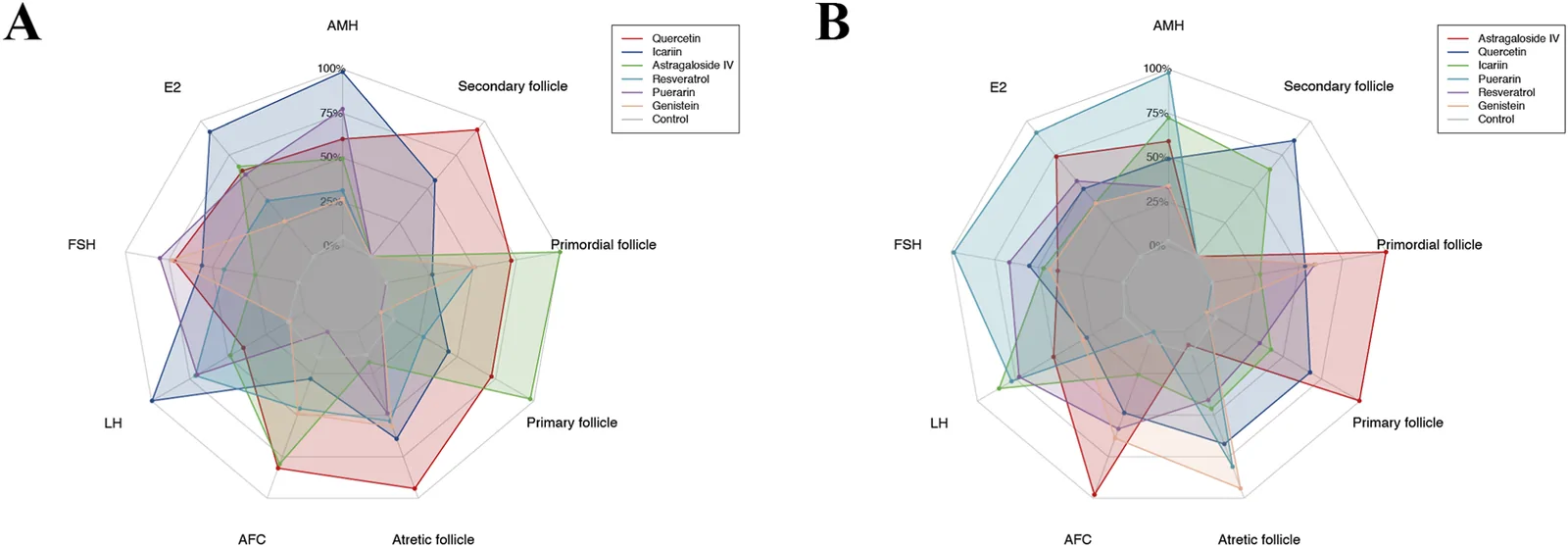
Radar charts of SUCRA values across different dose-selection strategies. **(A)** Radar chart based on the main network meta-analysis, where the best-performing dose arm was selected for each monomer. **(B)** Radar chart based on the additional all-dose network meta-analysis, including all eligible dose arms to assess ranking sensitivity. Each axis represents a distinct ovarian function outcome, and each point indicates its corresponding SUCRA value.

### 3.5 Subgroup and sensitivity analyses

Subgroup analyses revealed distinct efficacy patterns by animal species (mice vs. rats), induction methods (cyclophosphamide (CTX) vs. non-CTX), and treatment duration (21 days or less vs. more than 21 days) (Supplementary Figures S11-S28). Compared with controls, puerarin exhibited the most pronounced effects in mice and non-CTX models, significantly decreasing FSH (mice: SMD = −12.00, 95% CI: −20.30 to −3.70, 95% PI: −26.56 to 2.56; non-CTX: SMD = −12.00, 95% CI: −19.21 to −4.79, 95% PI: −24.39 to 0.39) and elevating AMH (mice: SMD = 15.59, 95% CI: 6.55 to 24.63, 95% PI: −3.57 to 34.76; non-CTX: SMD = 15.59, 95% CI: 7.37 to 23.82, 95% PI: 2.43–28.75). Similarly, icariin demonstrated a prominent FSH-lowering effect in studies with a duration of 21 days or less (SMD = −12.34, 95% CI: −21.27 to −3.41, 95% PI: −28.73 to 4.05), and its AMH-elevating effect remained consistent across treatment durations (21 days or less: SMD = 8.44, 95% CI: 2.46 to 14.41, 95% PI: −1.13 to 18; more than 21 days: SMD = 11.03, 95% CI: 7.14 to 14.92, 95% PI: 2.11–19.95), as well as in CTX (SMD = 10.03, 95% CI: 3.81 to 16.24, 95% PI: 0.59–19.46) and rat models (SMD = 6.62, 95% CI: 2.73 to 10.50, 95% PI: −0.36–13.60). Additionally, quercetin showed substantial efficacy in rat and CTX models, significantly reducing FSH (rats: SMD = −6.02, 95% CI: −8.20 to −3.84, 95% PI: −11.59 to −0.45; CTX: SMD = −5.63, 95% CI: −7.68 to −3.57, 95% PI: −10.88 to −0.37) and increasing AFC (both subgroups: SMD = 5.65, 95% CI: 1.49 to 9.82, 95% PI: −7.11–18.42). Several subgroup-specific comparisons were based on a single study, precluding the estimation of within-comparison heterogeneity; thus, these results should be interpreted with caution. Among comparisons where heterogeneity could be estimated, it remained substantial after stratification, indicating that animal species, induction method, and treatment duration did not fully account for the between-study variability. Sensitivity analysis excluding small-sample studies yielded consistent results (Supplementary Figures S29-S37).

### 3.6 Pairwise meta-analyses of oxidative stress biomarkers

Among the ten studies evaluating quercetin (Eren et al., 2024; Guo et al., 2024; Huang et al., 2020; Ji et al., 2025; Jiang et al., 2025; Li, 2013; Mai et al., 2025; Moradi et al., 2023; Wang et al., 2025; Zheng et al., 2021), eight investigated mechanisms (Guo et al., 2024; Ji et al., 2025; Jiang et al., 2025; Li, 2013; Mai et al., 2025; Moradi et al., 2023; Wang et al., 2025; Zheng et al., 2021), and six assessed oxidative stress biomarkers (Guo et al., 2024; Jiang et al., 2025; Li, 2013; Mai et al., 2025; Wang et al., 2025; Zheng et al., 2021) (Table 2). Pooled results revealed that quercetin increased serum SOD (SMD = 5.90, 95% CI: 2.43 to 9.38, 95% PI: −5.66 to 17.47; heterogeneity: *I* ^2^ = 86.6%, *P* < 0.001) and CAT (SMD = 4.48, 95% CI: 2.45 to 6.51, 95% PI: −1.80 to 10.76; heterogeneity: *I* ^2^ = 64.1%, *P* = 0.039), while reducing serum MDA levels (SMD = −2.88; 95% CI: −4.08 to −1.69, 95% PI: −6.29 to 0.52; heterogeneity: *I* ^2^ = 63.4%, *P* = 0.018) (Figure 5). Additionally, one study (Wang et al., 2025) supported the upregulation of serum GPx (SMD = 1.29, 95% CI: 0–2.58). Beyond serum markers, two studies (Li, 2013; Zheng et al., 2021) reported improved levels of these four biomarkers in ovarian and hepatic tissues, respectively. While these findings demonstrated a correlation between quercetin and favorable alterations in oxidative stress biomarkers, the observed benefits should not be solely attributed to oxidative stress modulation.

**TABLE 2:** Characteristics of studies evaluating the effects of quercetin on oxidative stress biomarkers.

Study ID (first author, year)	Species (weight, age)	Modeling induction drug (dosage, approach, frequency, duration)	Sample sizes (T/M)	Quercetin (dosage, approach, frequency, duration)	Oxidative stress markers
Jiang et al., 2025	Sprague-Dawley rats (170 ± 10 g, 8 weeks)	Cyclophosphamide (D1: 50 mg/kg, D2**–**D7: 5 mg/kg; i.p., qd, 7 days)	TL: 10/TM: 10/TH: 10/M: 10	TL: 25 mg/kg, i.g., qd, 30 days TM: 50 mg/kg, i.g., qd, 30 days TH: 100 mg/kg, i.g., qd, 30 days	SOD, CAT, MDA in serum
Guo et al., 2024	Sprague-Dawley rats (210 ± 20 g, NR)	Cyclophosphamide (D1: 50 mg/kg, D2**–**D15: 8 mg/kg; i.p., qd, 15 days)	TL: 12/TH: 12/M: 12	TL: 25 mg/kg, i.g., qd, 14 days TH: 100 mg/kg, i.g., qd, 14 days	SOD, MDA in serum
Li, 2013	BALB/c mice (21**–**23 g, 7**–**9 weeks)	Peptide zona pellucida 3 (D0: 0.15mL, CFA-pZP3, D14: 0.15mL, IFA-pZP3; s.c., D0, D14, 14 days)	TL: 12/TM: 12/TH: 12/M: 12	TL: 5 mg/kg, i.g., qd, 50 days TM: 25 mg/kg, i.g., qd, 50 days TH: 45 mg/kg, i.g., qd, 50 days	SOD, CAT, MDA, GPx in liver
Mai et al., 2025	C57BL/6 mice (20 ± 5 g, 5 weeks)	Cyclophosphamide (90 mg/kg, i.p., once, 1 day)	T: 10/M: 10	50 mg/kg, i.g., qd, 21 days	SOD, CAT in serum
Zheng et al., 2021	Sprague-Dawley rats (260 ± 20 g, 12 weeks)	Cyclophosphamide (100 mg/kg, i.p., once, 1 day)	TL: 10/TM: 10/TH: 10/M: 10	TL: 25 mg/kg, i.g., qd, 30 days TM: 50 mg/kg, i.g., qd, 30 days TH: 100 mg/kg, i.g., qd, 30 days	SOD, CAT, MDA, GPx in ovary
Wang et al., 2025	Sprague-Dawley rats (200 g, 8 weeks)	Cyclophosphamide (D1: 200 mg/kg, D2**–**D15: 8 mg/kg; i.p., qd, 15 days)	T: 6/M: 6	100 mg/kg, i.g., qd, 30 days	SOD, MDA, GPx in serum

M: model group; T: treatment group; TL: treatment group with low-dose quercetin; TM: treatment group with medium-dose quercetin; TH: treatment group with high-dose quercetin; SOD: superoxide dismutase; CAT: catalase; MDA: malondialdehyde; GPx: glutathione peroxidase; i. g.: intragastric administration; i. p.: intraperitoneal injection; s. c.: subcutaneous injection; qd: once daily; NR: not reported.

**FIGURE 5:**
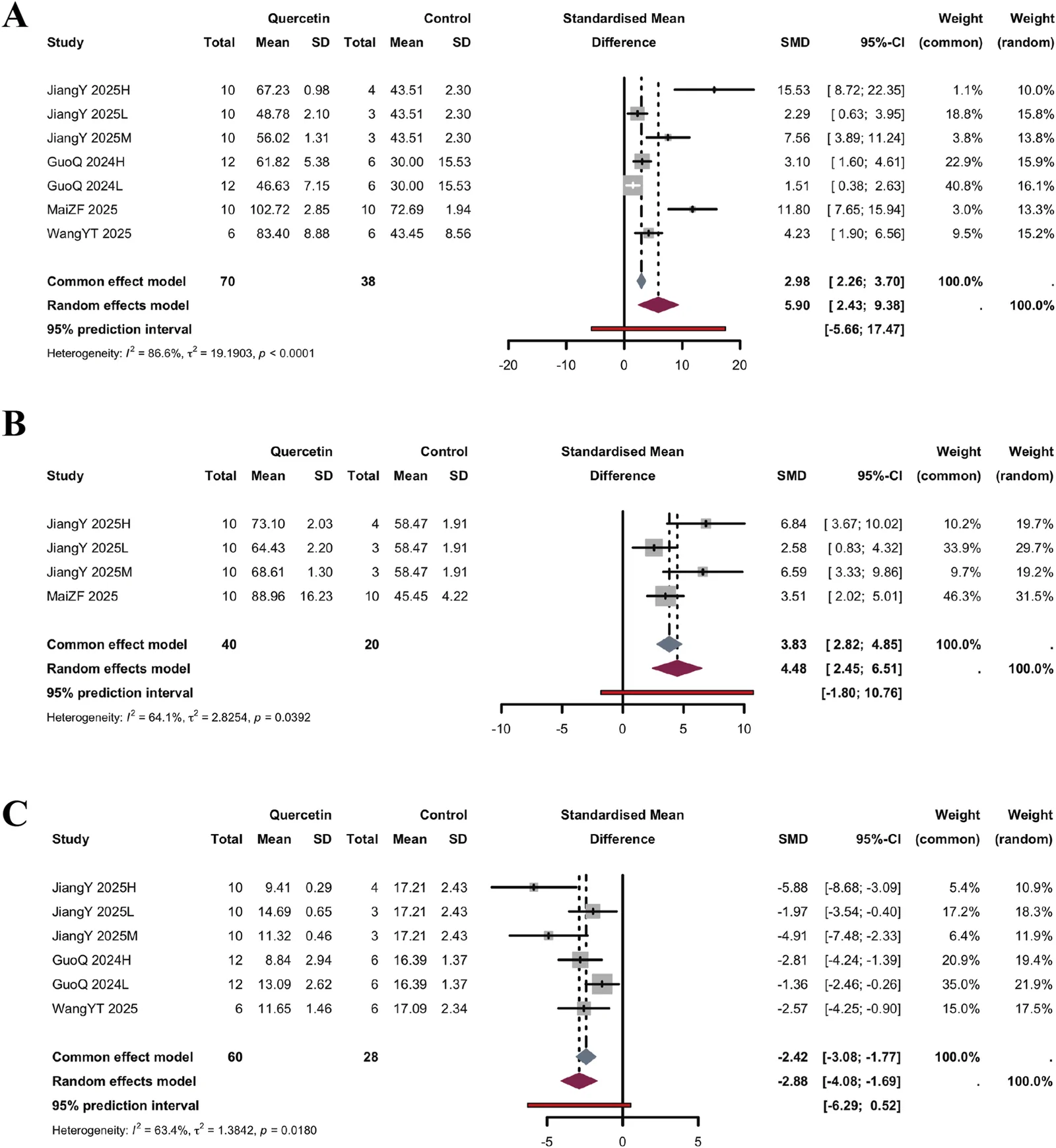
Forest plots of the effects of quercetin on serum oxidative stress biomarkers. **(A)** Superoxide dismutase (SOD); **(B)** Catalase (CAT); **(C)** Malondialdehyde (MDA).

### 3.7 Exploratory three-dimensional scatter plots

Three-dimensional scatter plots illustrate the primary outcomes effect estimates of quercetin across all observed doses and treatment durations (Figure 6). Although the majority of data points clustered in the low-dose and short-duration regions, quercetin showed an exploratory trend toward reduced FSH alongside increased AMH and AFC at doses of 25–100 mg/kg over 10–30 days. As noted, these results were descriptive and did not establish to a formal dose-response relationship.

**FIGURE 6:**
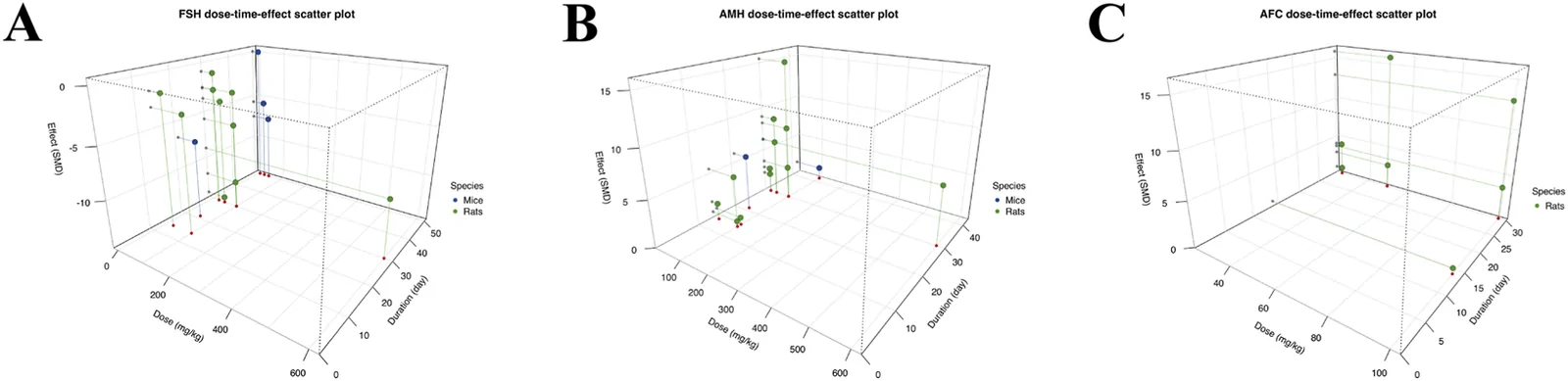
Exploratory three-dimensional scatter plots of quercetin on primary outcomes in POI models. **(A)** FSH dose–time–effect scatter plot; **(B)** AMH dose–time–effect scatter plot; **(C)** AFC dose–time–effect scatter plot.

## 4 Discussion

### 4.1 Principal findings

This network meta-analysis evaluated the efficacy of six TCM monomers—quercetin, icariin, resveratrol, astragaloside IV, genistein, and puerarin—against controls in POI animal models. All six monomers consistently reduced FSH, with four elevating AMH and two increasing AFC. Notably, quercetin and icariin demonstrated substantial advantages by restoring E_2_ and LH, promoting follicular development and suppressing atresia. Given that the overall certainty of evidence was low to very low, these findings should be interpreted cautiously. Although dose-selection strategies slightly influenced the exact overall rankings, quercetin ranked highest in the main analysis and was selected for subsequent pairwise meta-analyses on oxidative stress biomarkers. These analyses showed that quercetin increased serum SOD and CAT levels and reduced MDA levels, suggesting that the regulation of oxidative stress may be associated with its protective effects on ovarian function. Furthermore, three-dimensional scatter plots revealed that quercetin exhibits a potential enhanced therapeutic trend in regulating FSH, AMH, and AFC levels within the dosage range of 25–100 mg/kg for 10–30 days.

### 4.2 Interpretation of heterogeneity, evidence certainty, and ranking stability

These apparent efficacy advantages should be interpreted in light of the substantial heterogeneity and low certainty of evidence. Heterogeneity was high across the main networks and remained substantial in several subgroup analyses. In addition, several 95% PIs were wide and crossed the null value, indicating that both the direction and magnitude of effects may vary across future comparable experimental settings. This variability may reflect differences in animal characteristics, modeling methods, treatment doses, sample sizes, and the incomplete reporting of randomization and blinding. Moreover, the overall certainty of evidence was constrained by methodological shortcomings in the included preclinical studies. Accordingly, the SUCRA rankings should be regarded as hypothesis-generating rather than definitive estimates of comparative efficacy.

Dose selection is a critical consideration when interpreting SUCRA rankings (Kwon et al., 2026). Although the best-dose strategy used in the main network meta-analysis prevented disproportionate weighting from multiple dose arms within a study, it may have preferentially retained extreme effects and thereby inflated efficacy estimates. Despite fluctuations in relative rankings across the all-dose analysis, quercetin remained among the higher-ranked monomers in complementary analyses. This relative stability provides additional rationale for prioritizing quercetin for mechanistic investigation, highlighting its potential as a promising candidate.

### 4.3 Potential mechanisms of quercetin

As the most extensively documented monomer in this study, quercetin was evaluated across all outcomes and exhibited a favorable trend in follicle count restoration. Pooled biomarker analyses further suggested that modulation of oxidative stress may contribute to its ovarian-protective effects. Structurally, quercetin is a polyhydroxylated flavonol containing a catechol B ring, a configuration that may facilitate radical scavenging and redox regulation (Corrente et al., 2025; Heřmánková et al., 2019). In line with its established antioxidant capacity (Shen et al., 2013; Lan et al., 2022; Roslan et al., 2017), quercetin may help preserve the follicular microenvironment by reinforcing antioxidant defenses and mitigating lipid peroxidation. This interpretation is biologically plausible because excessive ROS can promote POI progression through GC apoptosis and follicular atresia (Guo et al., 2025), while quercetin has been reported to protect GCs from oxidative injury (Qi et al., 2023) and ameliorate oxidative stress and hormonal disturbances in reproductive injury models (Nna et al., 2017).

It is important to note that the observed ovarian effects should not be attributed solely to antioxidant mechanisms. Given the pleiotropic pharmacology of quercetin (Zhang et al., 2025), its effects on oxidative stress may involve other signaling pathways, including SDF-1/CXCR4 (Guo et al., 2024), PI3K/Akt/FoxO3a (Zheng et al., 2021), JAK2/STAT3 (Jiang et al., 2025) and SLC7A11/GPX4/ACSL4 (Mai et al., 2025) (Figure 7). These pathways could collectively influence follicular development, hormone secretion, and ovarian reserve. Because evidence for other mechanisms was insufficient for quantitative synthesis, the present meta-analysis focused on oxidative stress biomarkers while underscoring the need for integrated mechanistic studies.

**FIGURE 7:**
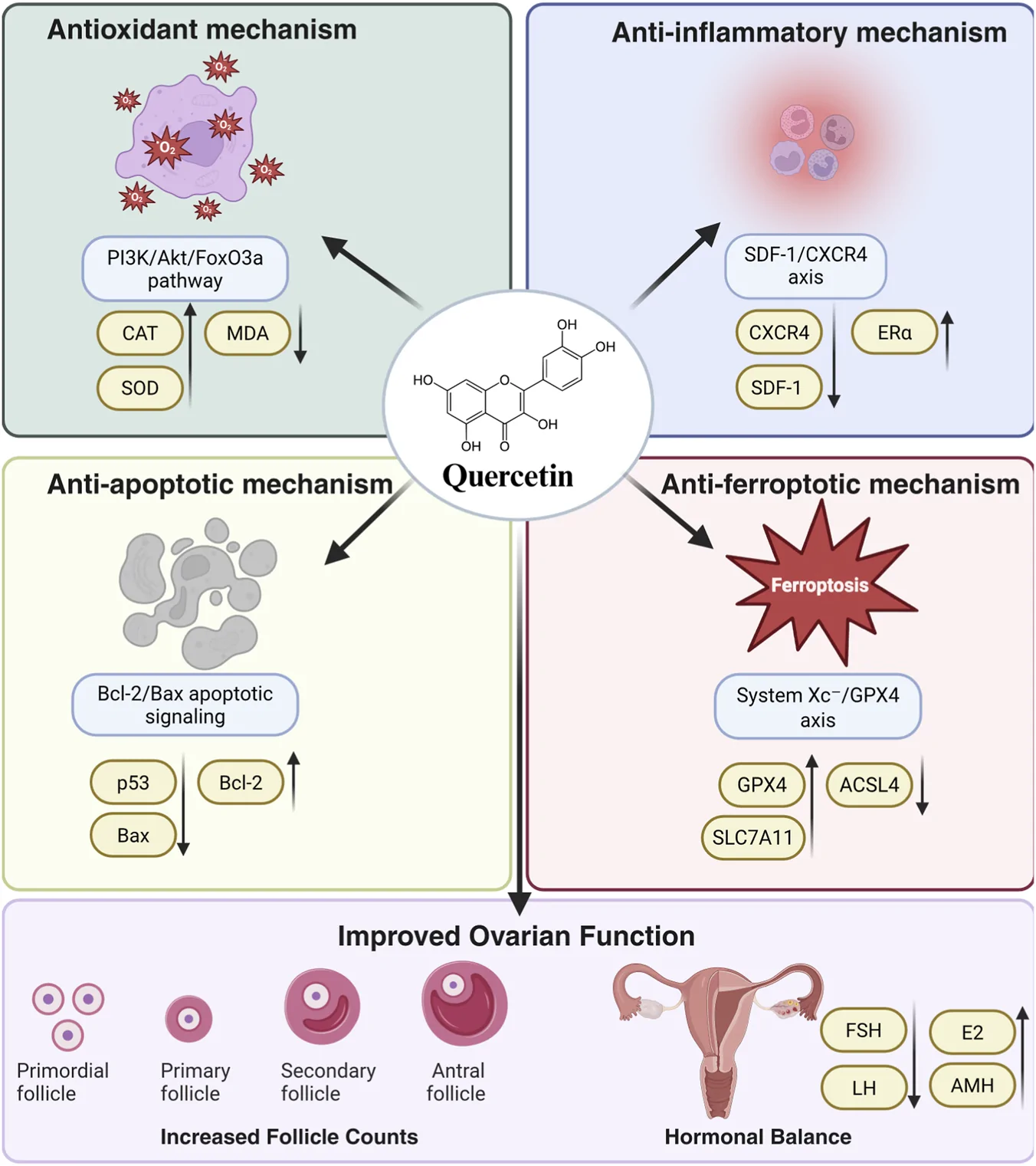
The potential mechanisms of quercetin in POI models. Created in BioRender (https://BioRender.com/p4ocfo1).

### 4.4 Potential mechanisms of other TCM monomers

Beyond quercetin, the SUCRA analyses suggested outcome-specific advantages for several other TCM monomers, although the evidence supporting these rankings remains limited. Puerarin, a C-glycosylated isoflavone and the major bioactive constituent of *Pueraria lobata*, ranked highest for lowering FSH. It has been reported to alleviate ovarian oxidative stress and maintain hormonal homeostasis, thereby ameliorating reproductive aging (Huang et al., 2026). Icariin, a prenylated and glycosylated flavonol, ranked highest for increasing AMH and E_2_ levels and decreasing LH. It may promote estrogen biosynthesis through aromatase upregulation (Yang et al., 2013) and mitigate ovarian toxicity through antioxidant, anti-inflammatory, and anti-apoptotic effects (Eid et al., 2025). Astragaloside IV, a triterpenoid saponin derived from *Astragalus membranaceus*, ranked highest for increasing primordial and primary follicles. It may preserve the ovarian microenvironment by attenuating tissue damage, enhancing antioxidant defenses, and suppressing pro-inflammatory cytokines (Feng et al., 2026). Collectively, these insights suggest that differences in chemical structures may contribute to distinct efficacy patterns through interconnected mechanisms involving oxidative stress, inflammation, apoptosis and autophagy.

### 4.5 Exploratory visualization of quercetin therapeutic trends

Building on previous findings regarding the favorable effects of quercetin on hormone levels, follicular development, and oxidative stress biomarkers, we further explored the potential influence of dose and treatment duration on the primary outcomes. The three-dimensional scatter plots showed a gradual trend toward greater effects at higher doses within the range of 25–100 mg/kg over 10–30 days. This pattern represents a preliminary descriptive signal rather than evidence of a definitive dose-dependent effect. The limited number of eligible studies, uneven distribution of dose-duration regimens, and differences in induction methods precluded reliable meta-regression. Accordingly, these findings cannot be used to determine an appropriate human dose. Although quercetin has been evaluated in other conditions at 150 mg/day for 6 weeks (Egert et al., 2009), 500 mg/day for 8 weeks (Javadi et al., 2017), and 1000 mg/day for 12 weeks (Rezvan et al., 2017), these studies do not establish its efficacy, safety, or optimal regimen in POI because of distinct disease mechanisms. Potential adverse effects associated with high doses or prolonged treatment also warrant caution (Andres et al., 2018). Well-designed dose-response studies are therefore needed to define an appropriate therapeutic window for POI.

### 4.6 Translational challenges

Despite these encouraging preclinical findings, several barriers remain to the clinical translation of TCM monomers. The available evidence is largely derived from acute POI models, which cannot fully recapitulate the etiological heterogeneity or long-term course of clinical POI. Species differences in pharmacokinetics also complicate dose extrapolation from rodents to humans. Moreover, the included studies provided limited systematic information on toxicity and safety. For quercetin, poor aqueous solubility and limited oral bioavailability may further restrict systemic exposure, representing an additional challenge for clinical translation (Liu et al., 2025).

### 4.7 Study limitations

This study has several limitations. Restricting the literature search to mainstream English and Chinese databases may have introduced language bias. Small sample sizes, frequently unclear risk of bias, and substantial heterogeneity across animal models may have compromised the robustness of the pooled estimates, while the very low certainty of evidence for most comparisons and potential publication bias for several outcomes reduce confidence in the findings. Requiring at least three studies per monomer improved network stability but excluded emerging candidates, and rankings based on sparse outcome data should be interpreted cautiously. Additionally, assigning a value of zero to missing SUCRA values in the calculation of the global mean SUCRA was a conservative approach that may have disadvantaged interventions with incomplete outcome data. Selecting the best-performing dose for each monomer in the main network meta-analysis may also have introduced selection bias and inflated effect estimates. The three-dimensional scatter plot analysis was exploratory because formal dose-response meta-regression was not feasible with the available data. Finally, although our results support the antioxidant activity of quercetin, existing studies have focused predominantly on oxidative stress biomarkers, leaving its broader regulatory mechanisms insufficiently explored.

### 4.8 Future directions

Future studies should prioritize adequately powered preclinical dose-response studies using standardized POI induction protocols and outcome definitions, prespecified doses and treatment durations, rigorous randomization and blinding, and complete data reporting. These studies should include pharmacokinetic, toxicological, reproductive safety, dose translation, and time-course assessments, as well as clinically relevant fertility outcomes such as pregnancy and live birth rates. Integrating transcriptomic, proteomic, and metabolomic profiling with pathway-specific validation could clarify the interconnected mechanisms underlying the effects of TCM monomers. Systematic evaluation of novel monomer structures and analogue series may further reveal relationships between chemical features and efficacy, while advanced delivery systems could improve bioavailability. Ultimately, well-designed randomized controlled trials conducted within established therapeutic windows will be required to assess the clinical utility of these monomers in POI management.

## 5 Conclusion

To the best of our knowledge, this is the first network meta-analysis to evaluate the effects of TCM monomers on POI, demonstrating that several monomers improved hormone levels and follicular development in animal models. Notably, quercetin ranked highest in the main analysis and emerged as a promising candidate overall, with favorable changes in oxidative stress biomarkers. While these exploratory findings offer preliminary support for the therapeutic potential of TCM monomers, they should be interpreted with caution due to substantial heterogeneity and very low certainty of evidence. Future studies should use standardized models and dose-response designs to clarify effective and safe dose ranges, and apply structure-activity relationship analyses to guide the design of monomer derivatives with potentially improved efficacy, bioavailability, and safety. Integrated multi-omics studies and well-designed clinical trials are needed to validate these preclinical findings and advance the clinical translation of promising TCM monomers for POI management.

## Data Availability

The original contributions presented in the study are included in the article/Supplementary Material, further inquiries can be directed to the corresponding author.
