# Supplementary material for: Screening traditional Chinese medicine monomers for premature ovarian insufficiency: from network meta-analysis to the effects of a promising candidate on oxidative stress biomarkers
Source: Front Pharmacol. 2026 Sep 18;17:1934959. doi: 10.3389/fphar.2026.1934959 (PMC13655582; doi:10.3389/fphar.2026.1934959)
Supplement: Supplementary file 1 [file Supplementaryfile1.pdf]

Supplementary Material 1

Table S1 Detailed search strategies for electronic databases.
Table S2 Risk of bias assessment using the SYRCLE's RoB tool.
Table S3 CINeMA assessment of confidence in network meta-analysis evidence.
Table S4 Global heterogeneity for outcomes across subgroup and sensitivity analyses.
Table S5 Pairwise heterogeneity for all outcome comparisons.
Table S6 SUCRA values of different interventions across various outcomes.
Table S7 Summary of overall efficacy rankings and comparison between best-dose and all-dose network meta-analyses for different interventions.
Figure S1 Comparison-adjusted funnel plots for FSH, AMH, and E_2.
Figure S2 Comparison-adjusted funnel plots for LH, primordial follicle, primary follicle and atretic follicle.
Figure S3 Network meta-analysis of AMH levels.
Figure S4 Network meta-analysis of AFC levels.
Figure S5 Network meta-analysis of E_2 levels.
Figure S6 Network meta-analysis of LH levels.
Figure S7 Network meta-analysis of primordial follicles.
Figure S8 Network meta-analysis of primary follicles.
Figure S9 Network meta-analysis of secondary follicles.
Figure S10 Network meta-analysis of atretic follicles.
Figure S11–S16 Subgroup analysis of FSH levels.
Figure S17–S22 Subgroup analysis of AMH levels.
Figure S23–S28 Subgroup analysis of AFC.
Figure S29–S37 Sensitivity analysis excluding small-sample studies.

Table S1 Detailed search strategies for electronic databases.

English databases (PubMed, Embase, the Cochrane Library, Web of Science):	
#1	Primary Ovarian Insufficiency [MeSH Terms]
#2	Menopause, Premature [MeSH Terms]
#3	premature ovarian insufficiency [Title/Abstract]
#4	premature ovarian failure [Title/Abstract]
#5	POI [Title/Abstract]
#6	POF [Title/Abstract]
#7	#1 OR #2 OR #3 OR #4 OR #5 OR #6
#8	monomer [Title/Abstract]
#9	isolated compound [Title/Abstract]
#10	active component [Title/Abstract]
#11	traditional Chinese medicine extract [Title/Abstract]
#12	Flavonoids [MeSH Terms]
#13	Saponins [MeSH Terms]
#14	Polysaccharides [MeSH Terms]
#15	Alkaloids [MeSH Terms]
#16	Polyphenols [MeSH Terms]
#17	Terpenes [MeSH Terms]
#18	Glycosides [MeSH Terms]
#19	icariin [Title/Abstract]
#20	Quercetin [MeSH Terms]
#21	baicalein [Title/Abstract]
#22	Curcumin [MeSH Terms]
#23	Resveratrol [MeSH Terms]
#24	Ginsenoside [MeSH Terms]
#25	tanshinone [Title/Abstract]
#26	puerarin [Title/Abstract]
#27	kaempferol [Title/Abstract]
#28	#8 OR #9 OR #10 OR #11 OR #12 OR #13 OR #14 OR #15 OR #16 OR #17 OR #18 OR #19 OR #20 OR #21 OR #22 OR #23 OR #24 OR #25 OR #26 OR #27
#29	Animals [MeSH Terms]
#30	Mice [MeSH Terms]
#31	Rats [MeSH Terms]
#32	animal model [Title/Abstract]
#33	mouse [Title/Abstract]
#34	experiment [Title/Abstract]
#35	#29 OR #30 OR #31 OR #32 OR #33 OR #34
#36	#7 AND #28 AND #35

Chinese databases (CNKI, VIP, WangFang, Sinomed):	
#1	早发性卵巢功能不全[篇关摘]
#2	卵巢早衰[篇关摘]
#3	卵巢功能衰退[篇关摘]
#4	POI[篇关摘]
#5	POF[篇关摘]
#6	#1 OR #2 OR #3 OR #4 OR #5
#7	中药单体[篇关摘]
#8	有效成分[篇关摘]
#9	活性成分[篇关摘]
#10	中药提取物[篇关摘]
#11	黄酮[篇关摘]
#12	皂苷[篇关摘]
#13	多糖[篇关摘]
#14	生物碱[篇关摘]
#15	多酚[篇关摘]
#16	萜类[篇关摘]
#17	苷[篇关摘]
#18	淫羊藿苷[篇关摘]
#19	槲皮素[篇关摘]
#20	黄芩素[篇关摘]
#21	姜黄素[篇关摘]
#22	白藜芦醇[篇关摘]
#23	人参皂苷[篇关摘]
#24	丹参酮[篇关摘]
#25	葛根素[篇关摘]
#26	山奈酚[篇关摘]
#27	#7 OR #8 OR #9 OR #10 OR #11 OR #12 OR #13 OR #14 OR #15 OR #16 OR #17 OR #18 OR #19 OR #20 OR #21 OR #22 OR #23 OR #24 OR #25 OR #26
#28	大鼠[篇关摘]
#29	小鼠[篇关摘]
#30	实验[篇关摘]
#31	模型[篇关摘]
#32	体内[篇关摘]
#33	动物[篇关摘]
#34	#28 OR #29 OR #30 OR #31 OR #32 OR #33
#35	#6 AND #27 AND #34

Table S2 Risk of bias assessment using the SYRCLE's RoB tool.

Study ID	Q1	Q2	Q3	Q4	Q5	Q6	Q7	Q8	Q9	Q10
LiN 2018	L	L	U	U	U	U	U	L	L	U
ChenYQ 2025	L	L	U	L	U	U	U	L	L	L
ZhouYF 2024	U	L	U	L	U	U	U	L	L	L
ZhangHY 2021	U	L	U	L	U	U	U	L	L	L
DingXL 2025	U	U	U	L	U	U	U	L	L	L
JiangY 2025	L	U	U	L	U	U	U	L	L	U
GuoQ 2024	U	U	U	U	U	U	U	L	L	L
LiN 2013	U	L	U	L	U	U	U	U	L	L
MaiZF 2025	U	L	U	L	U	U	U	L	L	U
YangL 2024	U	U	U	L	U	U	U	L	L	L
XingSS 2023	U	U	U	U	U	U	U	L	L	L
ChenHR 2022	L	L	U	L	U	U	U	L	L	L
LiYX 2025	U	L	U	L	U	U	U	L	L	L
AiH 2007	U	U	U	U	U	U	U	L	U	U
HuangCS 2020	U	U	U	U	U	U	U	L	L	L
ZhuBL 2023	U	L	U	L	U	U	U	L	L	L
XueMZ 2023	U	L	U	L	U	U	U	L	L	L
TongRX 2019	L	U	U	L	U	U	U	L	L	U
MobasherMA 2023	U	L	U	L	U	U	U	L	L	U
ErenCY 2024	U	L	U	U	U	U	U	L	L	U
ZhengSY 2021	U	L	U	U	U	U	U	L	L	U
ÖzatikFY 2016	U	L	U	U	U	U	U	L	L	U
LiFF 2024	U	U	U	L	U	U	U	L	L	L
YangJJ 2026	L	U	U	L	U	U	U	L	L	L
LiN 2019	U	U	U	U	U	U	U	U	L	U
WangYT 2025	U	L	U	L	U	U	U	L	L	U
YuZY 2026	U	U	U	L	U	U	U	L	L	U
HaddadYH 2020	U	L	U	U	U	U	U	U	U	U
FangYJ 2025	U	L	U	L	U	U	U	U	L	L
SaidRS 2016	U	L	U	L	U	U	U	U	U	U
JiM 2024	U	L	U	U	U	U	U	L	U	U
MoradiS 2023	U	L	U	U	U	U	U	L	U	U

Q1: Sequence generation; Q2: Baseline characteristics; Q3: Allocation concealment; Q4: Random housing; Q5: Blinding of caregivers and researchers; Q6: Random outcome assessment; Q7: Blinding of outcome assessors; Q8: Incomplete outcome data; Q9: Selective outcome reporting; Q10: Other sources of bias; L: Low risk; U: Unclear risk.

Table S3 CINeMA assessment of confidence in network meta-analysis evidence.

Outcome	Comparison	*k*	D1	D2	D3	D4	D5	D6	Confidence rating	Reason(s) for downgrading
FSH	Astragaloside IV:Control	3	Some concerns	Low risk	No concerns	No concerns	Major concerns	Major concerns	Very low	Risk of bias, substantial heterogeneity and incoherence
FSH	Control:Genistein	1	Some concerns	Low risk	Some concerns	No concerns	Major concerns	No concerns	Very low	Risk of bias, indirectness and substantial heterogeneity
FSH	Control:Icariin	5	Some concerns	Some concerns	No concerns	No concerns	Major concerns	Major concerns	Very low	Risk of bias, suspected publication bias, substantial heterogeneity and incoherence
FSH	Control:Puerarin	3	Some concerns	Some concerns	No concerns	No concerns	No concerns	Major concerns	Very low	Risk of bias, suspected publication bias and incoherence
FSH	Control:Quercetin	7	Some concerns	Low risk	No concerns	No concerns	No concerns	Major concerns	Very low	Risk of bias and incoherence

FSH	Control:Resveratrol	5	Some concerns	Some concerns	No concerns	No concerns	Major concerns	Major concerns	Very low	Risk of bias, suspected publication bias, substantial heterogeneity and incoherence
AMH	Astragaloside IV:Control	3	Some concerns	Low risk	No concerns	No concerns	Major concerns	Major concerns	Very low	Risk of bias, substantial heterogeneity and incoherence
AMH	Control:Genistein	1	Some concerns	Low risk	Some concerns	Major concerns	No concerns	Major concerns	Very low	Risk of bias, indirectness, sparse network and incoherence
AMH	Control:Icariin	5	Some concerns	Low risk	No concerns	No concerns	No concerns	Major concerns	Very low	Risk of bias and incoherence
AMH	Control:Puerarin	3	Some concerns	Some concerns	No concerns	No concerns	Some concerns	Major concerns	Very low	Risk of bias, suspected publication bias, moderate variance and incoherence
AMH	Control:Quercetin	9	Some concerns	Some concerns	No concerns	No concerns	Major concerns	Major concerns	Very low	Risk of bias, suspected publication bias, substantial heterogeneity and incoherence
AMH	Control:Resveratrol	4	Some concerns	Low risk	No concerns	Major concerns	No concerns	Major concerns	Very low	Risk of bias, sparse network and incoherence

AFC	Astragaloside IV:Control	2	Some concerns	Low risk	No concerns	No concerns	Major concerns	Major concerns	Very low	Risk of bias, substantial heterogeneity and incoherence
AFC	Control:Genistein	1	Some concerns	Low risk	Some concerns	Major concerns	No concerns	Major concerns	Very low	Risk of bias, indirectness, sparse network and incoherence
AFC	Control:Icariin	2	Some concerns	Low risk	No concerns	Major concerns	No concerns	Major concerns	Very low	Risk of bias, sparse network and incoherence
AFC	Control:Quercetin	3	Some concerns	Low risk	No concerns	No concerns	Major concerns	Major concerns	Very low	Risk of bias, substantial heterogeneity and incoherence
AFC	Control:Resveratrol	1	Some concerns	Low risk	Some concerns	Major concerns	No concerns	Major concerns	Very low	Risk of bias, indirectness, sparse network and incoherence
E_2	Astragaloside IV:Control	3	Some concerns	Low risk	No concerns	No concerns	Some concerns	Major concerns	Very low	Risk of bias, moderate variance and incoherence
E_2	Control:Genistein	3	Some concerns	Low risk	Some concerns	No concerns	Major concerns	No concerns	Very low	Risk of bias, indirectness and substantial heterogeneity

E_2	Control:Icariin	4	Some concerns	Low risk	No concerns	No concerns	No concerns	Major concerns	Very low	Risk of bias and incoherence
E_2	Control:Puerarin	3	Some concerns	Low risk	No concerns	No concerns	Major concerns	Major concerns	Very low	Risk of bias, substantial heterogeneity and incoherence
E_2	Control:Quercetin	9	Some concerns	Some concerns	No concerns	No concerns	No concerns	No concerns	Low	Risk of bias and suspected publication bias
E_2	Control:Resveratrol	6	Some concerns	Some concerns	Some concerns	No concerns	Major concerns	Major concerns	Very low	Risk of bias, suspected publication bias, indirectness, substantial heterogeneity and incoherence
LH	Astragaloside IV:Control	2	Some concerns	Low risk	No concerns	Some concerns	Some concerns	Major concerns	Very low	Risk of bias, wide confidence intervals, moderate variance and incoherence
LH	Control:Genistein	1	Some concerns	Low risk	Some concerns	Major concerns	No concerns	No concerns	Very low	Risk of bias, indirectness and sparse network

LH	Control:Icariin	3	Some concerns	Some concerns	No concerns	No concerns	No concerns	Major concerns	Very low	Risk of bias, suspected publication bias and incoherence
LH	Control:Puerarin	2	Some concerns	Low risk	No concerns	No concerns	Major concerns	Major concerns	Very low	Risk of bias, substantial heterogeneity and incoherence
LH	Control:Quercetin	5	Some concerns	Some concerns	No concerns	No concerns	Major concerns	Major concerns	Very low	Risk of bias, suspected publication bias, substantial heterogeneity and incoherence
LH	Control:Resveratrol	4	Some concerns	Some concerns	No concerns	No concerns	Major concerns	Major concerns	Very low	Risk of bias, suspected publication bias, substantial heterogeneity and incoherence
Primordial follicle	Astragaloside IV:Control	1	Some concerns	Low risk	No concerns	No concerns	No concerns	Major concerns	Very low	Risk of bias and incoherence
Primordial follicle	Control:Genistein	1	Some concerns	Low risk	Some concerns	No concerns	Major concerns	Major concerns	Very low	Risk of bias, indirectness, substantial heterogeneity and incoherence

Primordial follicle	Control:Icariin	5	Some concerns	Some concerns	Some concerns	No concerns	Major concerns	Major concerns	Very low	Risk of bias, suspected publication bias, indirectness, substantial heterogeneity and incoherence
Primordial follicle	Control:Quercetin	2	Some concerns	Low risk	No concerns	No concerns	No concerns	Major concerns	Very low	Risk of bias and incoherence
Primordial follicle	Control:Resveratrol	1	Some concerns	Low risk	Some concerns	No concerns	Major concerns	Major concerns	Very low	Risk of bias, indirectness, substantial heterogeneity and incoherence
Primary follicle	Astragaloside IV:Control	2	Some concerns	Some concerns	No concerns	No concerns	No concerns	Major concerns	Very low	Risk of bias, suspected publication bias and incoherence
Primary follicle	Control:Icariin	4	Some concerns	Low risk	No concerns	No concerns	Major concerns	Major concerns	Very low	Risk of bias, substantial heterogeneity and incoherence
Primary follicle	Control:Quercetin	4	Some concerns	Some concerns	Some concerns	No concerns	Major concerns	Major concerns	Very low	Risk of bias, suspected publication bias, indirectness, substantial heterogeneity and incoherence

Primary follicle	Control:Resveratrol	1	No concerns	Low risk	No concerns	Major concerns	No concerns	Major concerns	Very low	Sparse network and incoherence
Secondary follicle	Control:Icariin	4	Some concerns	Low risk	Some concerns	No concerns	Major concerns	Major concerns	Very low	Risk of bias, indirectness, substantial heterogeneity and incoherence
Secondary follicle	Control:Quercetin	3	Some concerns	Low risk	No concerns	No concerns	Major concerns	Major concerns	Very low	Risk of bias, substantial heterogeneity and incoherence
Atretic follicle	Astragaloside IV:Control	1	Some concerns	Low risk	No concerns	Major concerns	No concerns	Major concerns	Very low	Risk of bias, sparse network and incoherence
Atretic follicle	Control:Genistein	1	Some concerns	Low risk	Some concerns	Major concerns	No concerns	Major concerns	Very low	Risk of bias, indirectness, sparse network and incoherence
Atretic follicle	Control:Icariin	3	Some concerns	Some concerns	No concerns	No concerns	Major concerns	Major concerns	Very low	Risk of bias, suspected publication bias, substantial heterogeneity and incoherence
Atretic follicle	Control:Puerarin	2	Some concerns	Low risk	No concerns	Major concerns	No concerns	Major concerns	Very low	Risk of bias, sparse network and incoherence

Atretic follicle	Control:Quercetin	4	Some concerns	Some concerns	No concerns	No concerns	Major concerns	Major concerns	Very low	Risk of bias, suspected publication bias, substantial heterogeneity and incoherence
Atretic follicle	Control:Resveratrol	3	Some concerns	Some concerns	No concerns	Major concerns	No concerns	Major concerns	Very low	Risk of bias, suspected publication bias, sparse network and incoherence

Overall confidence was rated as high, moderate, low, or very low according to the CINeMA framework. D1: Within-study bias; D2: Reporting bias; D3: Indirectness; D4: Imprecision; D5: Heterogeneity; D6: Incoherence; *k*: Number of studies; FSH: follicle-stimulating hormone; AMH: anti-Müllerian hormone; AFC: antral follicle count; E_2: estradiol; LH: luteinizing hormone.

Table S4 Global heterogeneity for outcomes across subgroup and sensitivity analyses.

Outcome and Network Structure	Number of studies	*Q* statistic	*P*-value	τ^2	I^2 (%)
FSH					
Main network	23	182.746	<0.001	6.116	90.15
Subgroup: mice	9	95.084	<0.001	14.130	94.74
Subgroup: rats	14	70.075	<0.001	4.826	87.16
Subgroup: CTX	14	64.258	<0.001	4.299	85.99
Subgroup: non-CTX	9	68.366	<0.001	9.723	92.69
Subgroup: more than 21 days	15	118.772	<0.001	5.655	90.74
Subgroup: 21 days or less	8	41.570	<0.001	14.087	90.38
Sensitivity network	23	182.746	<0.001	6.116	90.15
AMH					
Main network	25	237.960	<0.001	7.992	92.02
Subgroup: mice	7	27.616	<0.001	14.989	89.14
Subgroup: rats	18	127.072	<0.001	6.340	90.56
Subgroup: CTX	14	105.331	<0.001	7.340	91.46
Subgroup: non-CTX	11	107.991	<0.001	11.327	94.44
Subgroup: more than 21 days	14	147.876	<0.001	12.086	93.24
Subgroup: 21 days or less	11	66.308	<0.001	5.994	90.95
Sensitivity network	24	235.922	<0.001	8.622	92.37
AFC					
Main network	9	54.025	<0.001	8.025	92.60
Subgroup: mice	2	9.824	0.002	3.302	89.82
Subgroup: rats	7	44.201	<0.001	11.569	93.21
Subgroup: CTX	5	44.201	<0.001	11.569	93.21
Subgroup: non-CTX	4	9.824	0.002	3.302	89.82
Subgroup: more than 21 days	5	22.983	<0.001	14.119	91.30
Subgroup: 21 days or less	4	0.000	NA	NA	100.00
Sensitivity network	9	54.025	<0.001	8.025	92.60
E_2					
Main network	27	152.377	<0.001	2.758	85.56
Sensitivity network	26	152.363	<0.001	2.869	86.22
LH					
Main network	16	79.783	<0.001	3.171	86.21
Sensitivity network	15	79.327	<0.001	3.553	87.39
Primordial follicle					
Main network	10	17.972	0.003	0.817	72.18
Sensitivity network	9	17.540	0.002	0.914	77.19
Primary follicle					
Main network	11	59.452	<0.001	3.416	88.23
Sensitivity network	10	58.390	<0.001	3.662	89.72
Secondary follicle					
Main network	7	36.850	<0.001	2.844	86.43

Sensitivity network	6	35.634	<0.001	3.123	88.77
Atretic follicle					
Main network	14	129.124	<0.001	11.073	93.80
Sensitivity network	12	123.963	<0.001	12.701	95.16

FSH: follicle-stimulating hormone; AMH: anti-Müllerian hormone; AFC: antral follicle count; E_2: estradiol; LH: luteinizing hormone; CTX, cyclophosphamide; non-CTX, non-cyclophosphamide induction; Significant heterogeneity was defined as $P < 0.05$ or $I^2 > 50\%$.

Table S5 Pairwise heterogeneity for all outcome comparisons.

Outcome	Comparisons	Number of studies	Q statistic	P-value	τ^2	I^2 (%)
FSH	Resveratrol:Control	5	54.839	<0.001	14.588	92.71
FSH	Puerarin:Control	3	11.994	0.002	13.811	83.33
FSH	Quercetin:Control	7	39.931	<0.001	6.290	84.97
FSH	Quercetin:Genistein	1	0.000	NA	NA	NA
FSH	Genistein:Control	1	0.000	NA	NA	NA
FSH	Astragaloside IV:Control	3	0.588	0.745	0.000	0.00
FSH	Icariin:Control	5	75.394	<0.001	19.644	94.69
AMH	Resveratrol:Control	4	41.656	<0.001	3.440	92.80
AMH	Puerarin:Control	3	22.531	<0.001	38.303	91.12
AMH	Quercetin:Control	9	112.987	<0.001	17.598	92.92
AMH	Astragaloside IV:Control	3	5.241	0.073	1.054	61.84
AMH	Icariin:Control	5	55.544	<0.001	42.666	92.80
AMH	Genistein:Control	1	0.000	NA	NA	NA
AFC	Quercetin:Control	3	41.190	<0.001	57.729	95.14
AFC	Astragaloside IV:Control	2	3.012	0.083	1.674	66.80
AFC	Icariin:Control	2	9.824	0.002	3.302	89.82
AFC	Genistein:Control	1	0.000	NA	NA	NA
AFC	Resveratrol:Control	1	0.000	NA	NA	NA
E_2	Resveratrol:Control	6	50.926	<0.001	4.580	90.18
E_2	Puerarin:Control	3	2.421	0.298	0.159	17.40
E_2	Quercetin:Control	9	69.058	<0.001	9.208	88.42
E_2	Quercetin:Genistein	1	0.000	NA	NA	NA
E_2	Genistein:Control	3	8.026	0.018	0.452	75.08
E_2	Astragaloside IV:Control	3	10.997	0.004	2.494	81.81
E_2	Icariin:Control	4	7.952	0.047	1.744	62.28
LH	Resveratrol:Control	4	59.690	<0.001	10.175	94.97
LH	Puerarin:Control	2	0.038	0.846	0.000	0.00
LH	Quercetin:Control	5	13.886	0.008	0.762	71.19
LH	Quercetin:Genistein	1	0.000	NA	NA	NA
LH	Genistein:Control	1	0.000	NA	NA	NA
LH	Astragaloside IV:Control	2	0.712	0.399	0.000	0.00
LH	Icariin:Control	3	5.457	0.065	2.970	63.35
Primordial follicle	Icariin:Control	5	17.540	0.002	1.398	77.19
Primordial	Astragaloside	1	0.000	NA	NA	NA

follicle	IV:Control					
Primordial follicle	Quercetin:Control	2	0.432	0.511	0.000	0.00
Primordial follicle	Genistein:Control	1	0.000	NA	NA	NA
Primordial follicle	Resveratrol:Control	1	0.000	NA	NA	NA
Primary follicle	Resveratrol:Control	1	0.000	NA	NA	NA
Primary follicle	Quercetin:Control	4	21.923	<0.001	6.880	86.32
Primary follicle	Astragaloside IV:Control	2	25.504	<0.001	103.561	96.08
Primary follicle	Icariin:Control	4	12.024	0.007	1.028	75.05
Secondary follicle	Quercetin:Control	3	2.344	0.31	0.038	14.69
Secondary follicle	Icariin:Control	4	34.505	<0.001	27.622	91.31
Atretic follicle	Resveratrol:Control	3	58.442	<0.001	32.259	96.58
Atretic follicle	Puerarin:Control	2	0.460	0.497	0.000	0.00
Atretic follicle	Quercetin:Control	4	22.742	<0.001	40.507	86.81
Atretic follicle	Astragaloside IV:Control	1	0.000	NA	NA	NA
Atretic follicle	Icariin:Control	3	47.479	<0.001	20.497	95.79
Atretic follicle	Genistein:Control	1	0.000	NA	NA	NA
Subgroup Analysis						
FSH (mice)	Resveratrol:Control	2	19.960	<0.001	18.070	94.99
FSH (mice)	Puerarin:Control	1	0.000	NA	NA	NA
FSH (mice)	Quercetin:Control	2	2.065	0.151	1.038	51.58
FSH (mice)	Quercetin:Genistein	1	0.000	NA	NA	NA
FSH (mice)	Genistein:Control	1	0.000	NA	NA	NA
FSH (mice)	Icariin:Control	4	73.058	<0.001	26.496	95.89
FSH (rats)	Resveratrol:Control	3	33.955	<0.001	21.897	94.11
FSH (rats)	Puerarin:Control	2	0.002	0.967	0.000	0.00
FSH (rats)	Quercetin:Control	5	35.531	<0.001	9.704	88.74
FSH (rats)	Astragaloside IV:Control	3	0.588	0.745	0.000	0.00
FSH (rats)	Icariin:Control	1	0.000	NA	NA	NA
FSH (CTX)	Puerarin:Control	2	0.002	0.967	0.000	0.00
FSH (CTX)	Quercetin:Control	5	29.967	<0.001	7.231	86.65
FSH (CTX)	Astragaloside IV:Control	3	0.588	0.745	0.000	0.00

FSH (CTX)	Icariin:Control	2	12.400	<0.001	4.509	91.94
FSH (CTX)	Resveratrol:Control	2	21.301	<0.001	47.591	95.31
FSH (non-CTX)	Resveratrol:Control	3	28.622	<0.001	8.323	93.01
FSH (non-CTX)	Puerarin:Control	1	0.000	NA	NA	NA
FSH (non-CTX)	Quercetin:Control	2	6.552	0.01	8.105	84.74
FSH (non-CTX)	Quercetin:Genistein	1	0.000	NA	NA	NA
FSH (non-CTX)	Genistein:Control	1	0.000	NA	NA	NA
FSH (non-CTX)	Icariin:Control	3	33.192	<0.001	20.070	93.97
FSH (more than 21 days)	Resveratrol:Control	3	28.622	<0.001	8.323	93.01
FSH (more than 21 days)	Quercetin:Control	5	27.920	<0.001	7.344	85.67
FSH (more than 21 days)	Quercetin:Genistein	1	0.000	NA	NA	NA
FSH (more than 21 days)	Genistein:Control	1	0.000	NA	NA	NA
FSH (more than 21 days)	Astragaloside IV:Control	3	0.588	0.745	0.000	0.00
FSH (more than 21 days)	Icariin:Control	4	61.642	<0.001	13.675	95.13
FSH (21 days or less)	Puerarin:Control	3	11.994	0.002	13.811	83.33
FSH (21 days or less)	Quercetin:Control	2	8.274	0.004	6.570	87.91
FSH (21 days or less)	Resveratrol:Control	2	21.301	<0.001	47.591	95.31
FSH (21 days or less)	Icariin:Control	1	0.000	NA	NA	NA
AMH (mice)	Resveratrol:Control	1	0.000	NA	NA	NA
AMH (mice)	Puerarin:Control	1	0.000	NA	NA	NA
AMH (mice)	Quercetin:Control	2	13.332	<0.001	10.487	92.50
AMH (mice)	Icariin:Control	3	14.284	<0.001	52.195	86.00
AMH (rats)	Resveratrol:Control	3	9.901	0.007	1.773	79.80
AMH (rats)	Puerarin:Control	2	2.144	0.143	1.286	53.35
AMH (rats)	Quercetin:Control	7	98.846	<0.001	22.261	93.93
AMH (rats)	Astragaloside IV:Control	3	5.241	0.073	1.054	61.84
AMH (rats)	Icariin:Control	2	10.940	<0.001	16.477	90.86

AMH (rats)	Genistein:Control	1	0.000	NA	NA	NA
AMH (CTX)	Puerarin:Control	2	2.144	0.143	1.286	53.35
AMH (CTX)	Quercetin:Control	7	97.946	<0.001	22.129	93.87
AMH (CTX)	Astragaloside IV:Control	3	5.241	0.073	1.054	61.84
AMH (CTX)	Icariin:Control	1	0.000	NA	NA	NA
AMH (CTX)	Resveratrol:Control	1	0.000	NA	NA	NA
AMH (non-CTX)	Resveratrol:Control	3	41.604	<0.001	5.030	95.19
AMH (non-CTX)	Puerarin:Control	1	0.000	NA	NA	NA
AMH (non-CTX)	Icariin:Control	4	52.160	<0.001	61.877	94.25
AMH (non-CTX)	Quercetin:Control	2	14.226	<0.001	11.864	92.97
AMH (non-CTX)	Genistein:Control	1	0.000	NA	NA	NA
AMH (more than 21 days)	Resveratrol:Control	2	36.299	<0.001	10.007	97.25
AMH (more than 21 days)	Quercetin:Control	5	51.312	<0.001	24.462	92.20
AMH (more than 21 days)	Astragaloside IV:Control	3	5.241	0.073	1.054	61.84
AMH (more than 21 days)	Icariin:Control	4	55.024	<0.001	58.830	94.55
AMH (21 days or less)	Puerarin:Control	3	22.531	<0.001	38.303	91.12
AMH (21 days or less)	Quercetin:Control	4	42.220	<0.001	7.876	92.89
AMH (21 days or less)	Icariin:Control	1	0.000	NA	NA	NA
AMH (21 days or less)	Resveratrol:Control	2	1.557	0.212	0.252	35.76
AMH (21 days or less)	Genistein:Control	1	0.000	NA	NA	NA
AFC (mice)	Icariin:Control	2	9.824	0.002	3.302	89.82
AFC (rats)	Quercetin:Control	3	41.190	<0.001	57.729	95.14
AFC (rats)	Astragaloside IV:Control	2	3.012	0.083	1.674	66.80
AFC (rats)	Genistein:Control	1	0.000	NA	NA	NA
AFC (rats)	Resveratrol:Control	1	0.000	NA	NA	NA
AFC (CTX)	Quercetin:Control	3	41.190	<0.001	57.729	95.14
AFC (CTX)	Astragaloside IV:Control	2	3.012	0.083	1.674	66.80

AFC (non-CTX)	Icariin:Control	2	9.824	0.002	3.302	89.82
AFC (non-CTX)	Genistein:Control	1	0.000	NA	NA	NA
AFC (non-CTX)	Resveratrol:Control	1	0.000	NA	NA	NA
AFC (more than 21 days)	Quercetin:Control	2	19.972	<0.001	66.787	94.99
AFC (more than 21 days)	Astragaloside IV:Control	2	3.012	0.083	1.674	66.80
AFC (more than 21 days)	Icariin:Control	1	0.000	NA	NA	NA
AFC (21 days or less)	Icariin:Control	1	0.000	NA	NA	NA
AFC (21 days or less)	Genistein:Control	1	0.000	NA	NA	NA
AFC (21 days or less)	Resveratrol:Control	1	0.000	NA	NA	NA
AFC (21 days or less)	Quercetin:Control	1	0.000	NA	NA	NA

NA, not applicable (heterogeneity could not be estimated because only one study contributed to this comparison). Significant heterogeneity was defined as $P < 0.05$ or $I^2 > 50\%$.

Table S6 SUCRA values of different interventions across various outcomes.

Intervention	FSH	AMH	AFC	E_2	LH	Primordial follicle	Primary follicle	Secondary follicle	Atretic follicle
Quercetin	0.7173	0.6065	0.8194	0.6337	0.3993	0.7194	0.7228	0.9360	0.9420
Icariin	0.5597	0.9855	0.2818	0.9210	0.9972	0.2654	0.4412	0.5635	0.6425
Astragaloside IV	0.2533	0.4972	0.7946	0.6648	0.4848	1.0000	0.9770	0.0000	0.1822
Resveratrol	0.4310	0.3152	0.4606	0.4117	0.7130	0.5074	0.2785	0.0000	0.5355
Puerarin	0.8015	0.7752	0.0000	0.6058	0.7022	0.0000	0.0000	0.0000	0.4897
Genistein	0.7327	0.2672	0.4882	0.2602	0.0960	0.5064	0.0000	0.0000	0.5695
Control	0.0045	0.0533	0.1554	0.0028	0.1075	0.0014	0.0805	0.0005	0.1387

A higher surface under the cumulative ranking curve (SUCRA) value indicates a greater probability of the intervention being the best treatment for that specific endpoint, whereas zero values denote a lack of direct or indirect evidence for the intervention in the corresponding outcome.

Table S7 Summary of overall efficacy rankings and comparison between best-dose and all-dose network meta-analyses for different interventions.

Intervention	Best-dose network meta-analysis					All-dose network meta-analysis				
	Total SUCRA	Global mean SUCRA	Available mean SUCRA	Overall efficacy ranking	Available-data efficacy ranking	Total SUCRA	Global mean SUCRA	Available mean SUCRA	Overall efficacy ranking	Available-data efficacy ranking
Quercetin	6.4964	0.7218	0.7218	1	1	5.0584	0.5620	0.5620	2	3
Icariin	5.6578	0.6286	0.6286	2	3	4.5078	0.5009	0.5009	3	6
Astragaloside IV	4.8539	0.5393	0.6067	3	4	5.2748	0.5861	0.6594	1	2
Resveratrol	3.6529	0.4059	0.4566	4	5	4.2119	0.4680	0.5265	5	4
Puerarin	3.3744	0.3749	0.6749	5	2	4.4669	0.4963	0.8934	4	1
Genistein	2.9202	0.3245	0.4172	6	6	3.6530	0.4059	0.5219	6	5
Control	0.5446	0.0605	0.0605	7	7	0.3272	0.0364	0.0409	7	7

The overall efficacy ranking is sorted in descending order based on the global mean SUCRA, which is calculated as the total SUCRA divided by the total number of predefined endpoints. Missing SUCRA values for individual endpoints were assigned a value of zero. Results based on available-case data are provided as a sensitivity analysis to evaluate the impact of assigning zero to missing SUCRA values on the rankings.

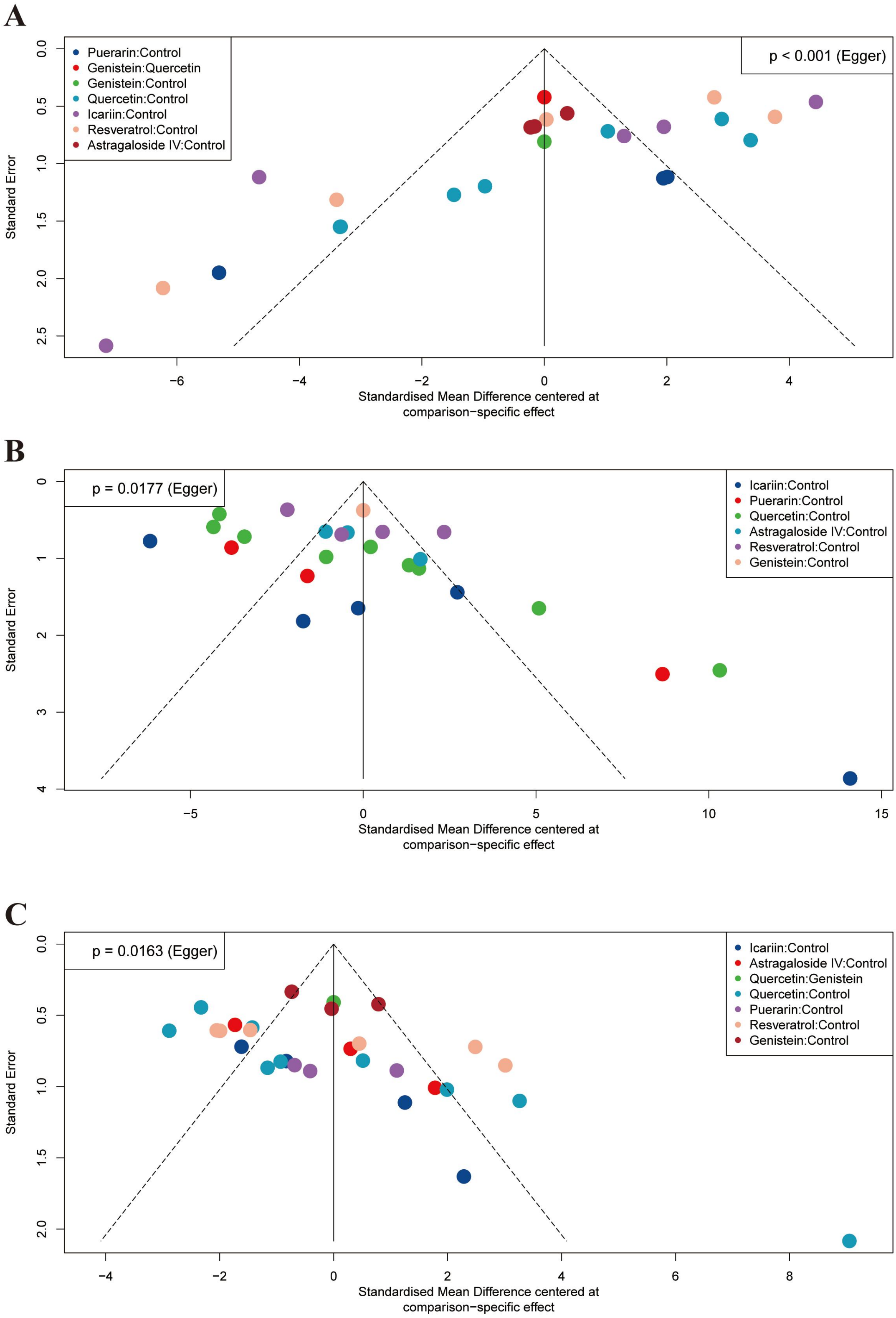

Figure S1 Comparison-adjusted funnel plots of ovarian function outcomes, including (A) FSH, (B) AMH, and (C) E_2. Egger's test was used to assess publication bias, with $p < 0.1$ indicating significant asymmetry.

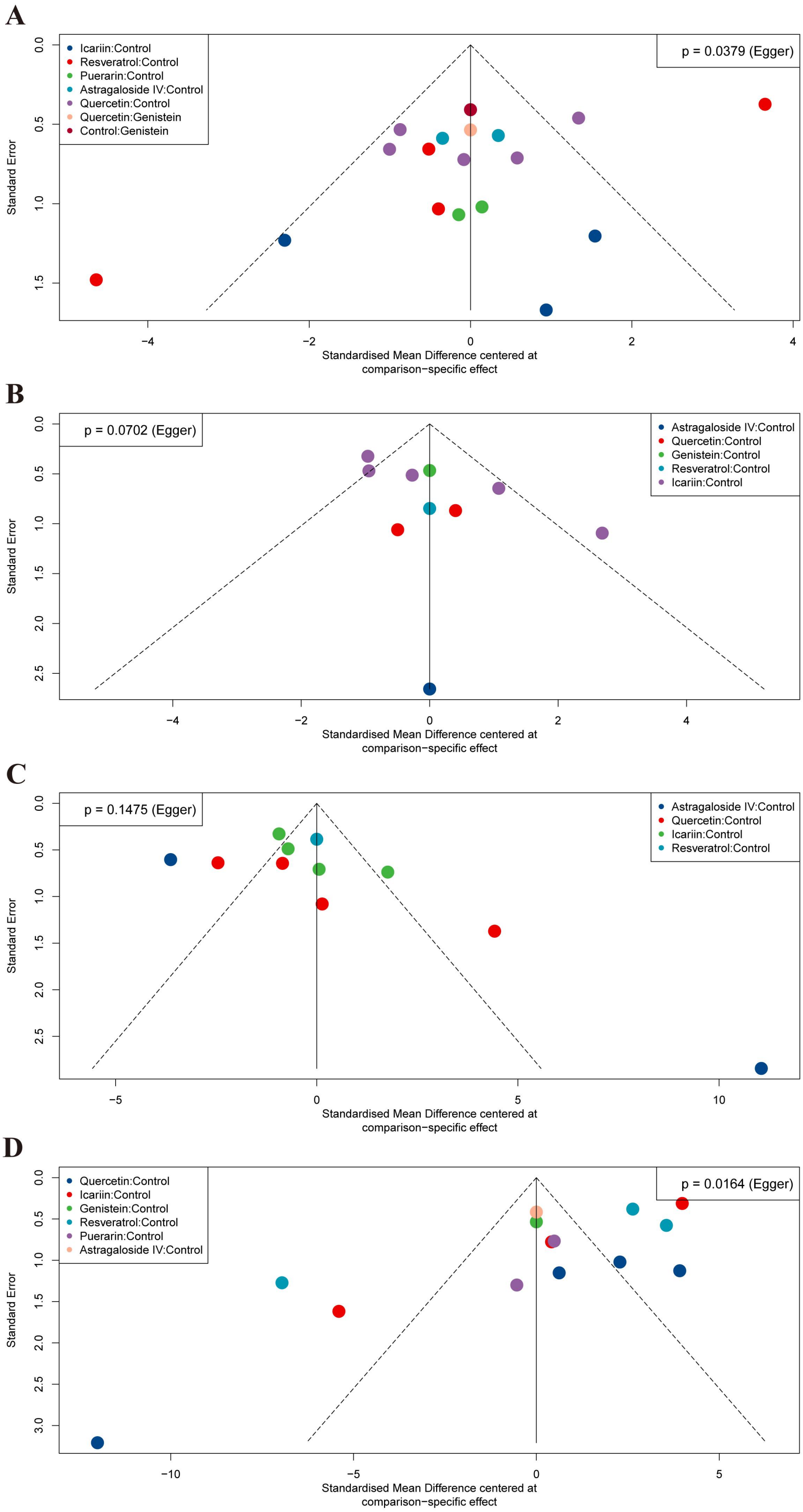

Figure S2 Comparison-adjusted funnel plots of ovarian function outcomes, including (A) LH, (B) primordial follicle, (C) primary follicle and (D) atretic follicle. Egger's test was used to assess publication bias, with $p < 0.1$ indicating significant asymmetry.

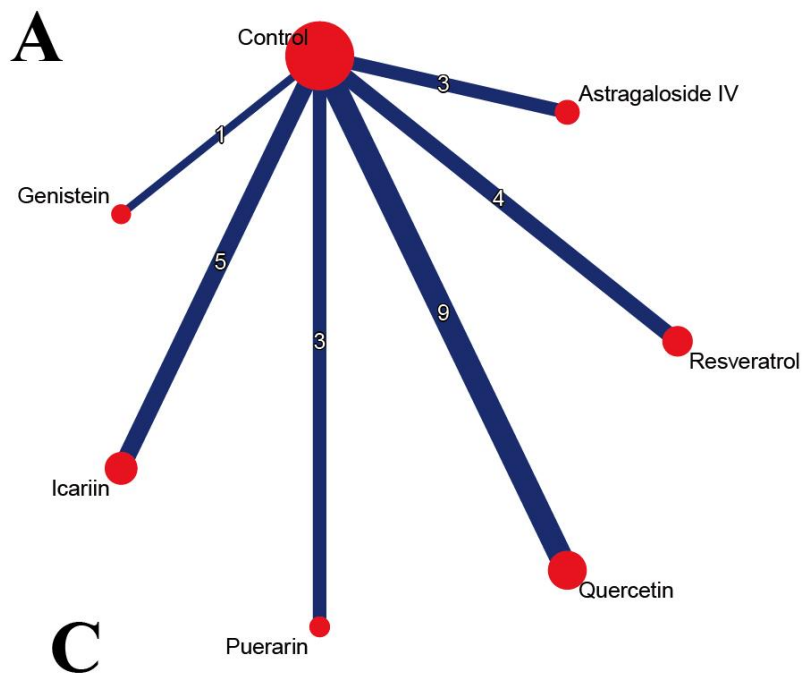

B

Astragaloside IV						
2.28 (-4.22,8.78)	Genistein					
**-6.10 (-10.54,-1.65)**	**-8.38 (-14.70,-2.06)**	Icariin				
-2.85 (-7.78,2.07)	-5.13 (-11.80,1.54)	3.24 (-1.44,7.93)	Puerarin			
-0.88 (-4.75,3.00)	-3.16 (-9.09,2.78)	**5.22 (1.66,8.78)**	1.98 (-2.17,6.13)	Quercetin		
1.61 (-2.76,5.97)	-0.67 (-6.94,5.59)	**7.71 (3.61,11.80)**	4.46 (-0.15,9.08)	2.49 (-0.98,5.95)	Resveratrol	
**4.08 (0.76,7.40)**	1.80 (-3.79,7.39)	**10.18 (7.22,13.13)**	**6.93 (3.29,10.57)**	**4.96 (2.96,6.95)**	2.47 (-0.36,5.30)	Control

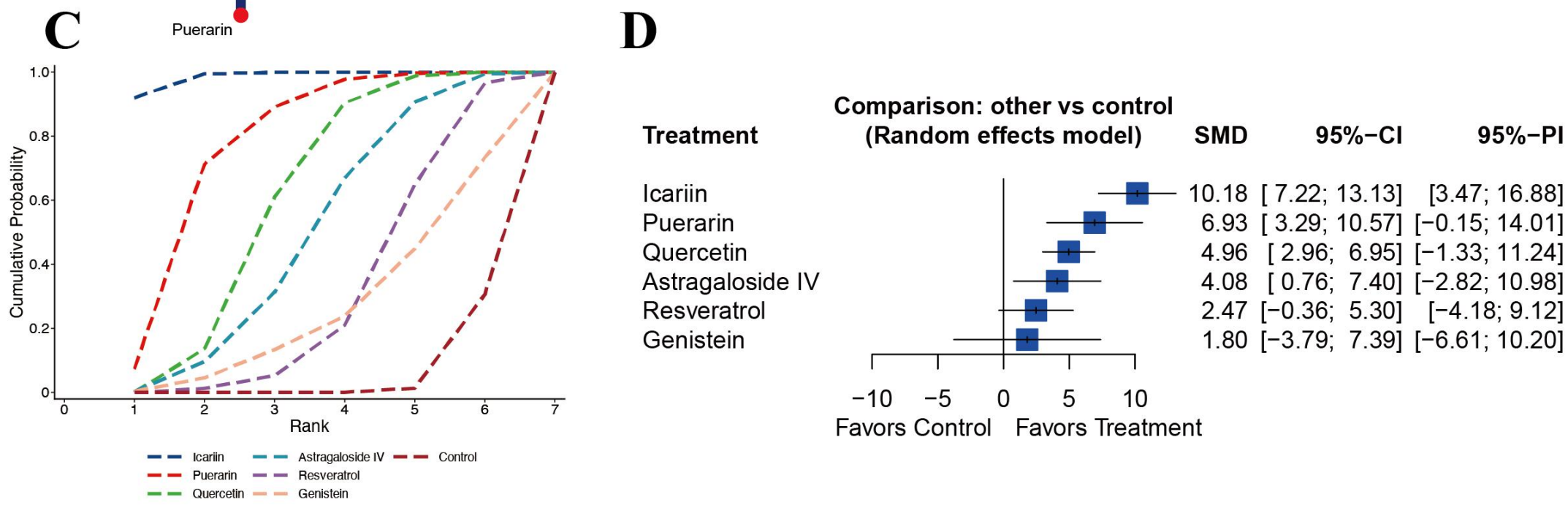

Figure S3 Network meta-analysis of AMH levels. (A) Network plot of eligible comparisons, where node sizes are proportional to the total animal sample sizes, and line widths correspond to the number of direct comparisons. The numbers on the lines represent the number of trials. (B) League table summarizing all pairwise comparisons. Data are expressed as standardized mean differences (SMDs) with 95% confidence intervals (CIs). Bold values indicate statistical significance. (C) SUCRA plots showing cumulative ranking probabilities. A larger area under the curve indicates a higher probability of the intervention being top-ranked. (D) Forest plot comparing the effects of TCM monomers with the control group based on a random-effects model.

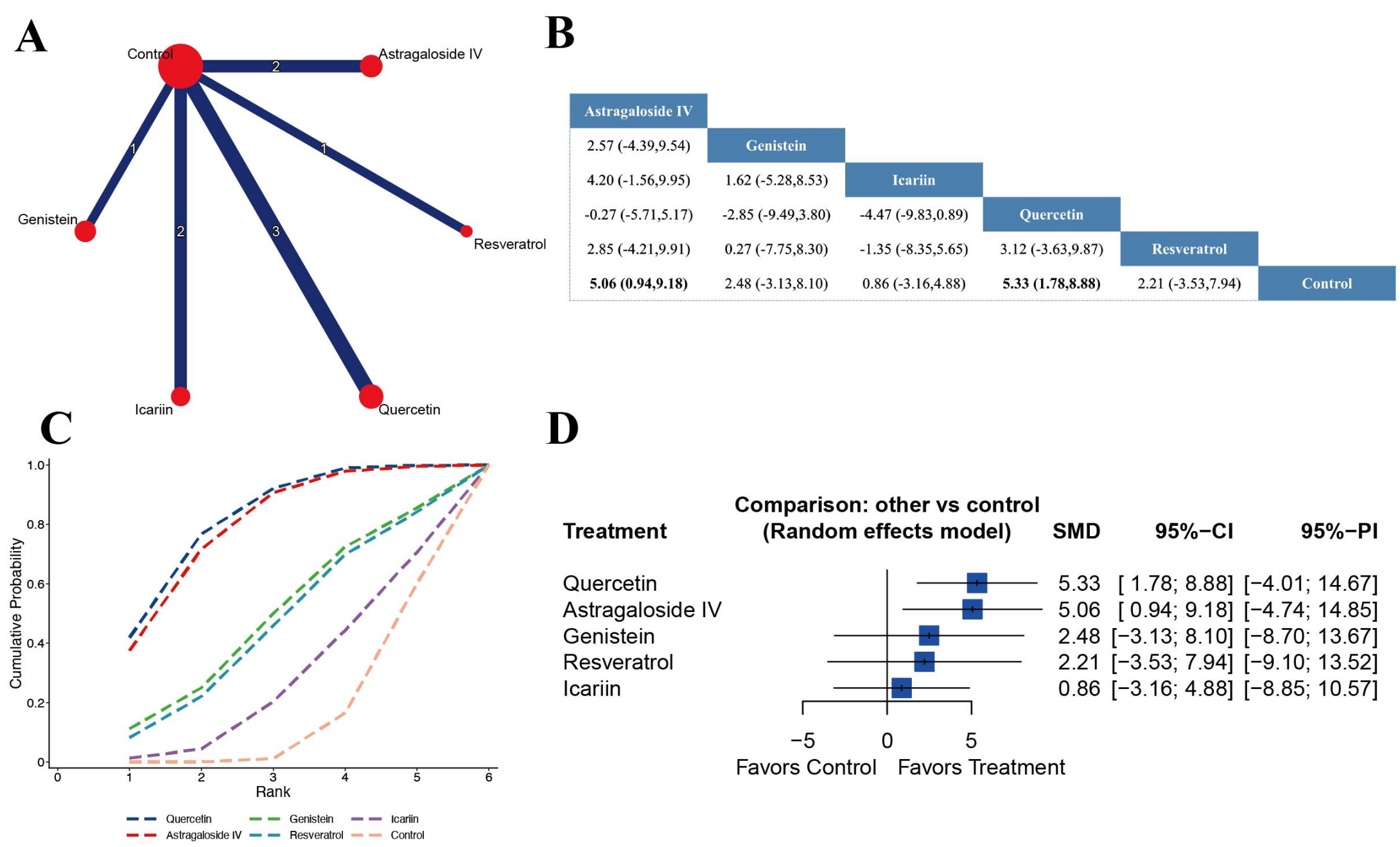

Figure S4 Network meta-analysis of AFC levels. (A) Network plot of eligible comparisons, where node sizes are proportional to the total animal sample sizes, and line widths correspond to the number of direct comparisons. The numbers on the lines represent the number of trials. (B) League table summarizing all pairwise comparisons. Data are expressed as standardized mean differences (SMDs) with 95% confidence intervals (CIs). Bold values indicate statistical significance. (C) SUCRA plots showing cumulative ranking probabilities. A larger area under the curve indicates a higher probability of the intervention being top-ranked. (D) Forest plot comparing the effects of TCM monomers with the control group based on a random-effects model.

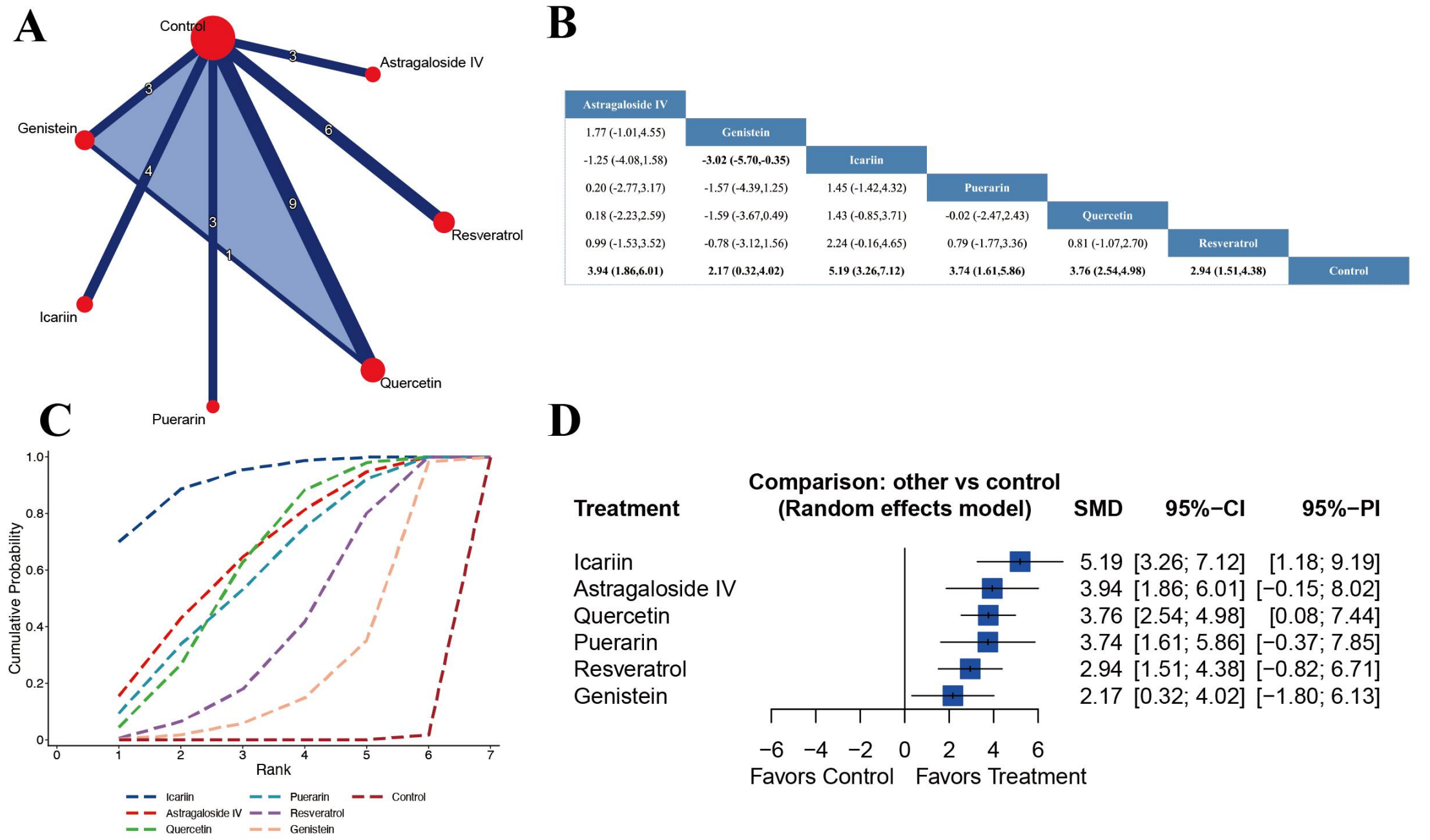

**Astragaloside IV**						
1.77 (-1.01,4.55)	**Genistein**					
-1.25 (-4.08,1.58)	**-3.02 (-5.70,-0.35)**	**Icariin**				
0.20 (-2.77,3.17)	-1.57 (-4.39,1.25)	1.45 (-1.42,4.32)	**Puerarin**			
0.18 (-2.23,2.59)	-1.59 (-3.67,0.49)	1.43 (-0.85,3.71)	-0.02 (-2.47,2.43)	**Quercetin**		
0.99 (-1.53,3.52)	-0.78 (-3.12,1.56)	2.24 (-0.16,4.65)	0.79 (-1.77,3.36)	0.81 (-1.07,2.70)	**Resveratrol**	
**3.94 (1.86,6.01)**	**2.17 (0.32,4.02)**	**5.19 (3.26,7.12)**	**3.74 (1.61,5.86)**	**3.76 (2.54,4.98)**	**2.94 (1.51,4.38)**	**Control**

Figure S5 Network meta-analysis of E_2 levels. (A) Network plot of eligible comparisons, where node sizes are proportional to the total animal sample sizes, and line widths correspond to the number of direct comparisons. The numbers on the lines represent the number of trials. The shaded area denotes a multi-arm trial. (B) League table summarizing all pairwise comparisons. Data are expressed as standardized mean differences (SMDs) with 95% confidence intervals (CIs). Bold values indicate statistical significance. (C) SUCRA plots showing cumulative ranking probabilities. A larger area under the curve indicates a higher probability of the intervention being top-ranked. (D) Forest plot comparing the effects of TCM monomers with the control group based on a random-effects model.

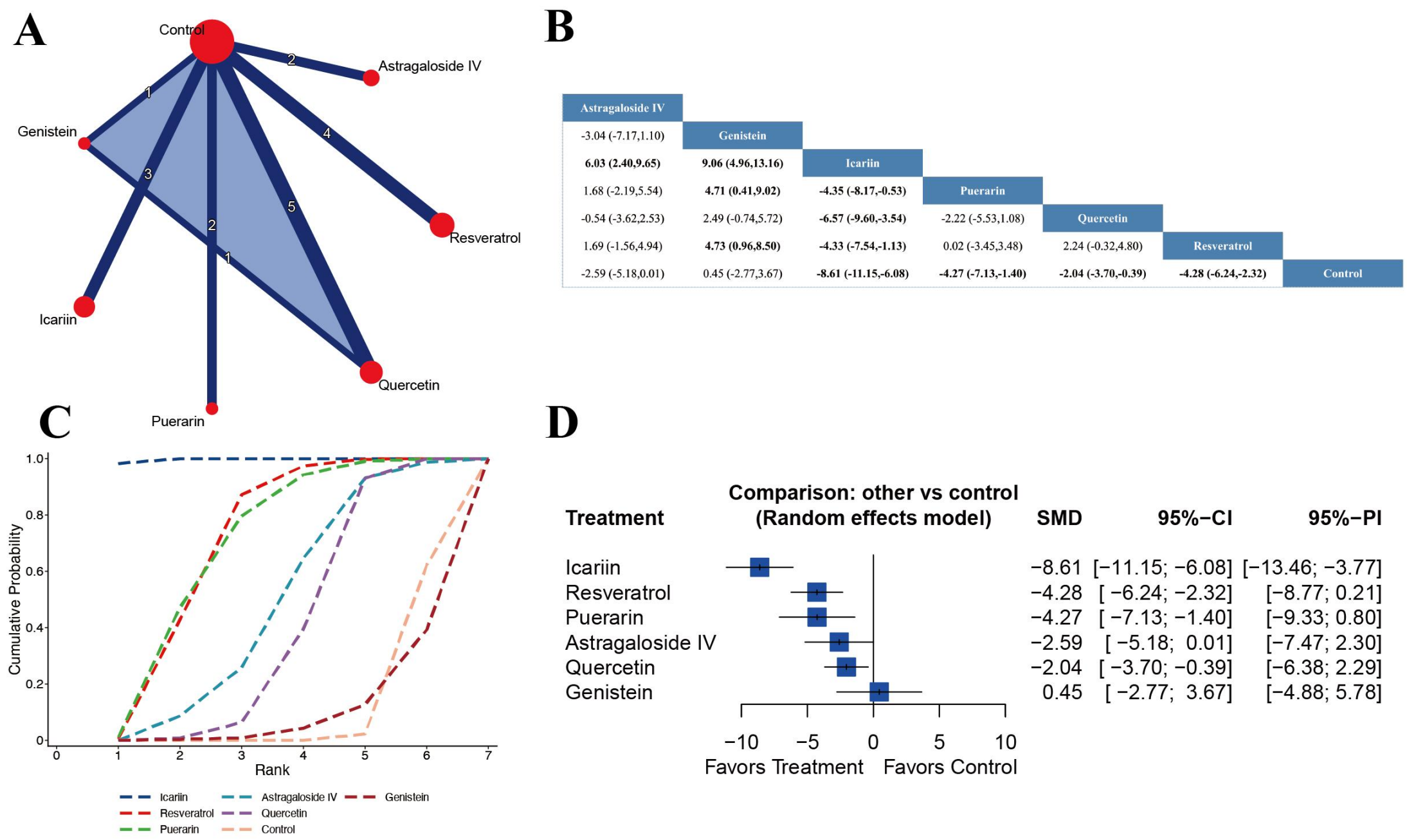

Figure S6 Network meta-analysis of LH levels. (A) Network plot of eligible comparisons, where node sizes are proportional to the total animal sample sizes, and line widths correspond to the number of direct comparisons. The numbers on the lines represent the number of trials. The shaded area denotes a multi-arm trial. (B) League table summarizing all pairwise comparisons. Data are expressed as standardized mean differences (SMDs) with 95% confidence intervals (CIs). Bold values indicate statistical significance. (C) SUCRA plots showing cumulative ranking probabilities. A larger area under the curve indicates a higher probability of the intervention being top-ranked. (D) Forest plot comparing the effects of TCM monomers with the control group based on a random-effects model.

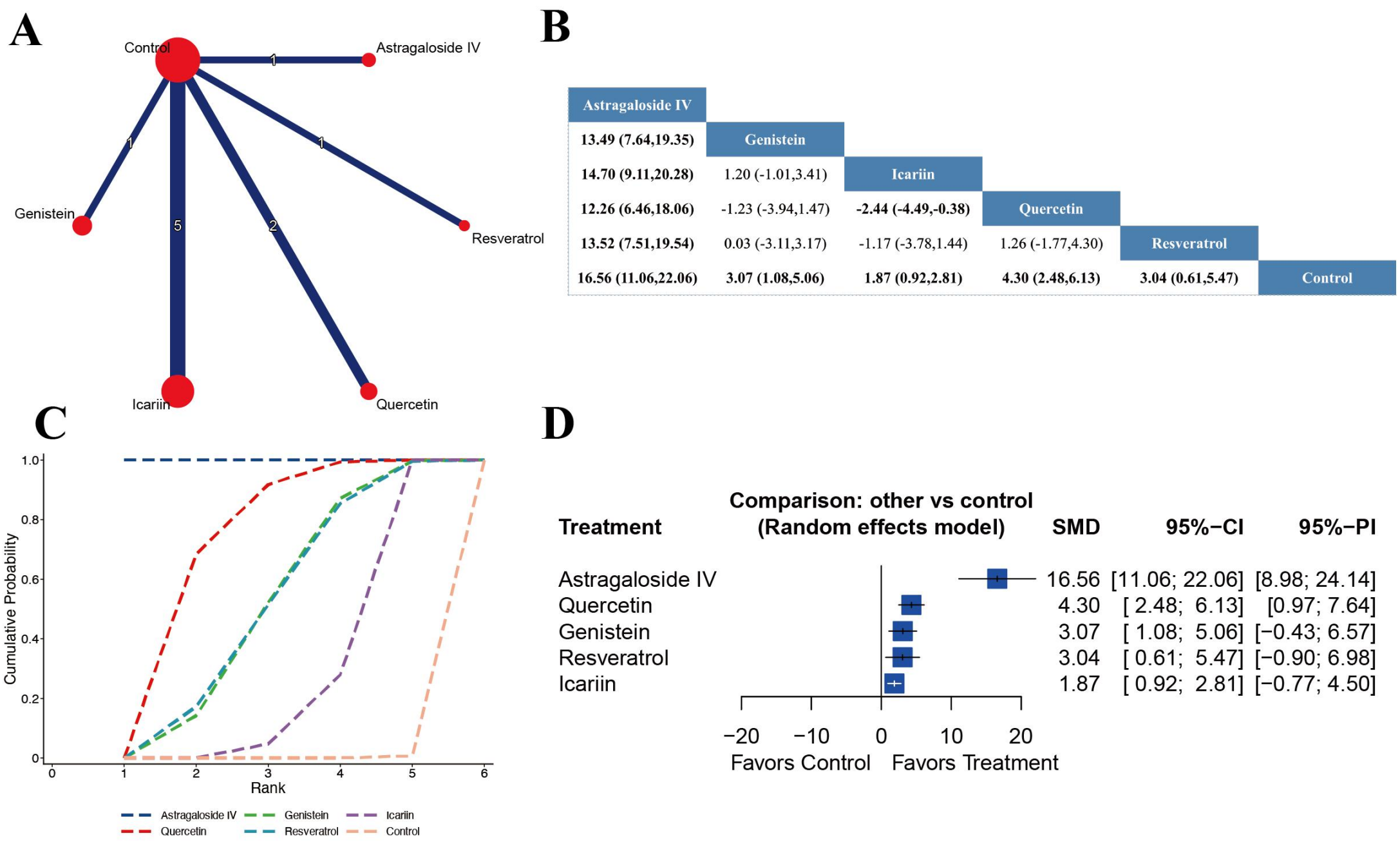

Figure S7 Network meta-analysis of primordial follicles. (A) Network plot of eligible comparisons, where node sizes are proportional to the total animal sample sizes, and line widths correspond to the number of direct comparisons. The numbers on the lines represent the number of trials. (B) League table summarizing all pairwise comparisons. Data are expressed as standardized mean differences (SMDs) with 95% confidence intervals (CIs). Bold values indicate statistical significance. (C) SUCRA plots showing cumulative ranking probabilities. A larger area under the curve indicates a higher probability of the intervention being top-ranked. (D) Forest plot comparing the effects of TCM monomers with the control group based on a random-effects model.

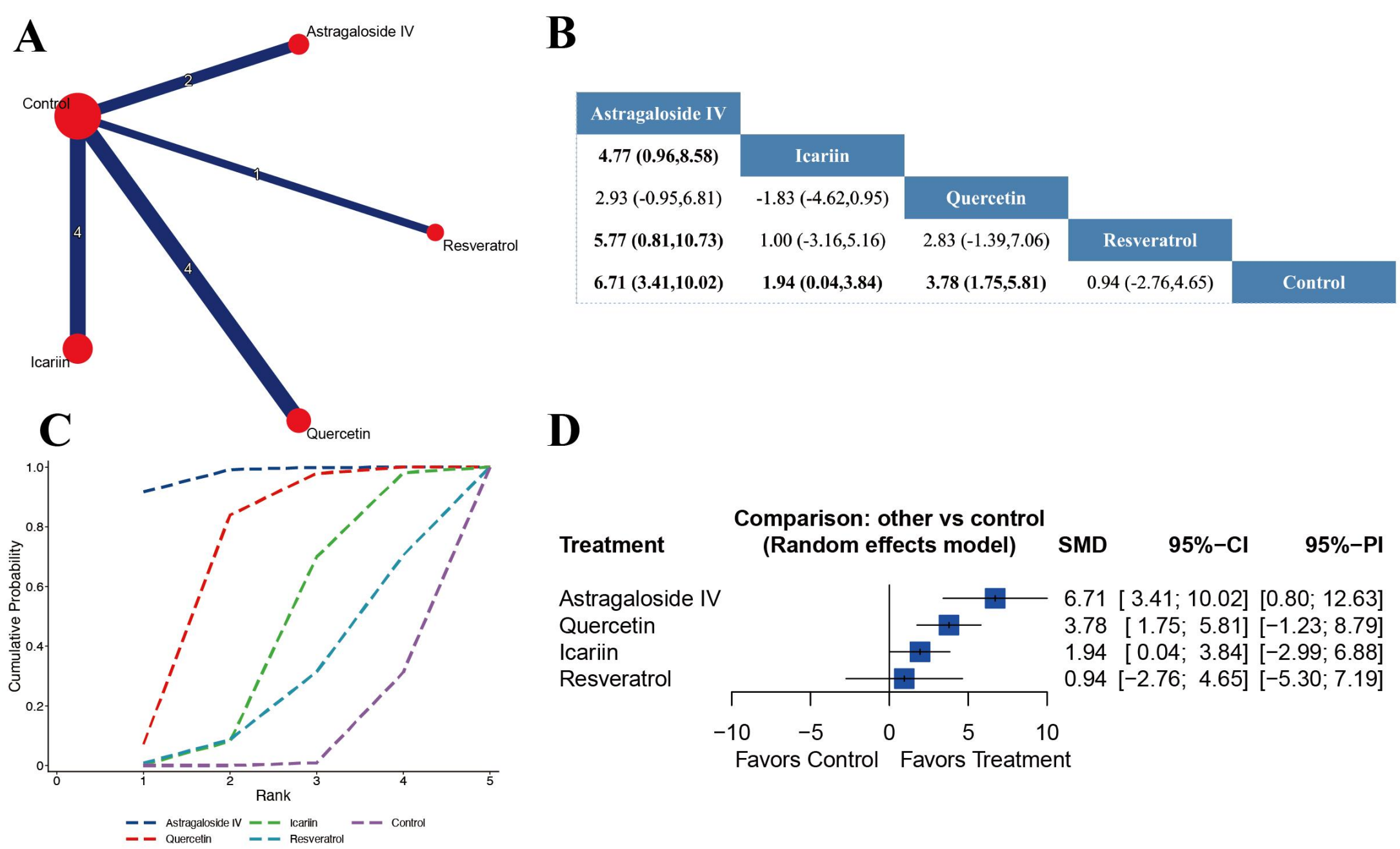

Figure S8 Network meta-analysis of primary follicles. (A) Network plot of eligible comparisons, where node sizes are proportional to the total animal sample sizes, and line widths correspond to the number of direct comparisons. The numbers on the lines represent the number of trials. (B) League table summarizing all pairwise comparisons. Data are expressed as standardized mean differences (SMDs) with 95% confidence intervals (CIs). Bold values indicate statistical significance. (C) SUCRA plots showing cumulative ranking probabilities. A larger area under the curve indicates a higher probability of the intervention being top-ranked. (D) Forest plot comparing the effects of TCM monomers with the control group based on a random-effects model.

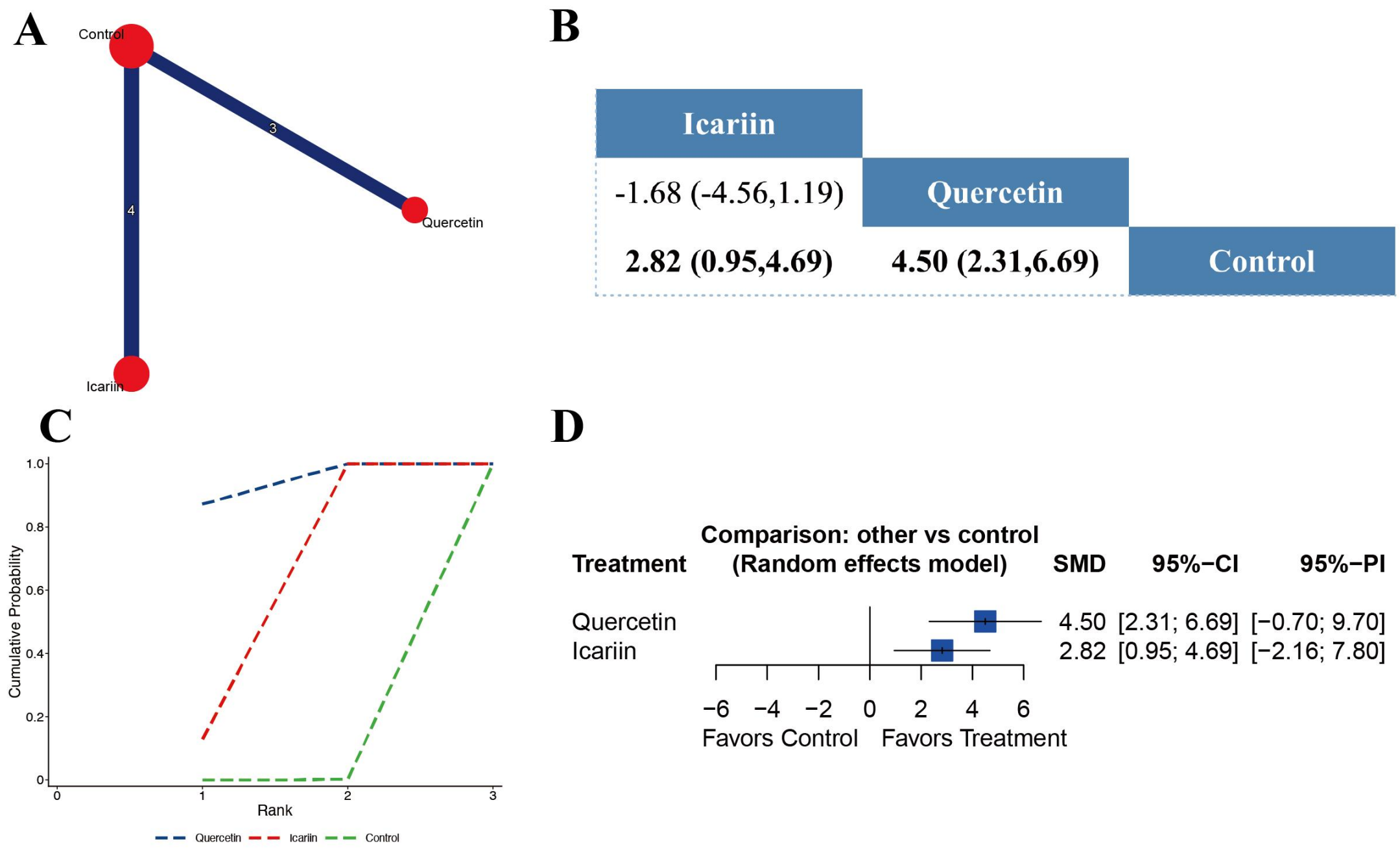

Figure S9 Network meta-analysis of secondary follicles. (A) Network plot of eligible comparisons, where node sizes are proportional to the total animal sample sizes, and line widths correspond to the number of direct comparisons. The numbers on the lines represent the number of trials. (B) League table summarizing all pairwise comparisons. Data are expressed as standardized mean differences (SMDs) with 95% confidence intervals (CIs). Bold values indicate statistical significance. (C) SUCRA plots showing cumulative ranking probabilities. A larger area under the curve indicates a higher probability of the intervention being top-ranked. (D) Forest plot comparing the effects of TCM monomers with the control group based on a random-effects model.

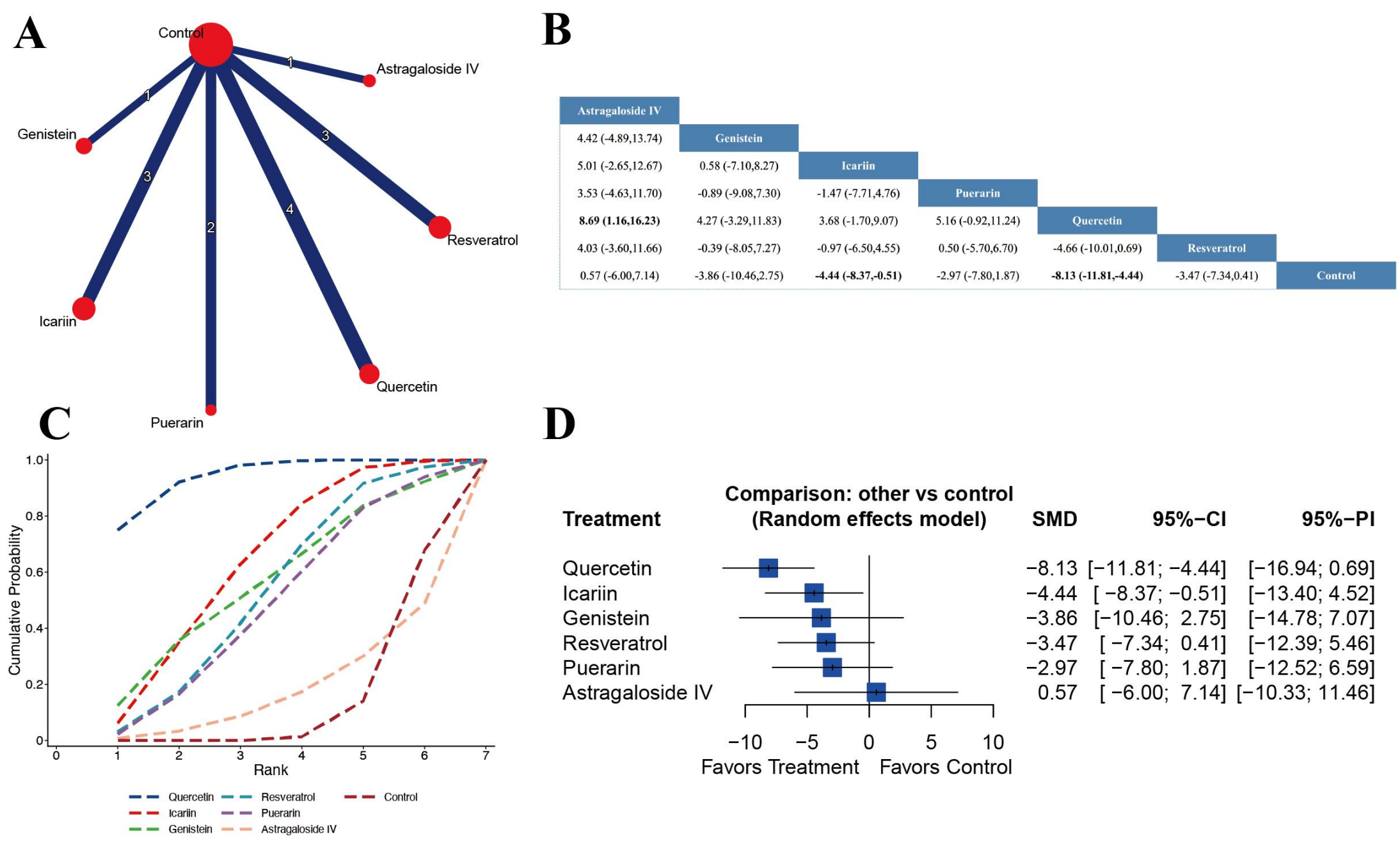

Figure S10 Network meta-analysis of atretic follicles. (A) Network plot of eligible comparisons, where node sizes are proportional to the total animal sample sizes, and line widths correspond to the number of direct comparisons. The numbers on the lines represent the number of trials. (B) League table summarizing all pairwise comparisons. Data are expressed as standardized mean differences (SMDs) with 95% confidence intervals (CIs). Bold values indicate statistical significance. (C) SUCRA plots showing cumulative ranking probabilities. A larger area under the curve indicates a higher probability of the intervention being top-ranked. (D) Forest plot comparing the effects of TCM monomers with the control group based on a random-effects model.

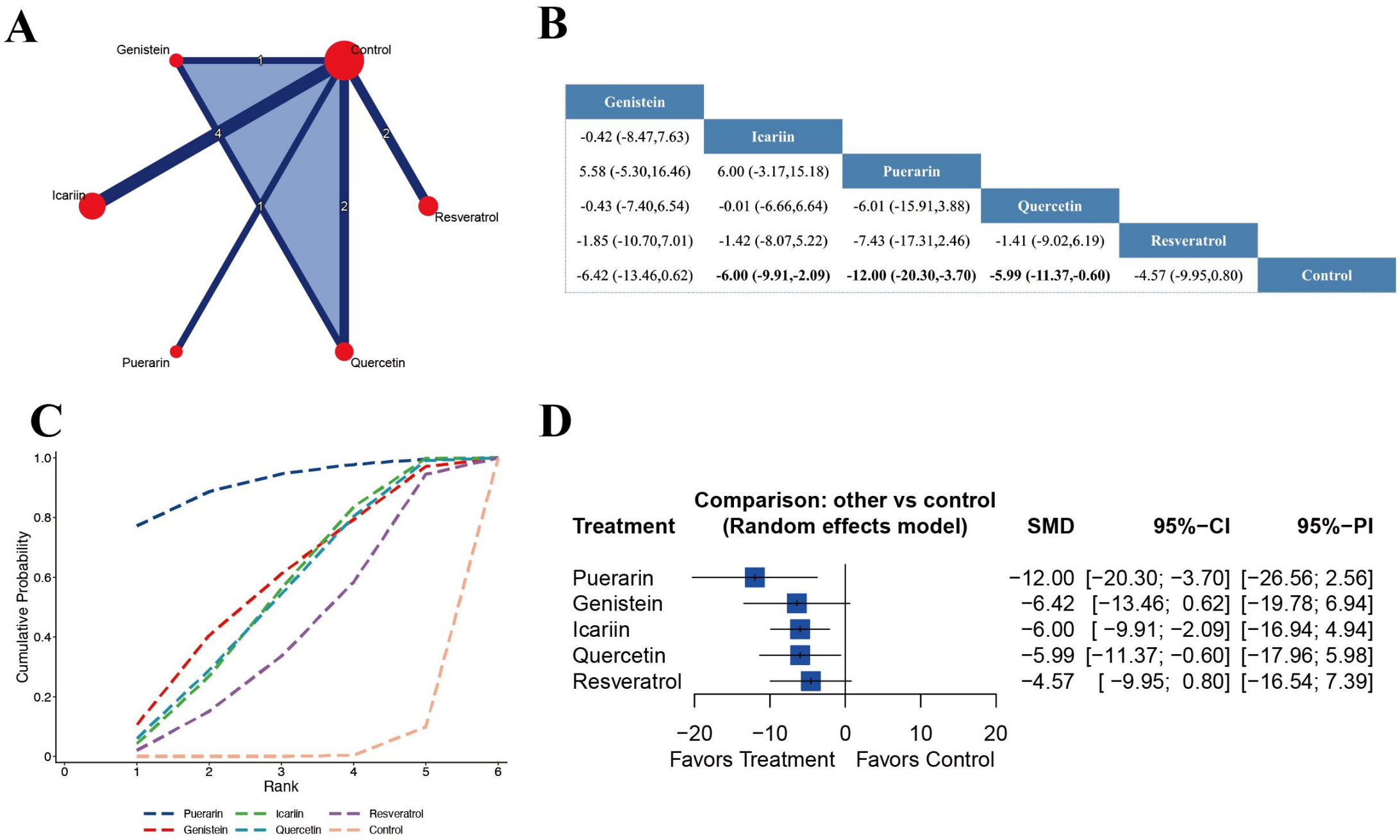

Figure S11 Subgroup analysis: network meta-analysis of FSH levels in mice. (A) Network plot of eligible comparisons. (B) League table summarizing all pairwise comparisons. Bold values represent statistical significance. (C) SUCRA plots showing cumulative ranking probabilities. A larger area under the curve indicates a higher probability of the intervention being top-ranked. (D) Forest plot comparing the effects of TCM monomers with the control group based on a random-effects model.

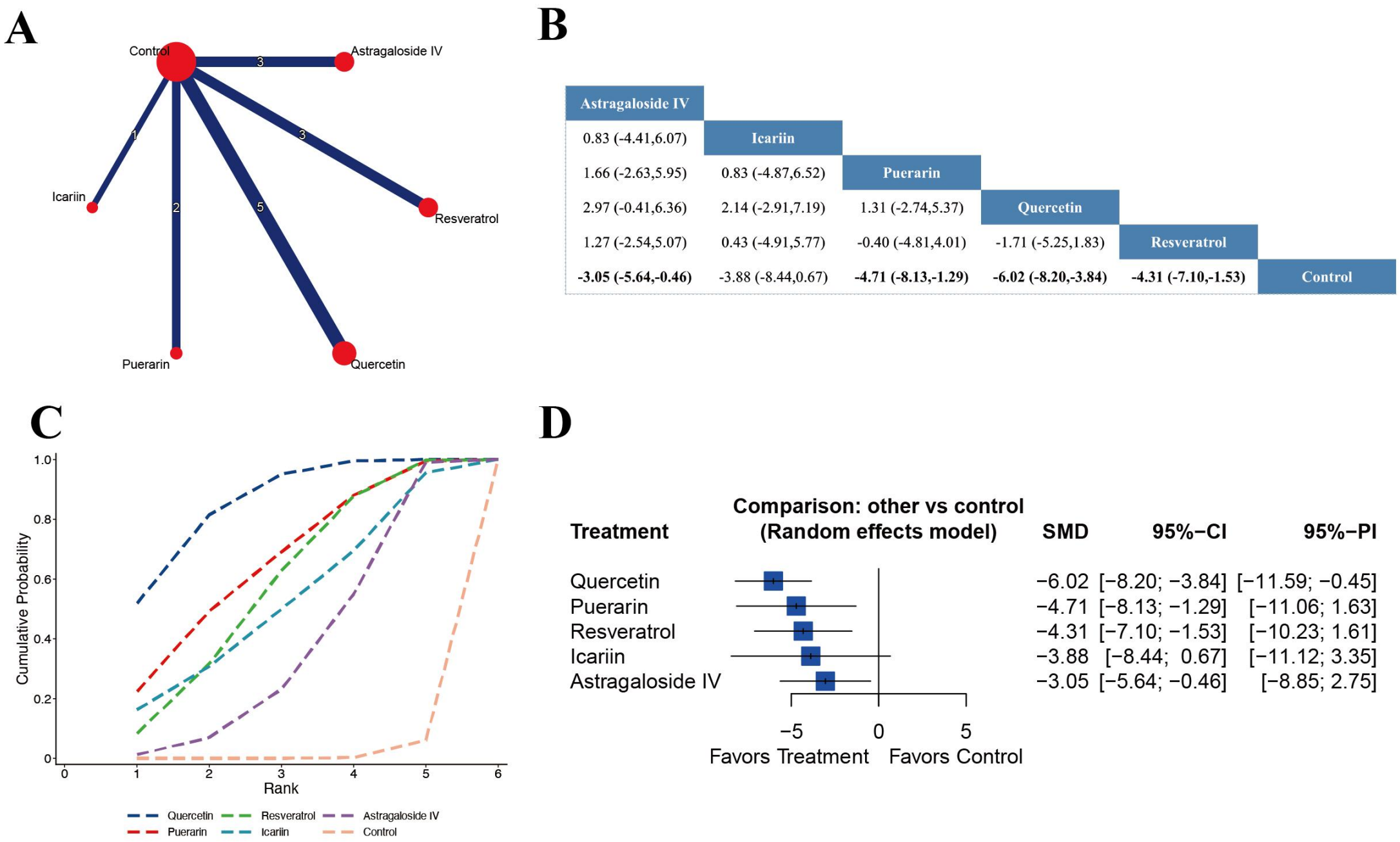

Figure S12 Subgroup analysis: network meta-analysis of FSH levels in rats. (A) Network plot of eligible comparisons. (B) League table summarizing all pairwise comparisons. Bold values represent statistical significance. (C) SUCRA plots showing cumulative ranking probabilities. A larger area under the curve indicates a higher probability of the intervention being top-ranked. (D) Forest plot comparing the effects of TCM monomers with the control group based on a random-effects model.

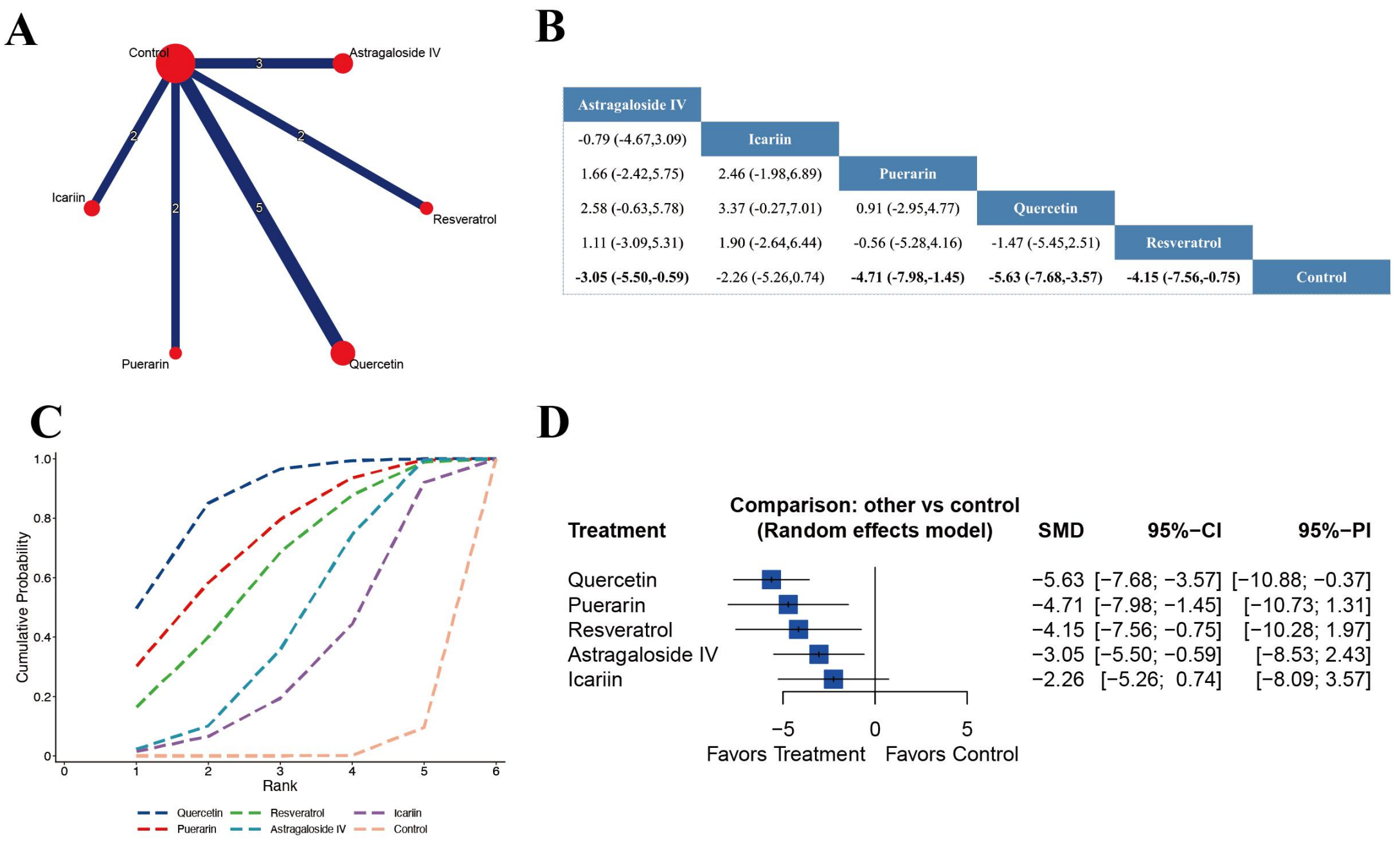

Astragaloside IV					
-0.79 (-4.67,3.09)	Icariin				
1.66 (-2.42,5.75)	2.46 (-1.98,6.89)	Puerarin			
2.58 (-0.63,5.78)	3.37 (-0.27,7.01)	0.91 (-2.95,4.77)	Quercetin		
1.11 (-3.09,5.31)	1.90 (-2.64,6.44)	-0.56 (-5.28,4.16)	-1.47 (-5.45,2.51)	Resveratrol	
**-3.05 (-5.50,-0.59)**	-2.26 (-5.26,0.74)	**-4.71 (-7.98,-1.45)**	**-5.63 (-7.68,-3.57)**	**-4.15 (-7.56,-0.75)**	Control

Figure S13 Subgroup analysis: network meta-analysis of FSH levels in cyclophosphamide (CTX) induced animal models. (A) Network plot of eligible comparisons. (B) League table summarizing all pairwise comparisons. Bold values represent statistical significance. (C) SUCRA plots showing cumulative ranking probabilities. A larger area under the curve indicates a higher probability of the intervention being top-ranked. (D) Forest plot comparing the effects of TCM monomers with the control group based on a random-effects model.

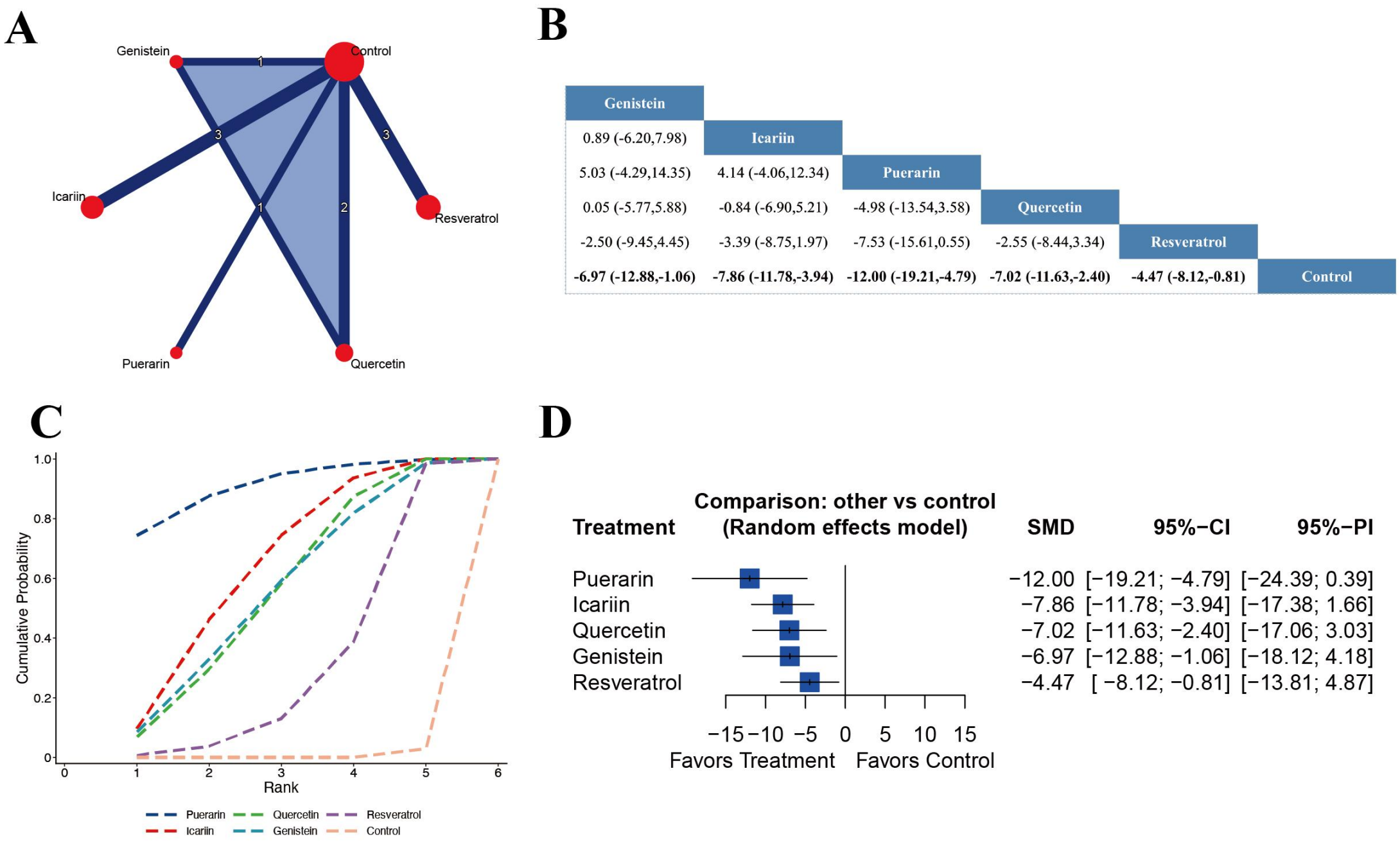

Figure S14 Subgroup analysis: network meta-analysis of FSH levels in non-CTX induced animal models. (A) Network plot of eligible comparisons. (B) League table summarizing all pairwise comparisons. Bold values represent statistical significance. (C) SUCRA plots showing cumulative ranking probabilities. A larger area under the curve indicates a higher probability of the intervention being top-ranked. (D) Forest plot comparing the effects of TCM monomers with the control group based on a random-effects model.

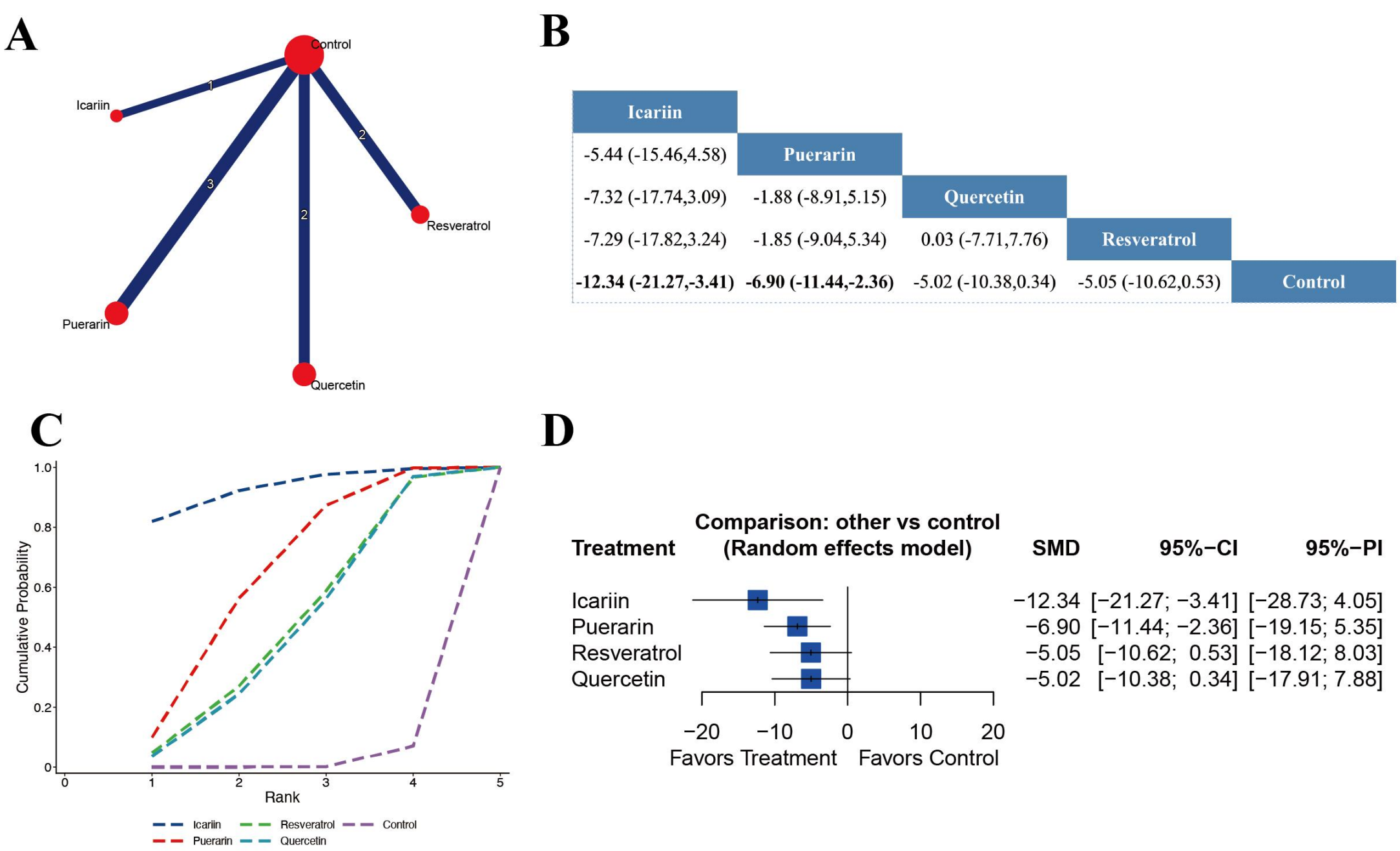

Figure S15 Subgroup analysis: network meta-analysis of FSH levels in animal models with a treatment duration of 21 days or less. (A) Network plot of eligible comparisons. (B) League table summarizing all pairwise comparisons. Bold values represent statistical significance. (C) SUCRA plots showing cumulative ranking probabilities. A larger area under the curve indicates a higher probability of the intervention being top-ranked. (D) Forest plot comparing the effects of TCM monomers with the control group based on a random-effects model.

A

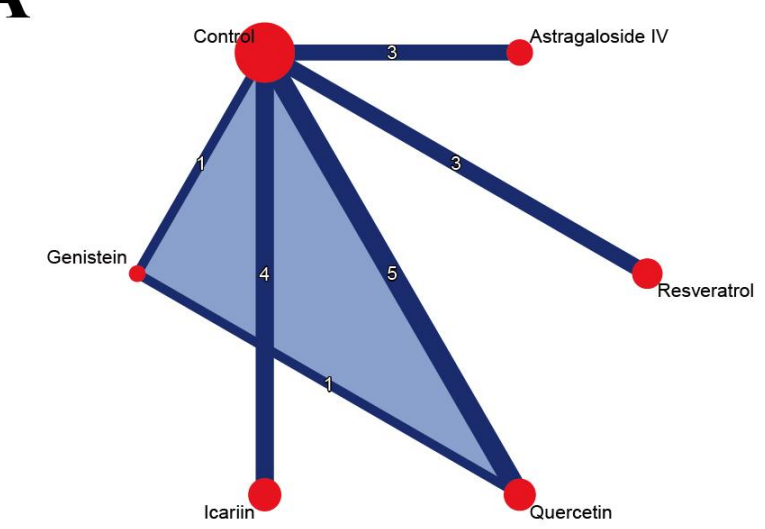

B

**Astragaloside IV**					
3.68 (-1.49,8.84)	**Genistein**				
1.18 (-2.53,4.90)	-2.49 (-7.49,2.50)	**Icariin**			
3.46 (-0.17,7.10)	-0.21 (-4.51,4.09)	2.28 (-1.10,5.66)	**Quercetin**		
1.33 (-2.66,5.32)	-2.35 (-7.55,2.86)	0.15 (-3.61,3.91)	-2.14 (-5.82,1.55)	**Resveratrol**	
**-3.05 (-5.84,-0.26)**	**-6.73 (-11.08,-2.37)**	**-4.23 (-6.68,-1.78)**	**-6.51 (-8.84,-4.18)**	**-4.38 (-7.23,-1.53)**	**Control**

C

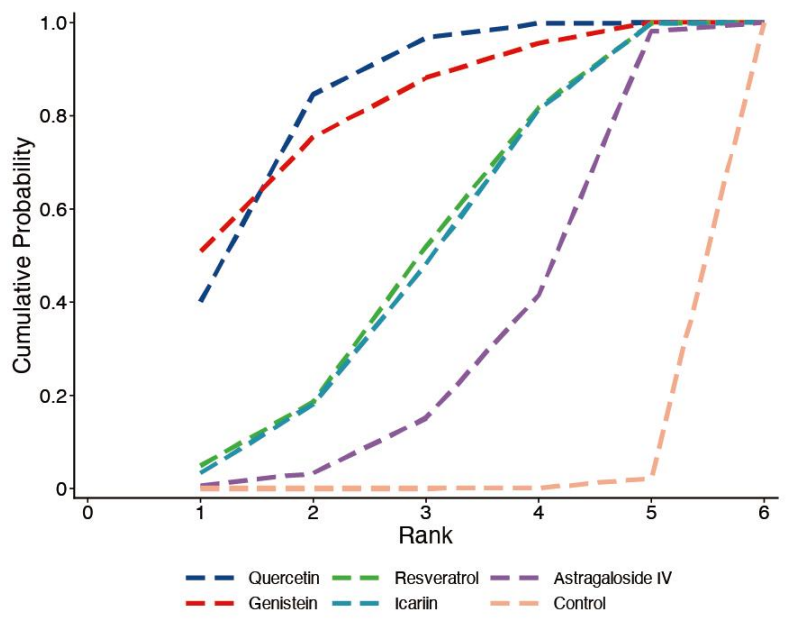

D

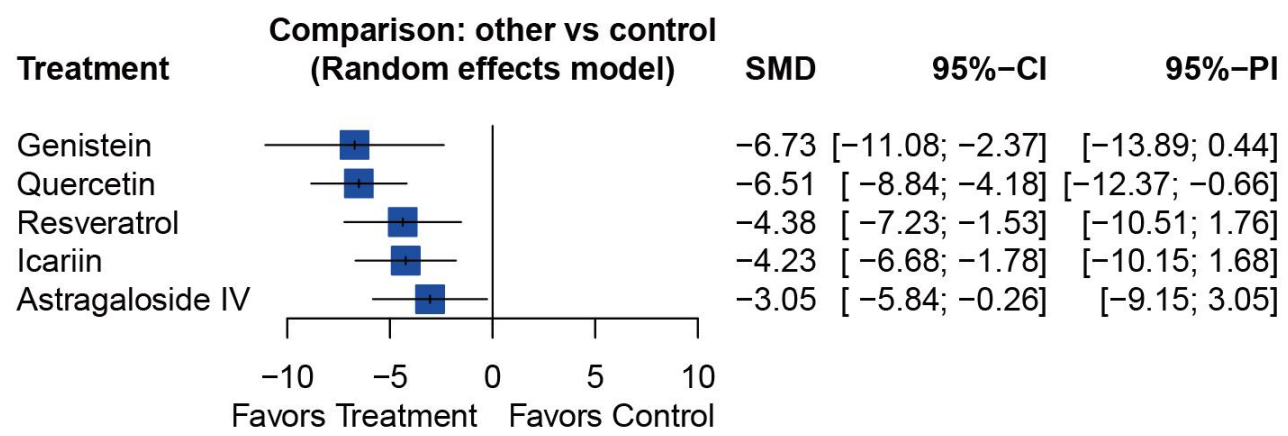

Figure S16 Subgroup analysis: network meta-analysis of FSH levels in animal models with a treatment duration of more than 21 days. (A) Network plot of eligible comparisons. (B) League table summarizing all pairwise comparisons. Bold values represent statistical significance. (C) SUCRA plots showing cumulative ranking probabilities. A larger area under the curve indicates a higher probability of the intervention being top-ranked. (D) Forest plot comparing the effects of TCM monomers with the control group based on a random-effects model.

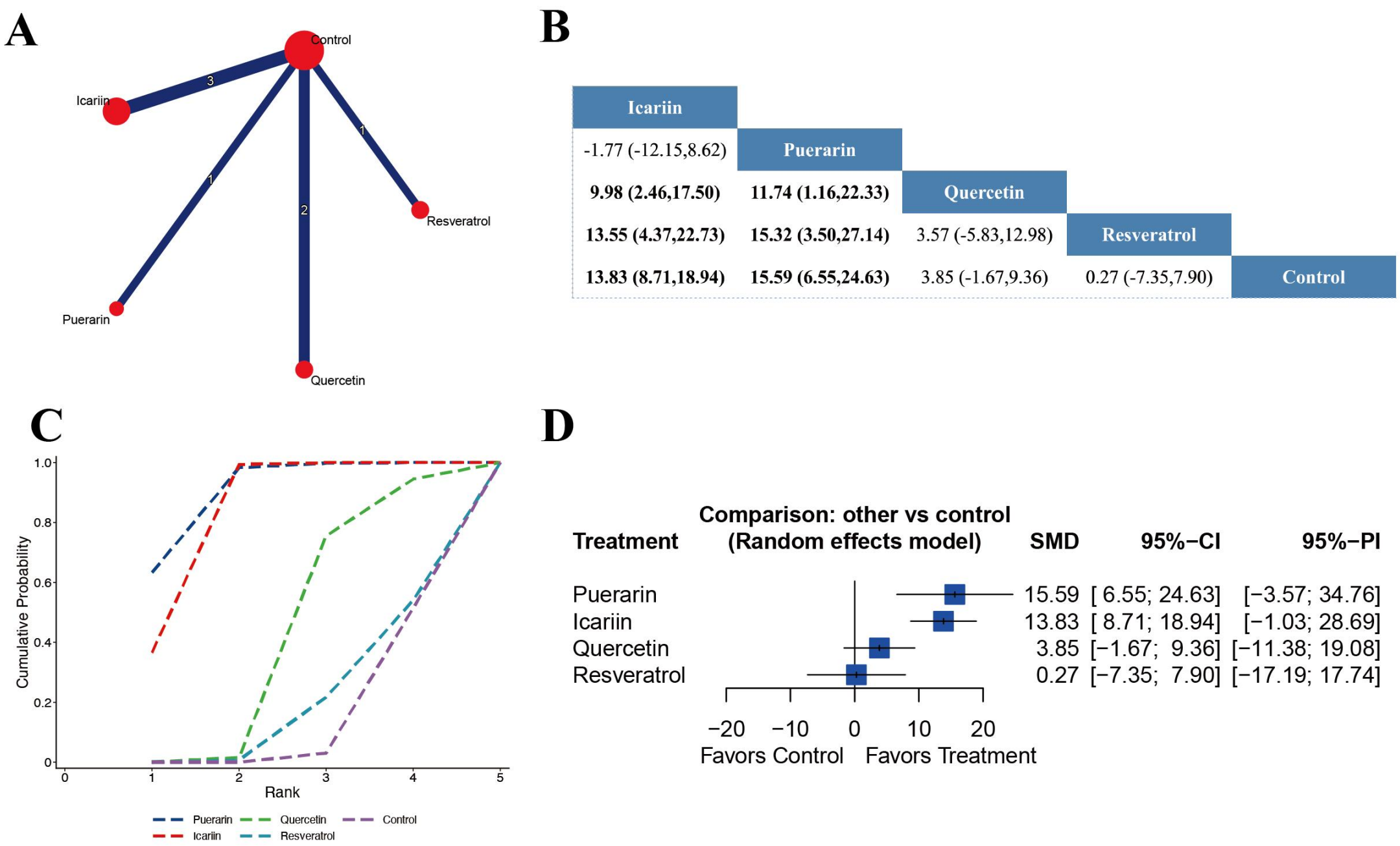

Figure S17 Subgroup analysis: network meta-analysis of AMH levels in mice. (A) Network plot of eligible comparisons. (B) League table summarizing all pairwise comparisons. Bold values represent statistical significance. (C) SUCRA plots showing cumulative ranking probabilities. A larger area under the curve indicates a higher probability of the intervention being top-ranked. (D) Forest plot comparing the effects of TCM monomers with the control group based on a random-effects model.

A

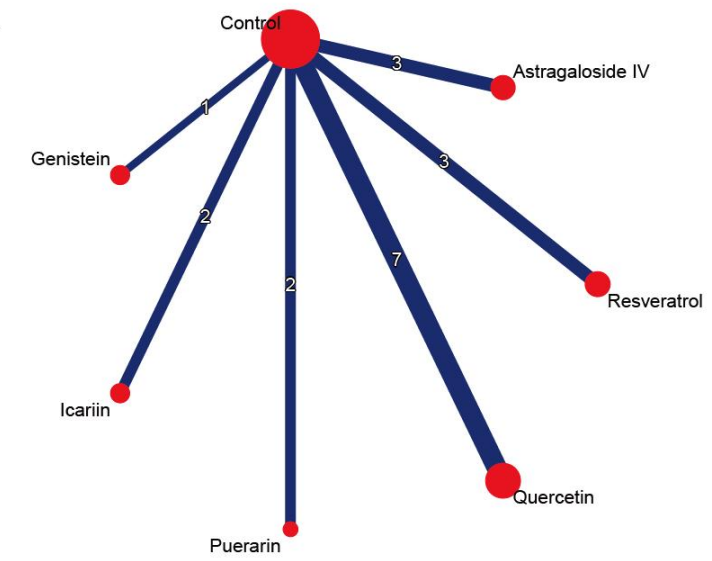

B

Astragaloside IV						
2.27 (-3.54,8.09)	Genistein					
-2.55 (-7.45,2.35)	-4.82 (-11.14,1.50)	Icariin				
-0.09 (-4.91,4.73)	-2.36 (-8.62,3.90)	2.46 (-2.96,7.88)	Puerarin			
-1.11 (-4.74,2.52)	-3.38 (-8.78,2.02)	1.44 (-2.96,5.84)	-1.02 (-5.33,3.29)	Quercetin		
0.84 (-3.36,5.03)	-1.43 (-7.23,4.36)	3.39 (-1.49,8.27)	0.93 (-3.86,5.72)	1.95 (-1.65,5.55)	Resveratrol	
**4.07 (1.09,7.05)**	1.80 (-3.19,6.79)	**6.62 (2.73,10.50)**	**4.16 (0.38,7.94)**	**5.18 (3.12,7.24)**	**3.23 (0.28,6.18)**	Control

C

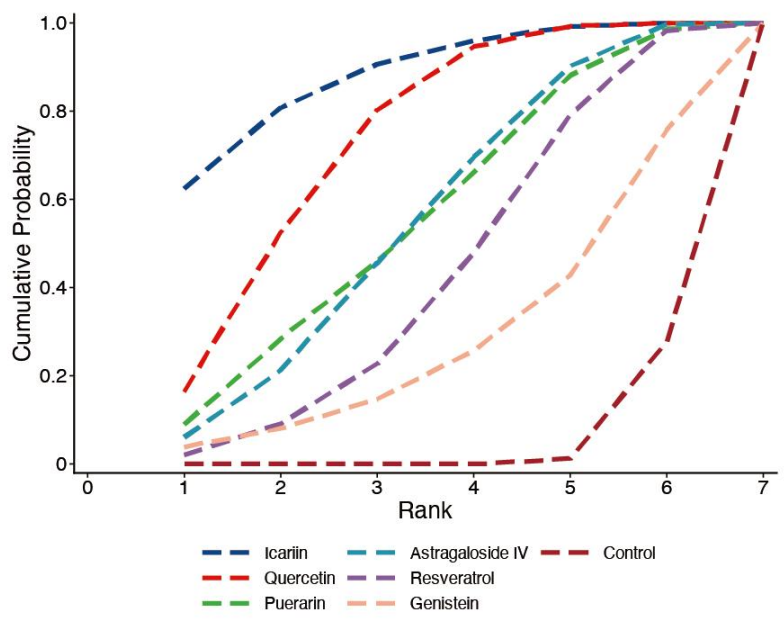

D

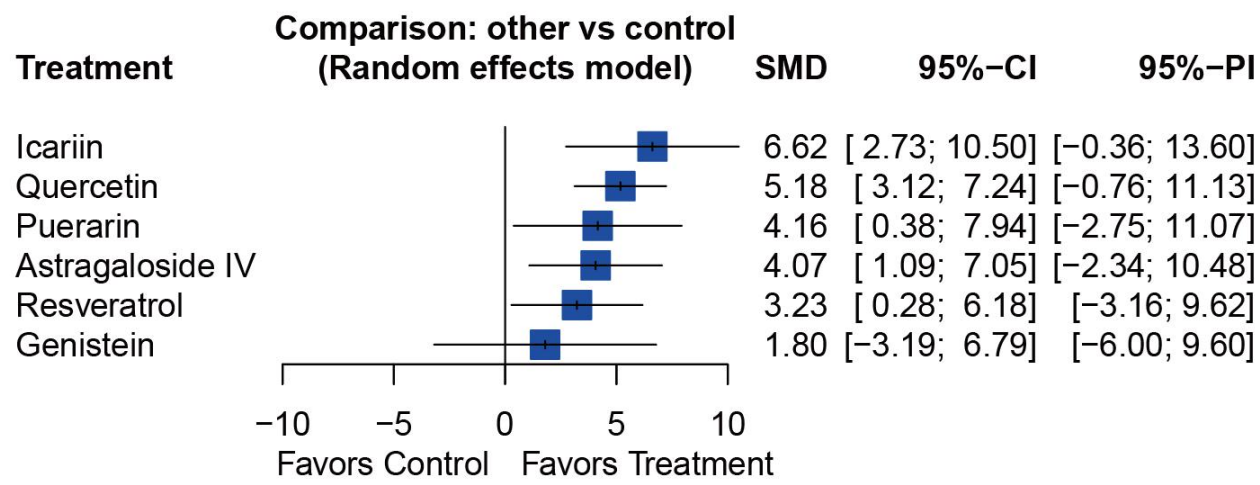

Figure S18 Subgroup analysis: network meta-analysis of AMH levels in rats. (A) Network plot of eligible comparisons. (B) League table summarizing all pairwise comparisons. Bold values represent statistical significance. (C) SUCRA plots showing cumulative ranking probabilities. A larger area under the curve indicates a higher probability of the intervention being top-ranked. (D) Forest plot comparing the effects of TCM monomers with the control group based on a random-effects model.

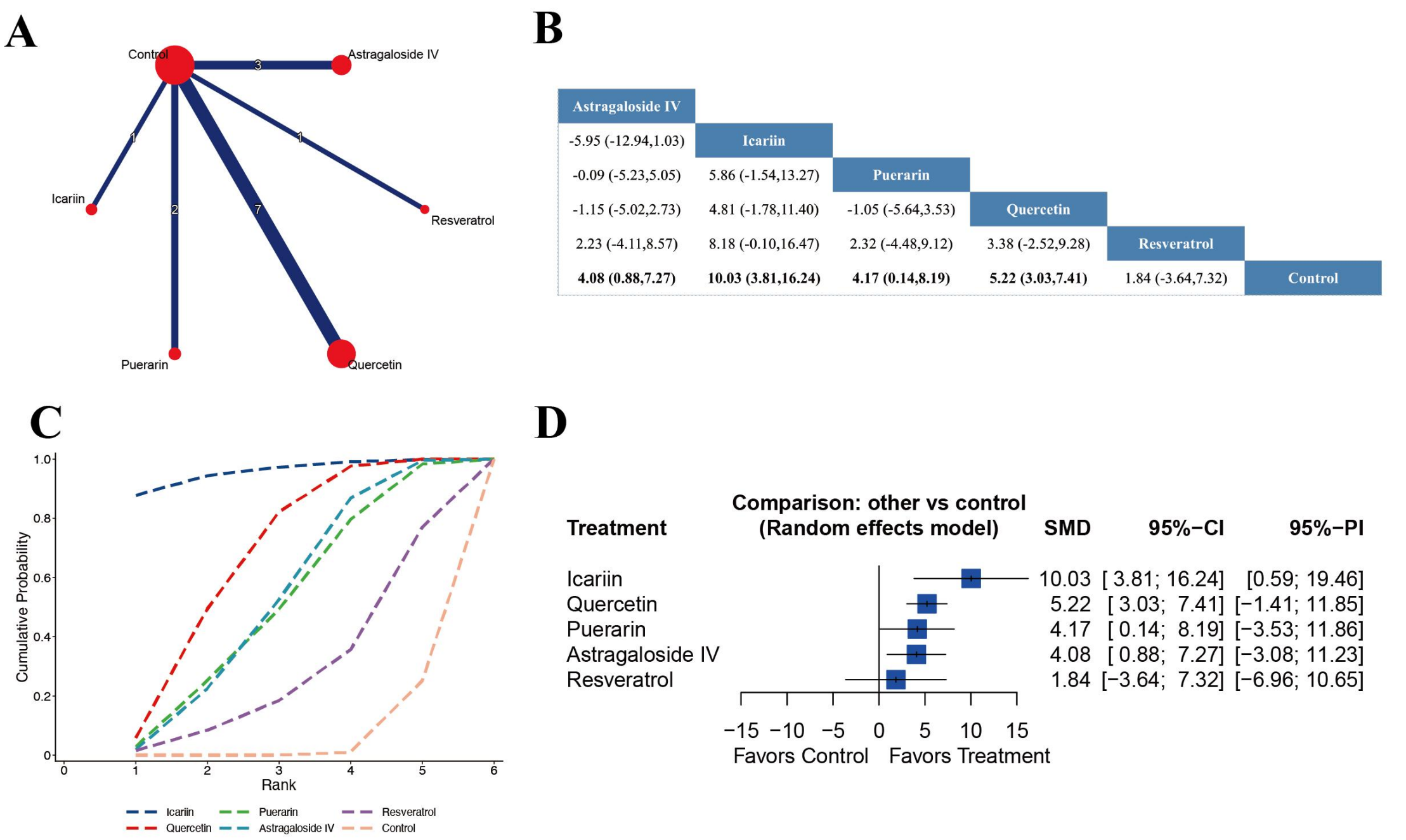

Astragaloside IV					
-5.95 (-12.94,1.03)	Icariin				
-0.09 (-5.23,5.05)	5.86 (-1.54,13.27)	Puerarin			
-1.15 (-5.02,2.73)	4.81 (-1.78,11.40)	-1.05 (-5.64,3.53)	Quercetin		
2.23 (-4.11,8.57)	8.18 (-0.10,16.47)	2.32 (-4.48,9.12)	3.38 (-2.52,9.28)	Resveratrol	
**4.08 (0.88,7.27)**	**10.03 (3.81,16.24)**	**4.17 (0.14,8.19)**	**5.22 (3.03,7.41)**	1.84 (-3.64,7.32)	Control

Figure S19 Subgroup analysis: network meta-analysis of AMH levels in CTX induced animal models. (A) Network plot of eligible comparisons. (B) League table summarizing all pairwise comparisons. Bold values represent statistical significance. (C) SUCRA plots showing cumulative ranking probabilities. A larger area under the curve indicates a higher probability of the intervention being top-ranked. (D) Forest plot comparing the effects of TCM monomers with the control group based on a random-effects model.

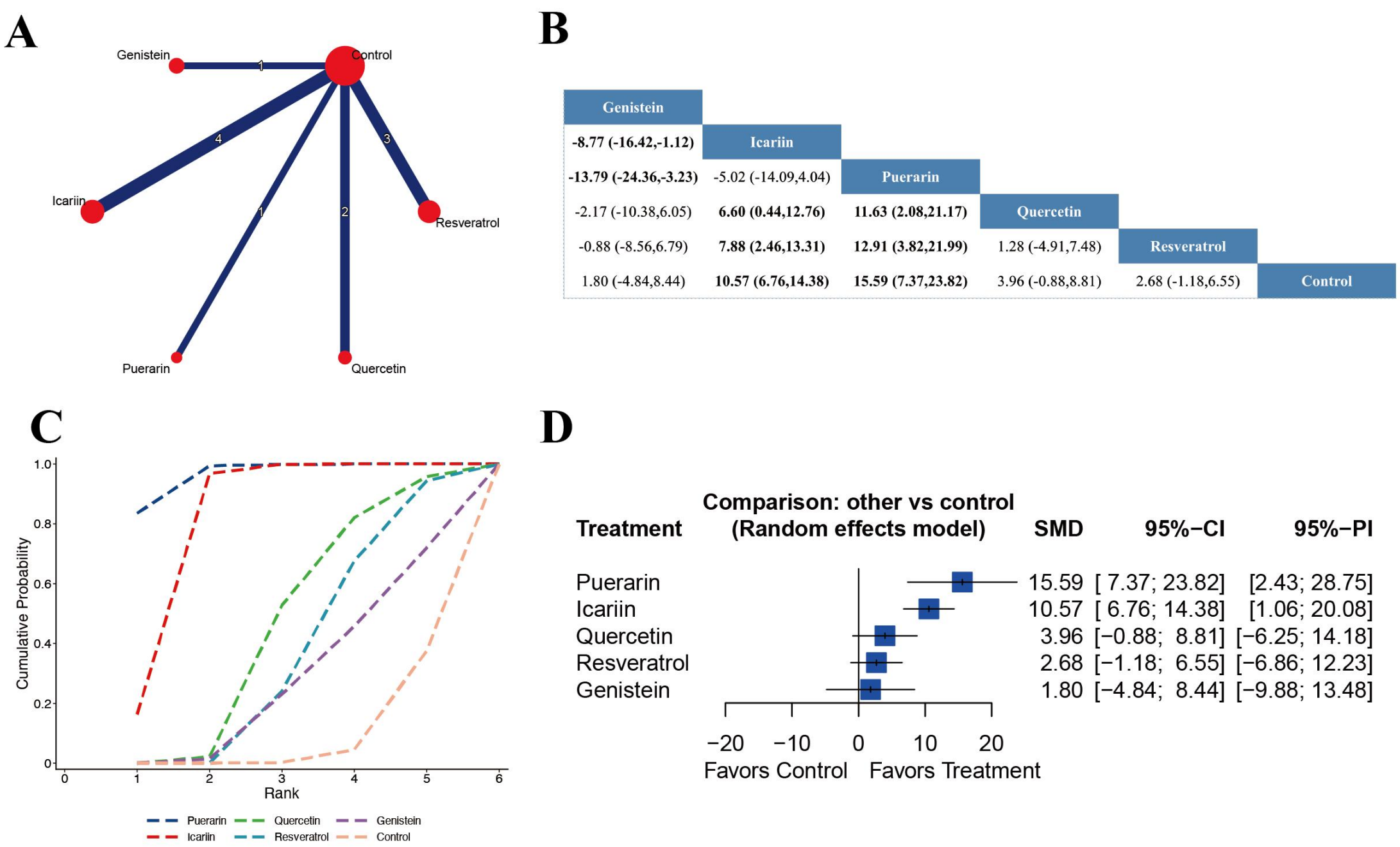

Figure S20 Subgroup analysis: network meta-analysis of AMH levels in non-CTX induced animal models. (A) Network plot of eligible comparisons. (B) League table summarizing all pairwise comparisons. Bold values represent statistical significance. (C) SUCRA plots showing cumulative ranking probabilities. A larger area under the curve indicates a higher probability of the intervention being top-ranked. (D) Forest plot comparing the effects of TCM monomers with the control group based on a random-effects model.

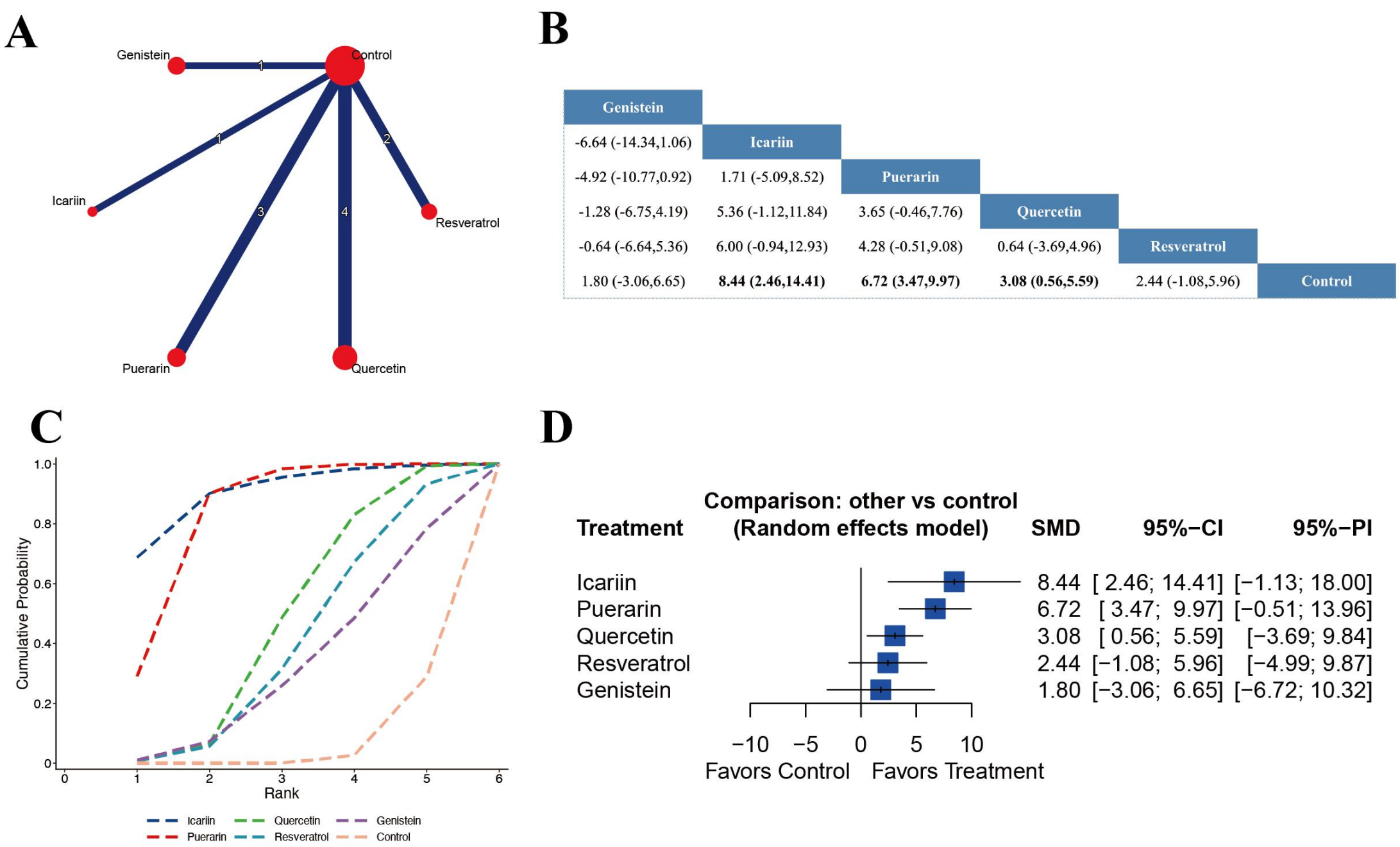

Figure S21 Subgroup analysis: network meta-analysis of AMH levels in animal models with a treatment duration of 21 days or less. (A) Network plot of eligible comparisons. (B) League table summarizing all pairwise comparisons. Bold values represent statistical significance. (C) SUCRA plots showing cumulative ranking probabilities. A larger area under the curve indicates a higher probability of the intervention being top-ranked. (D) Forest plot comparing the effects of TCM monomers with the control group based on a random-effects model.

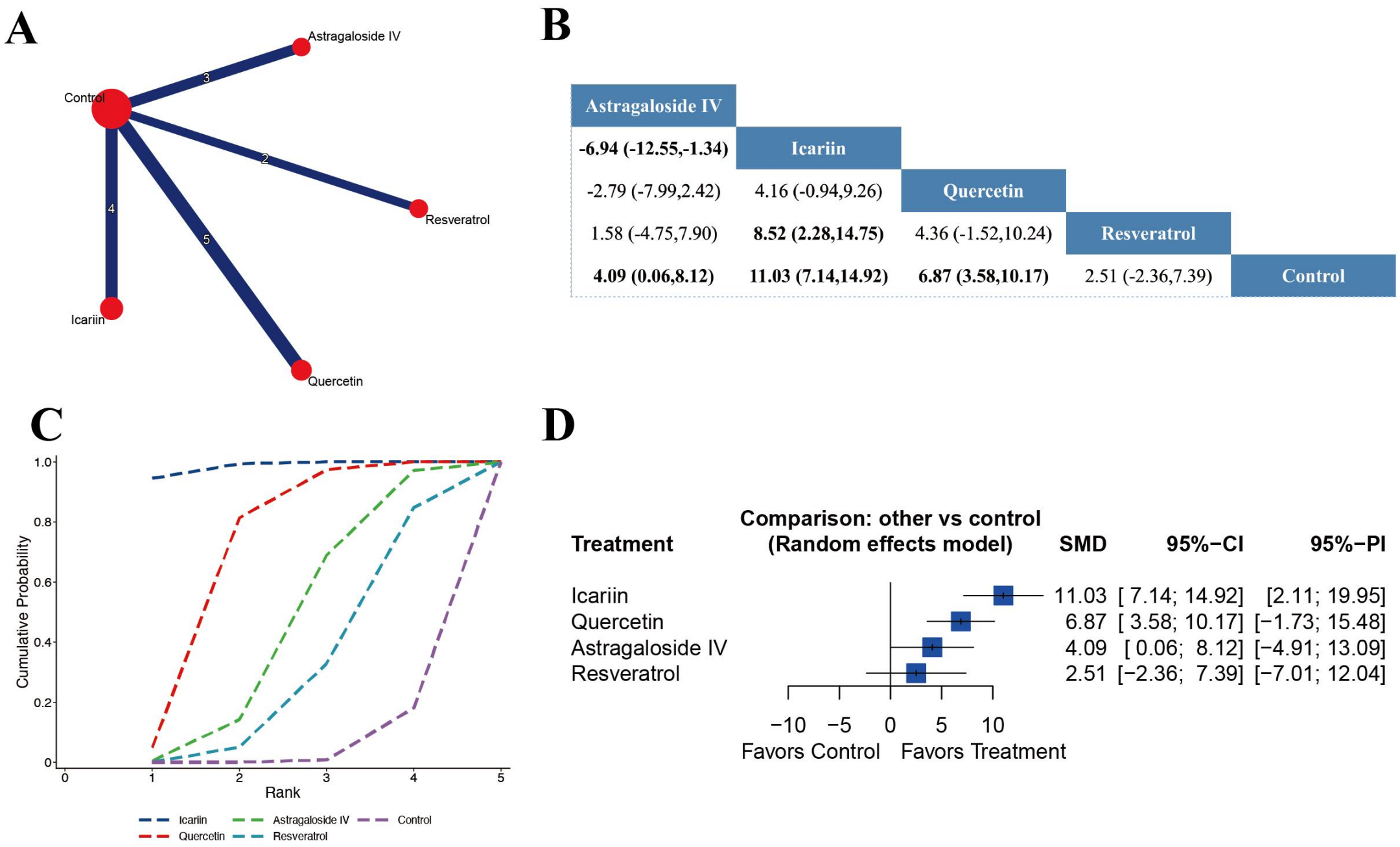

Figure S22 Subgroup analysis: network meta-analysis of AMH levels in animal models with a treatment duration of more than 21 days. (A) Network plot of eligible comparisons. (B) League table summarizing all pairwise comparisons. Bold values represent statistical significance. (C) SUCRA plots showing cumulative ranking probabilities. A larger area under the curve indicates a higher probability of the intervention being top-ranked. (D) Forest plot comparing the effects of TCM monomers with the control group based on a random-effects model.

A

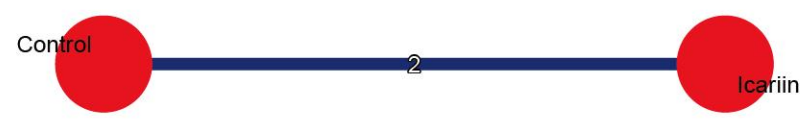

B

Icariin	
0.82 (-1.83,3.48)	Control

C

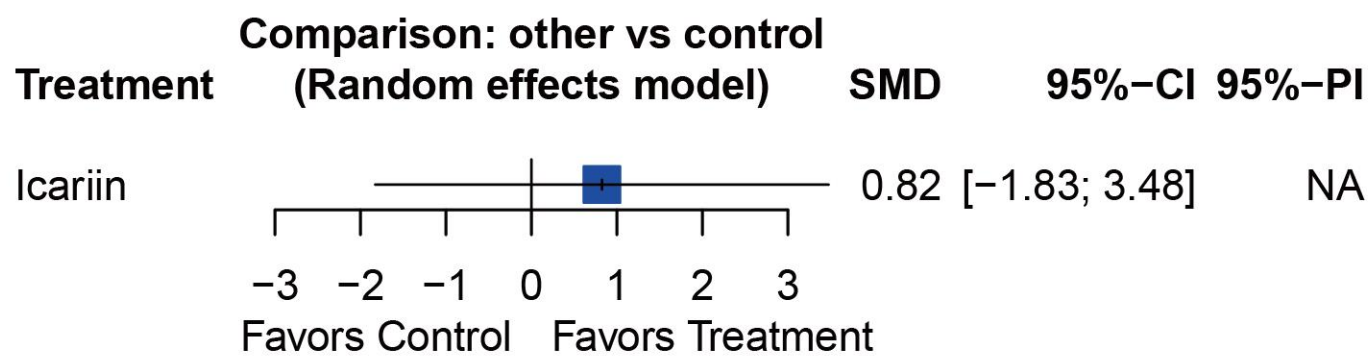

Figure S23 Subgroup analysis: network meta-analysis of AFC in mice. (A) Network plot of eligible comparisons. (B) League table summarizing all pairwise comparisons. Bold values represent statistical significance. (C) Forest plot comparing the effect of TCM monomer with the control group based on a random-effects model.

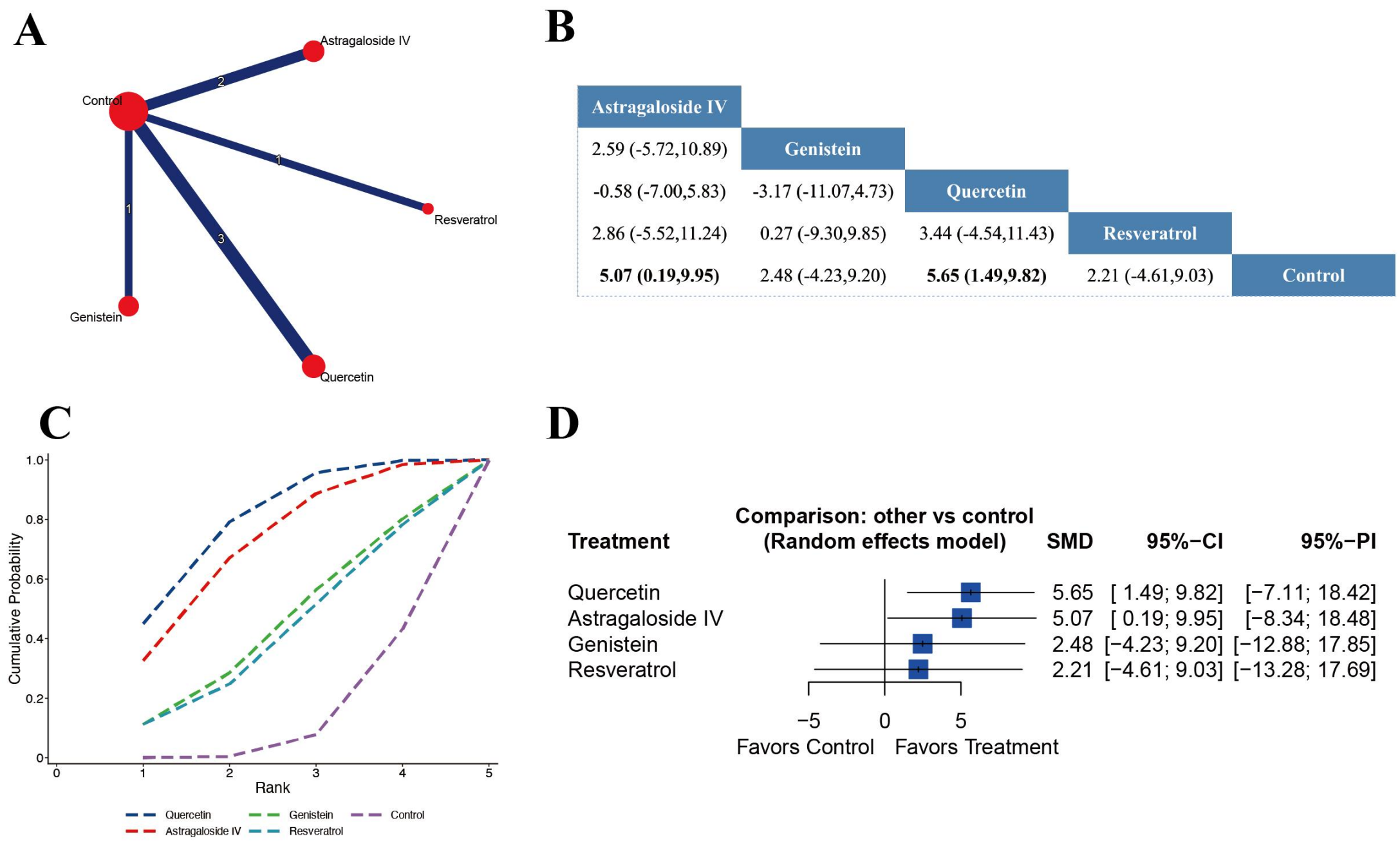

Figure S24 Subgroup analysis: network meta-analysis of AFC in rats. (A) Network plot of eligible comparisons. (B) League table summarizing all pairwise comparisons. Bold values represent statistical significance. (C) SUCRA plots showing cumulative ranking probabilities. A larger area under the curve indicates a higher probability of the intervention being top-ranked. (D) Forest plot comparing the effects of TCM monomers with the control group based on a random-effects model.

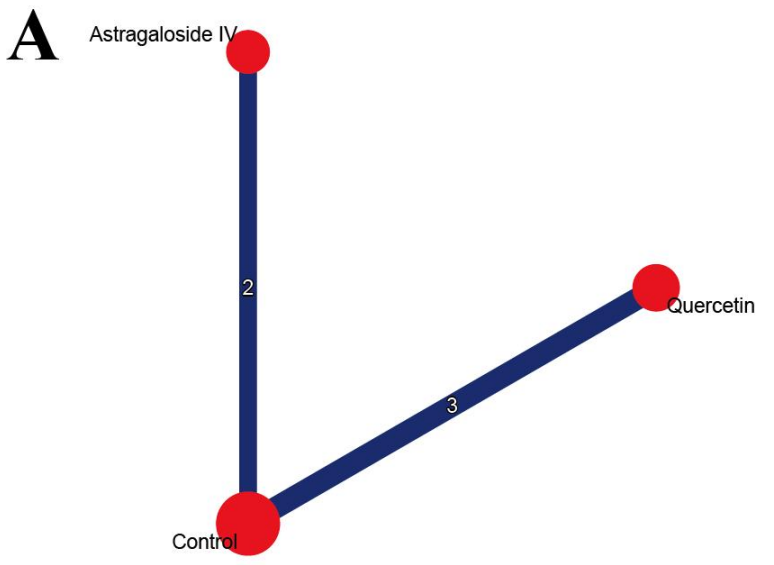

B

Astragaloside IV		
-0.58 (-7.00,5.83)	Quercetin	
**5.07 (0.19,9.95)**	**5.65 (1.49,9.82)**	Control

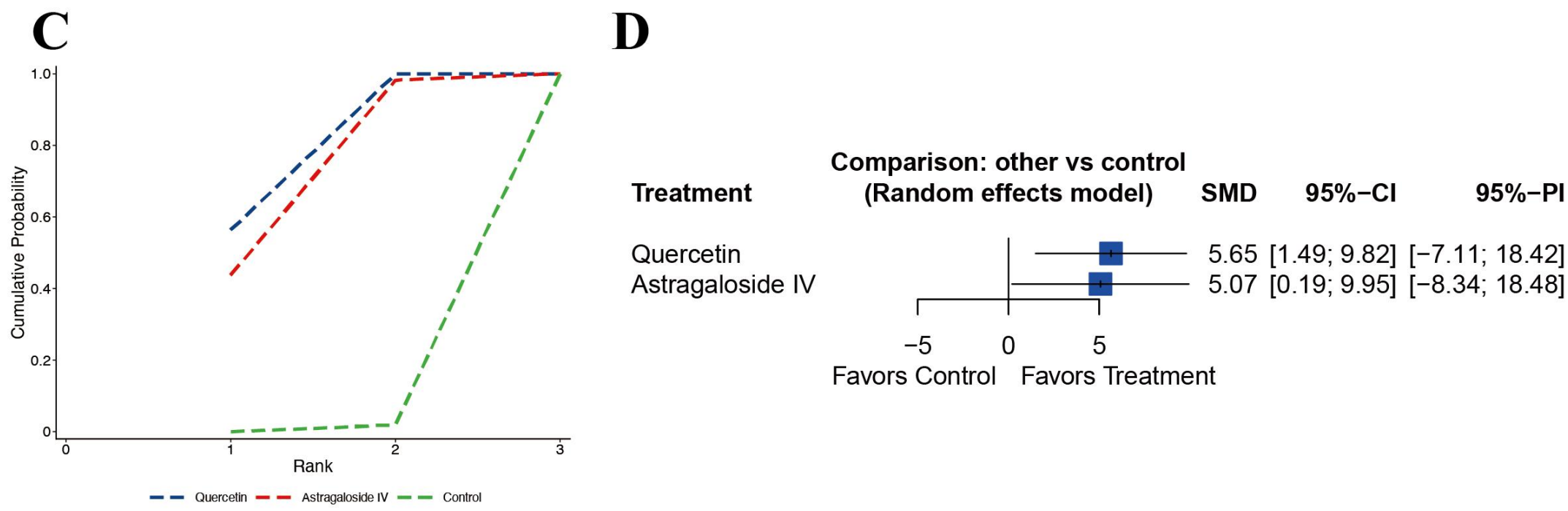

Figure S25 Subgroup analysis: network meta-analysis of AFC in CTX induced animal models. (A) Network plot of eligible comparisons. (B) League table summarizing all pairwise comparisons. Bold values represent statistical significance. (C) SUCRA plots showing cumulative ranking probabilities. A larger area under the curve indicates a higher probability of the intervention being top-ranked. (D) Forest plot comparing the effects of TCM monomers with the control group based on a random-effects model.

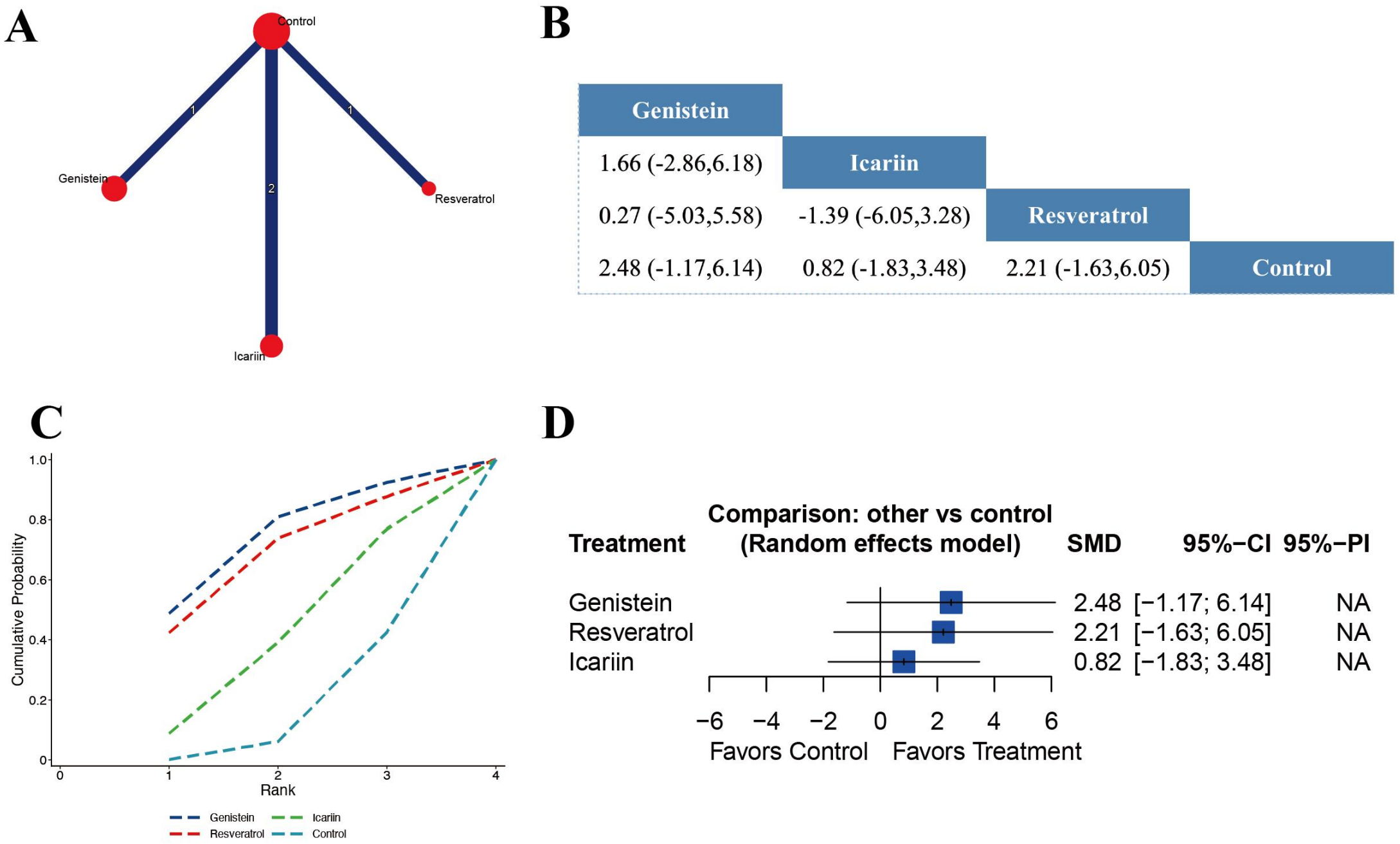

Genistein			
1.66 (-2.86,6.18)	Icariin		
0.27 (-5.03,5.58)	-1.39 (-6.05,3.28)	Resveratrol	
2.48 (-1.17,6.14)	0.82 (-1.83,3.48)	2.21 (-1.63,6.05)	Control

Figure S26 Subgroup analysis: network meta-analysis of AFC in non-CTX induced animal models. (A) Network plot of eligible comparisons. (B) League table summarizing all pairwise comparisons. Bold values represent statistical significance. (C) SUCRA plots showing cumulative ranking probabilities. A larger area under the curve indicates a higher probability of the intervention being top-ranked. (D) Forest plot comparing the effects of TCM monomers with the control group based on a random-effects model.

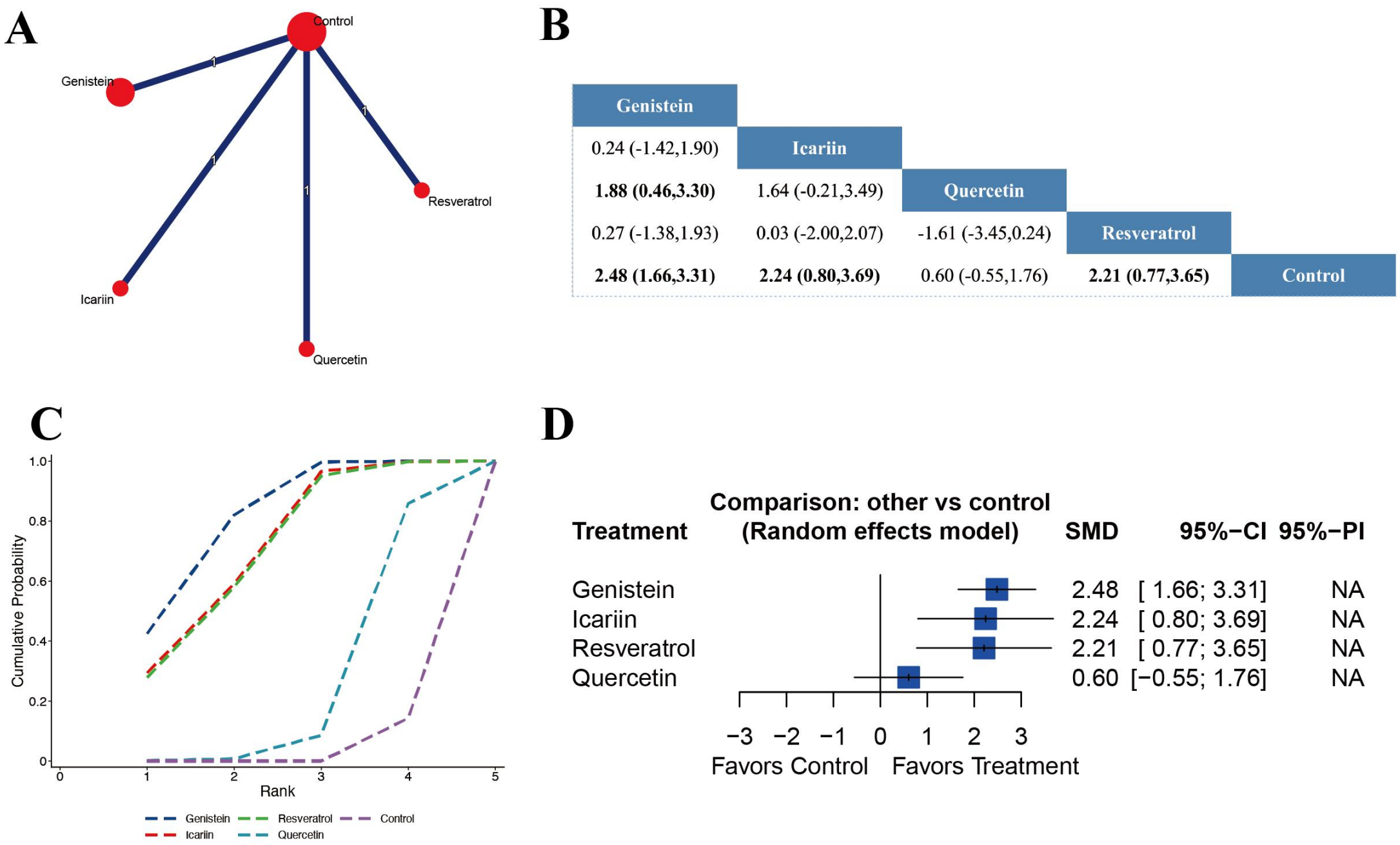

Figure S27 Subgroup analysis: network meta-analysis of AFC in animal models with a treatment duration of 21 days or less. (A) Network plot of eligible comparisons. (B) League table summarizing all pairwise comparisons. Bold values represent statistical significance. (C) SUCRA plots showing cumulative ranking probabilities. A larger area under the curve indicates a higher probability of the intervention being top-ranked. (D) Forest plot comparing the effects of TCM monomers with the control group based on a random-effects model.

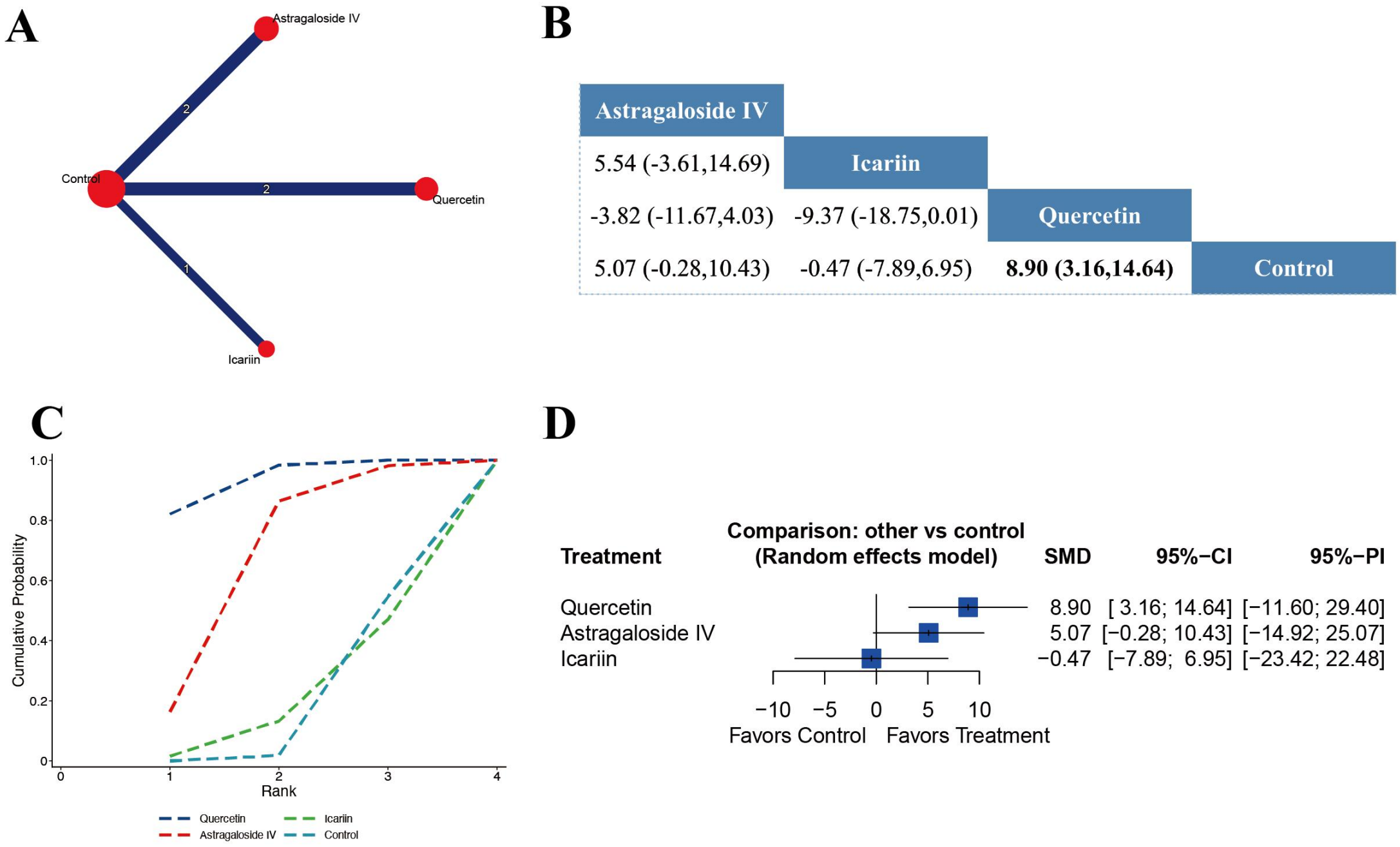

Figure S28 Subgroup analysis: network meta-analysis of AFC in animal models with a treatment duration of more than 21 days. (A) Network plot of eligible comparisons. (B) League table summarizing all pairwise comparisons. Bold values represent statistical significance. (C) SUCRA plots showing cumulative ranking probabilities. A larger area under the curve indicates a higher probability of the intervention being top-ranked. (D) Forest plot comparing the effects of TCM monomers with the control group based on a random-effects model.

A

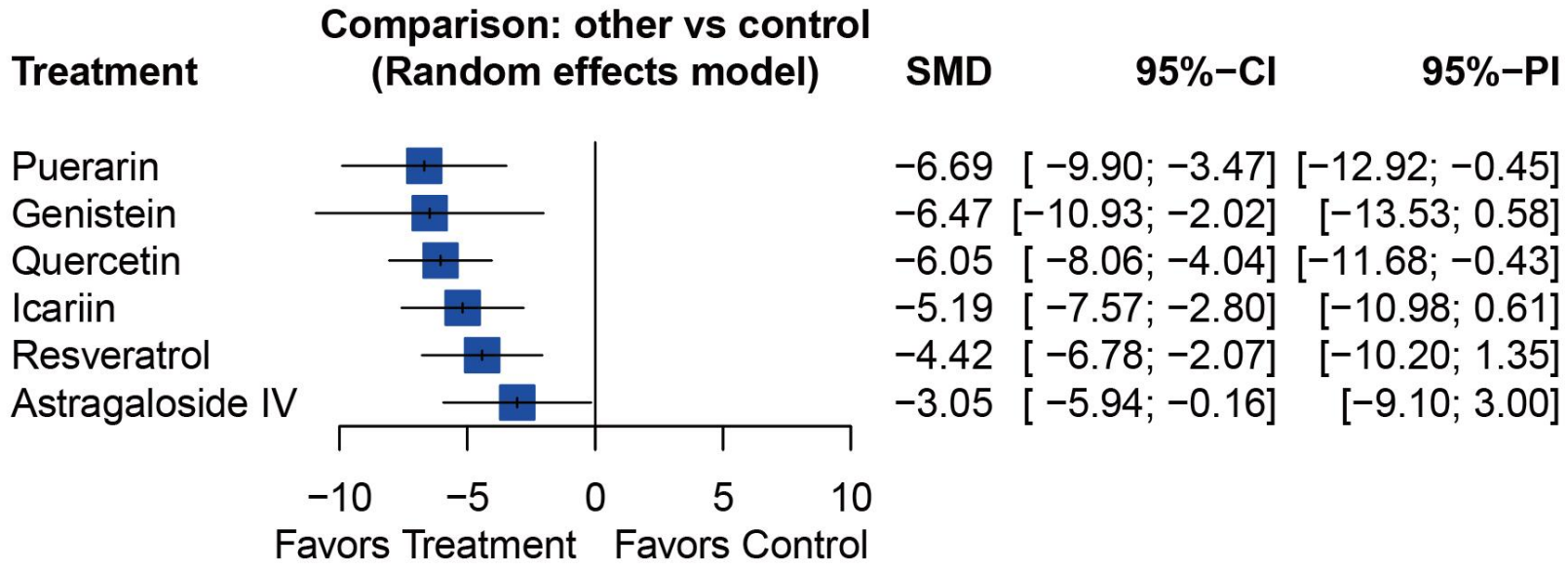

B

**Astragaloside IV**						
3.42 (-1.89,8.74)	**Genistein**					
2.14 (-1.61,5.89)	-1.29 (-6.34,3.77)	**Icariin**				
3.64 (-0.68,7.96)	0.21 (-5.28,5.71)	1.50 (-2.50,5.51)	**Puerarin**			
3.00 (-0.52,6.52)	-0.42 (-4.84,4.00)	0.87 (-2.26,3.99)	-0.64 (-4.43,3.15)	**Quercetin**		
1.37 (-2.36,5.11)	-2.05 (-7.09,2.99)	-0.76 (-4.12,2.60)	-2.26 (-6.25,1.72)	-1.63 (-4.73,1.47)	**Resveratrol**	
**-3.05 (-5.94,-0.16)**	**-6.47 (-10.93,-2.02)**	**-5.19 (-7.57,-2.80)**	**-6.69 (-9.90,-3.47)**	**-6.05 (-8.06,-4.04)**	**-4.42 (-6.78,-2.07)**	**Control**

Figure S29 Sensitivity analysis of FSH excluding small-sample studies ($n < 5$ animals per group). (A) Forest plot comparing the effects of TCM monomers with the control group based on a random-effects model. (B) League table summarizing all pairwise comparisons. Data are expressed as SMDs with 95% CIs.

A

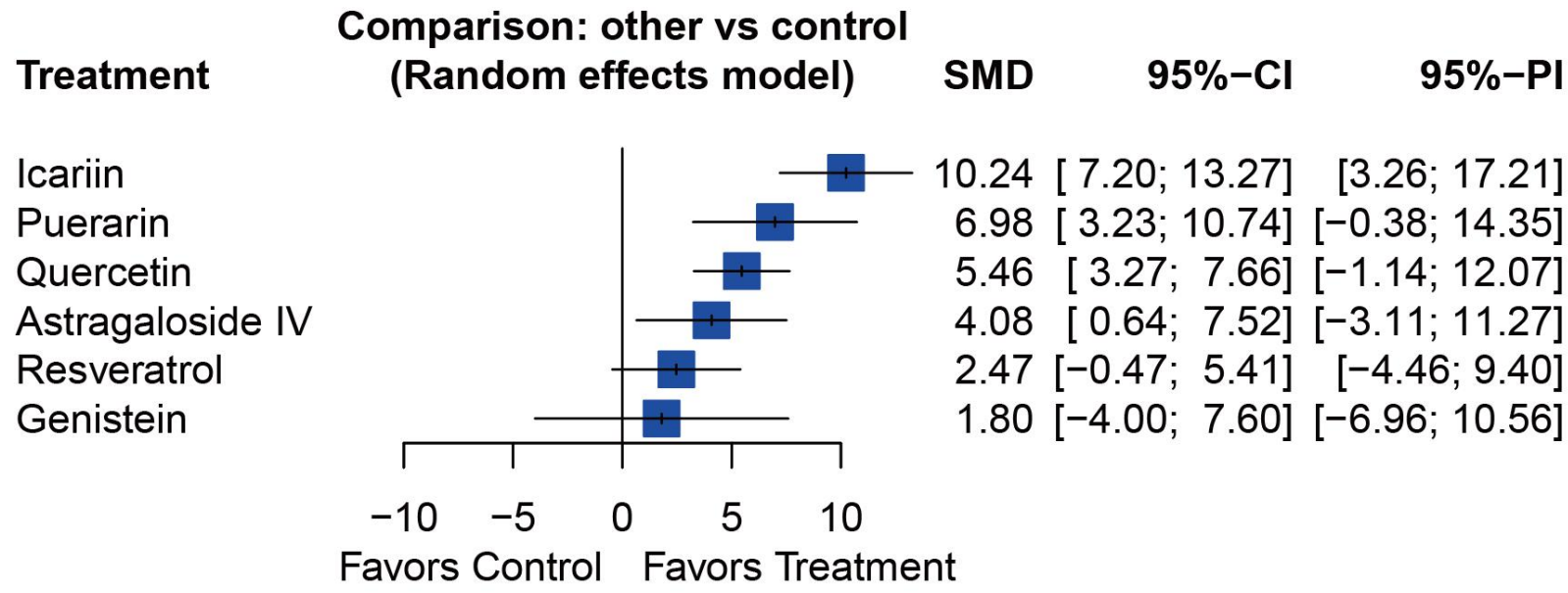

B

Astragaloside IV						
2.28 (-4.46,9.03)	Genistein					
**-6.16 (-10.74,-1.57)**	**-8.44 (-14.99,-1.89)**	Icariin				
-2.90 (-8.00,2.19)	-5.19 (-12.10,1.72)	3.25 (-1.58,8.08)	Puerarin			
-1.38 (-5.47,2.70)	-3.67 (-9.87,2.54)	**4.77 (1.02,8.52)**	1.52 (-2.83,5.87)	Quercetin		
1.61 (-2.91,6.13)	-0.67 (-7.18,5.83)	**7.77 (3.54,11.99)**	4.51 (-0.25,9.28)	2.99 (-0.68,6.66)	Resveratrol	
**4.08 (0.64,7.52)**	1.80 (-4.00,7.60)	**10.24 (7.20,13.27)**	**6.98 (3.23,10.74)**	**5.46 (3.27,7.66)**	2.47 (-0.47,5.41)	Control

Figure S30 Sensitivity analysis of AMH excluding small-sample studies ($n < 5$ animals per group). (A) Forest plot comparing the effects of TCM monomers with the control group based on a random-effects model. (B) League table summarizing all pairwise comparisons. Data are expressed as SMDs with 95% CIs.

A

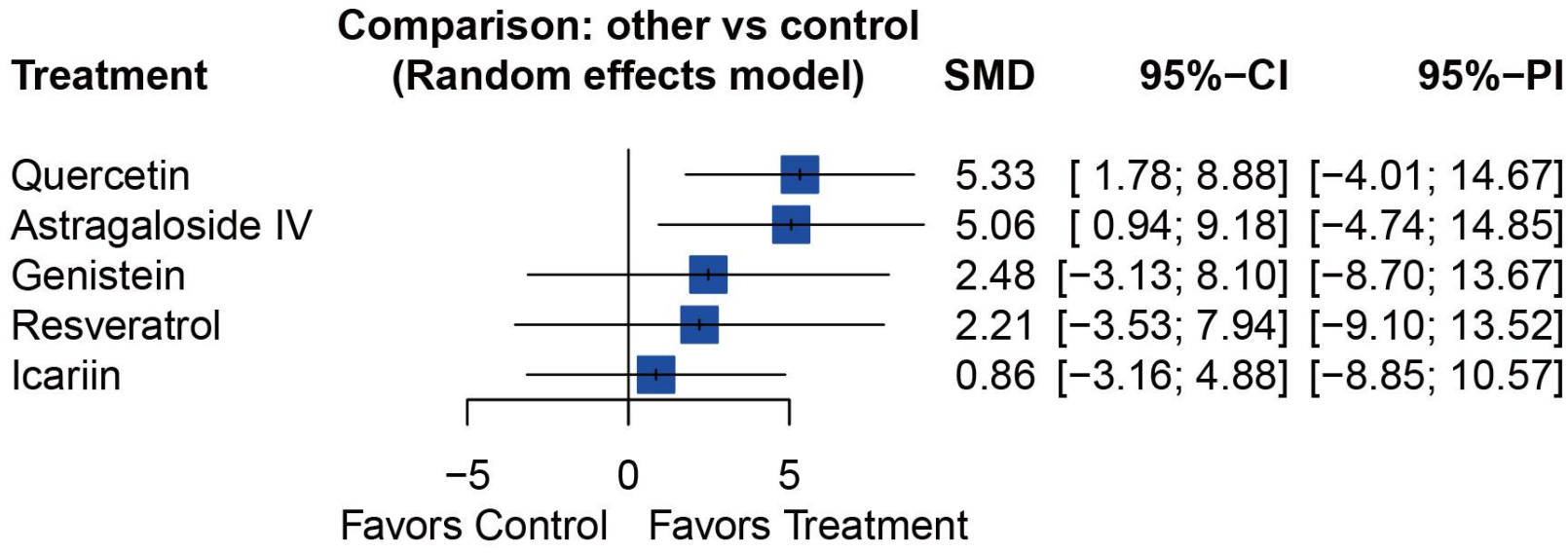

B

**Astragaloside IV**					
2.57 (-4.39,9.54)	**Genistein**				
4.20 (-1.56,9.95)	1.62 (-5.28,8.53)	**Icariin**			
-0.27 (-5.71,5.17)	-2.85 (-9.49,3.80)	-4.47 (-9.83,0.89)	**Quercetin**		
2.85 (-4.21,9.91)	0.27 (-7.75,8.30)	-1.35 (-8.35,5.65)	3.12 (-3.63,9.87)	**Resveratrol**	
**5.06 (0.94,9.18)**	2.48 (-3.13,8.10)	0.86 (-3.16,4.88)	**5.33 (1.78,8.88)**	2.21 (-3.53,7.94)	**Control**

Figure S31 Sensitivity analysis of AFC excluding small-sample studies (n < 5 animals per group). (A) Forest plot comparing the effects of TCM monomers with the control group based on a random-effects model. (B) League table summarizing all pairwise comparisons. Data are expressed as SMDs with 95% CIs.

A

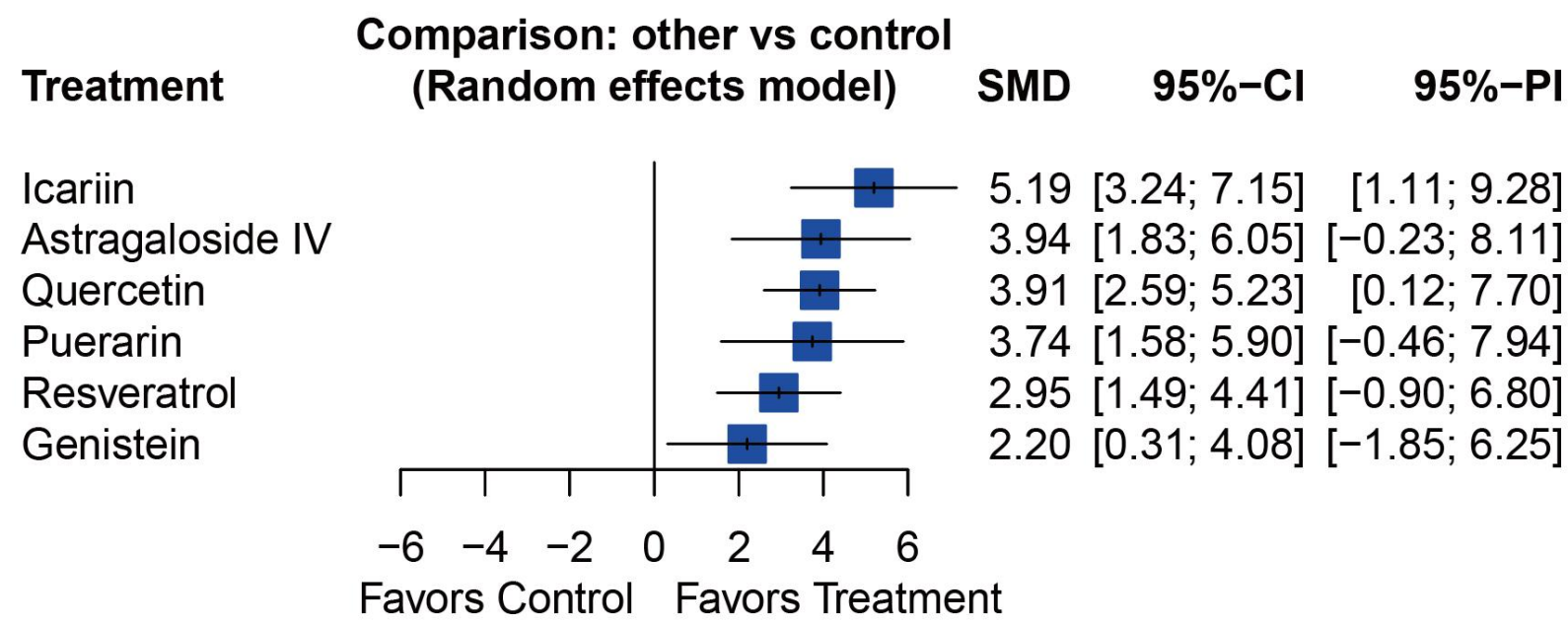

B

**Astragaloside IV**						
1.74 (-1.09,4.57)	**Genistein**					
-1.25 (-4.13,1.62)	**-3.00 (-5.72,-0.28)**	**Icariin**				
0.20 (-2.81,3.22)	-1.54 (-4.41,1.32)	1.46 (-1.46,4.37)	**Puerarin**			
0.03 (-2.46,2.52)	-1.71 (-3.86,0.43)	1.28 (-1.07,3.64)	-0.17 (-2.70,2.36)	**Quercetin**		
0.99 (-1.57,3.56)	-0.75 (-3.14,1.64)	2.25 (-0.20,4.69)	0.79 (-1.81,3.40)	0.96 (-1.00,2.93)	**Resveratrol**	
**3.94 (1.83,6.05)**	**2.20 (0.31,4.08)**	**5.19 (3.24,7.15)**	**3.74 (1.58,5.90)**	**3.91 (2.59,5.23)**	**2.95 (1.49,4.41)**	**Control**

Figure S32 Sensitivity analysis of E_2 excluding small-sample studies ($n < 5$ animals per group). (A) Forest plot comparing the effects of TCM monomers with the control group based on a random-effects model. (B) League table summarizing all pairwise comparisons. Data are expressed as SMDs with 95% CIs.

A

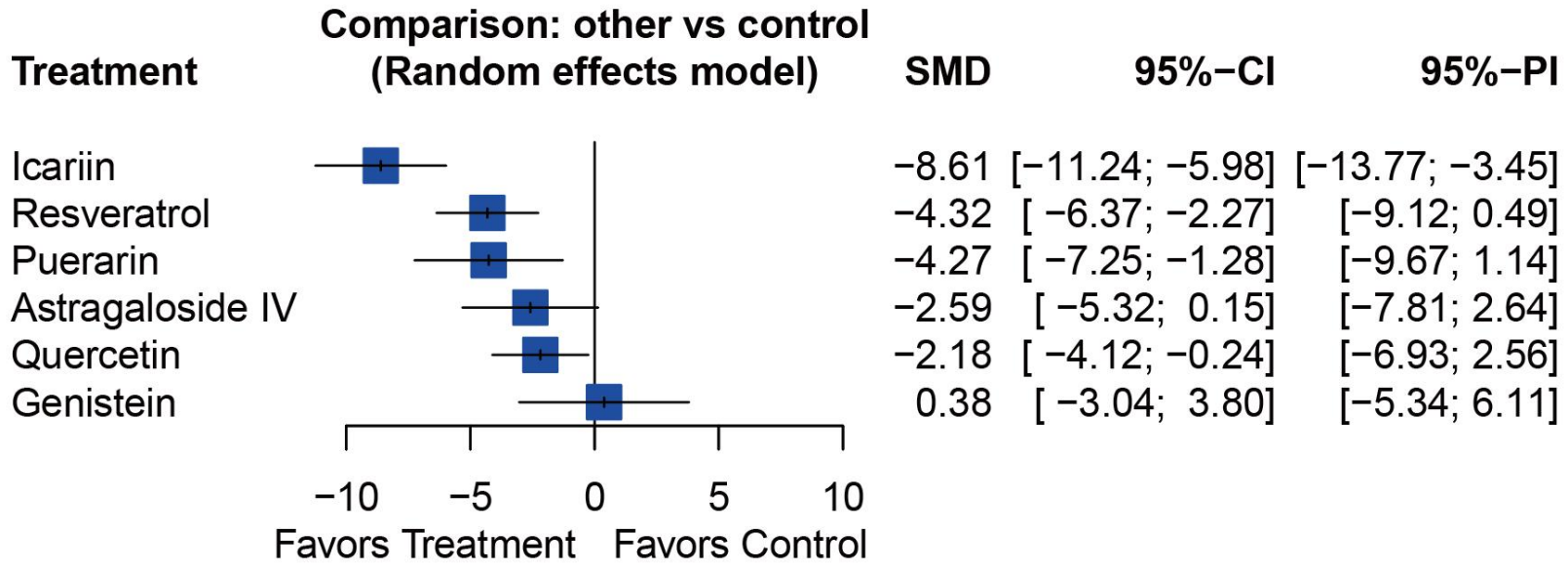

B

Astragaloside IV						
-2.97 (-7.35,1.41)	Genistein					
**6.02 (2.23,9.82)**	**8.99 (4.67,13.31)**	Icariin				
1.68 (-2.37,5.73)	**4.65 (0.10,9.19)**	**-4.34 (-8.32,-0.36)**	Puerarin			
-0.40 (-3.75,2.95)	2.56 (-0.88,6.00)	**-6.43 (-9.69,-3.16)**	-2.08 (-5.64,1.48)	Quercetin		
1.73 (-1.69,5.15)	**4.70 (0.71,8.69)**	**-4.29 (-7.63,-0.95)**	0.05 (-3.57,3.68)	2.14 (-0.69,4.96)	Resveratrol	
-2.59 (-5.32,0.15)	0.38 (-3.04,3.80)	**-8.61 (-11.24,-5.98)**	**-4.27 (-7.25,-1.28)**	**-2.18 (-4.12,-0.24)**	**-4.32 (-6.37,-2.27)**	Control

Figure S33 Sensitivity analysis of LH excluding small-sample studies (*n* < 5 animals per group). (A) Forest plot comparing the effects of TCM monomers with the control group based on a random-effects model. (B) League table summarizing all pairwise comparisons. Data are expressed as SMDs with 95% CIs.

A

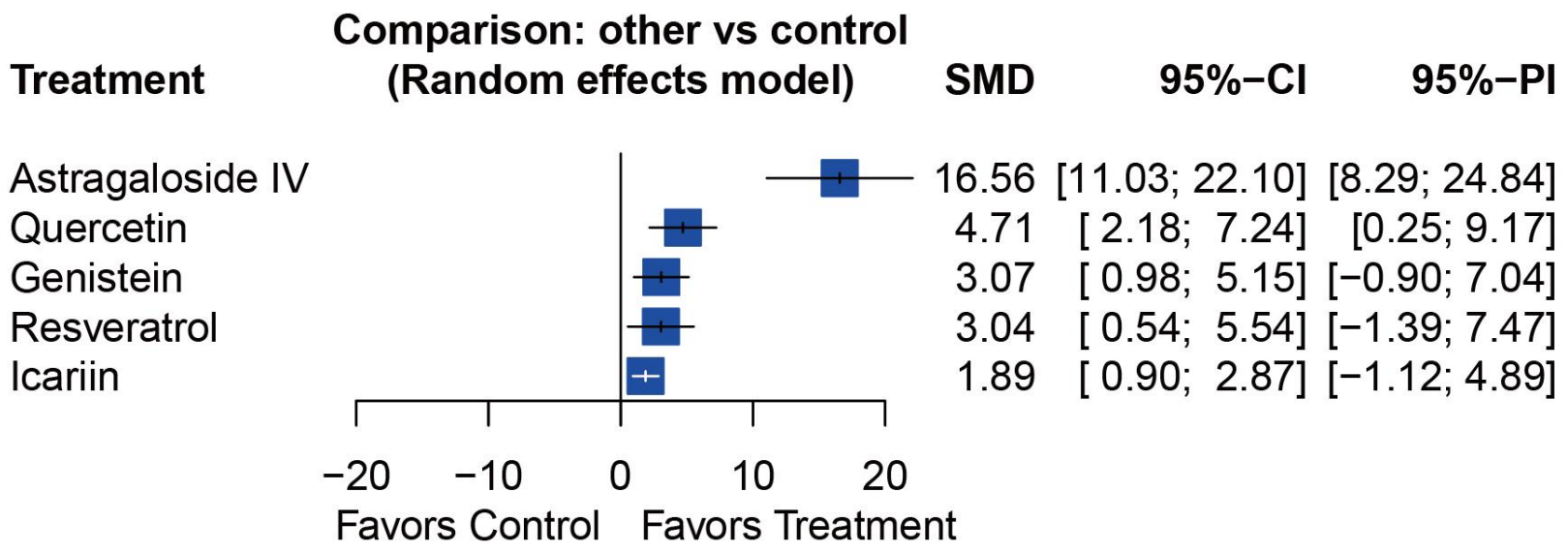

B

Astragaloside IV					
**13.49 (7.58,19.41)**	Genistein				
**14.68 (9.06,20.30)**	1.18 (-1.12,3.49)	Icariin			
**11.86 (5.77,17.94)**	-1.64 (-4.92,1.64)	**-2.82 (-5.54,-0.10)**	Quercetin		
**13.52 (7.45,19.60)**	0.03 (-3.23,3.29)	-1.15 (-3.85,1.54)	1.67 (-1.89,5.23)	Resveratrol	
**16.56 (11.03,22.10)**	**3.07 (0.98,5.15)**	**1.89 (0.90,2.87)**	**4.71 (2.18,7.24)**	**3.04 (0.54,5.54)**	Control

Figure S34 Sensitivity analysis of primordial follicle excluding small-sample studies (n < 5 animals per group). (A) Forest plot comparing the effects of TCM monomers with the control group based on a random-effects model. (B) League table summarizing all pairwise comparisons. Data are expressed as SMDs with 95% CIs.

A

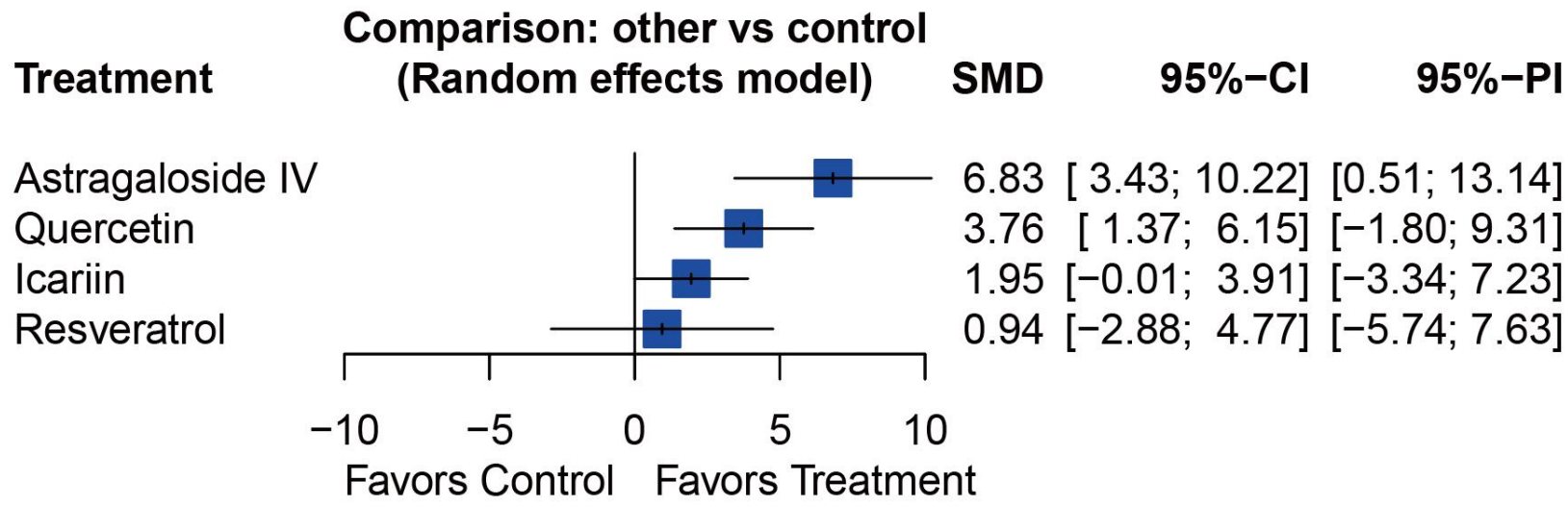

B

Astragaloside IV				
**4.88 (0.96,8.80)**	Icariin			
3.07 (-1.08,7.22)	-1.81 (-4.90,1.28)	Quercetin		
**5.88 (0.77,11.00)**	1.00 (-3.30,5.30)	2.81 (-1.70,7.32)	Resveratrol	
**6.83 (3.43,10.22)**	1.95 (-0.01,3.91)	**3.76 (1.37,6.15)**	0.94 (-2.88,4.77)	Control

Figure S35 Sensitivity analysis of primary follicle excluding small-sample studies (n < 5 animals per group). (A) Forest plot comparing the effects of TCM monomers with the control group based on a random-effects model. (B) League table summarizing all pairwise comparisons. Data are expressed as SMDs with 95% CIs.

A

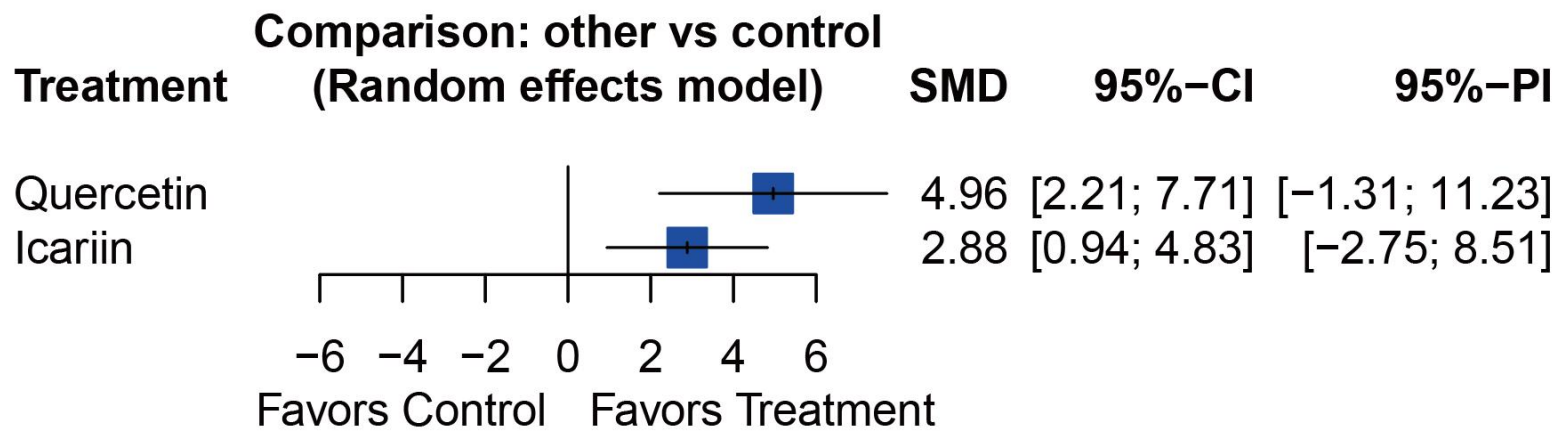

B

**Icariin**		
-2.08 (-5.45,1.29)	**Quercetin**	
**2.88 (0.94,4.83)**	**4.96 (2.21,7.71)**	**Control**

Figure S36 Sensitivity analysis of secondary follicle excluding small-sample studies ($n < 5$ animals per group). (A) Forest plot comparing the effects of TCM monomers with the control group based on a random-effects model. (B) League table summarizing all pairwise comparisons. Data are expressed as SMDs with 95% CIs.

A

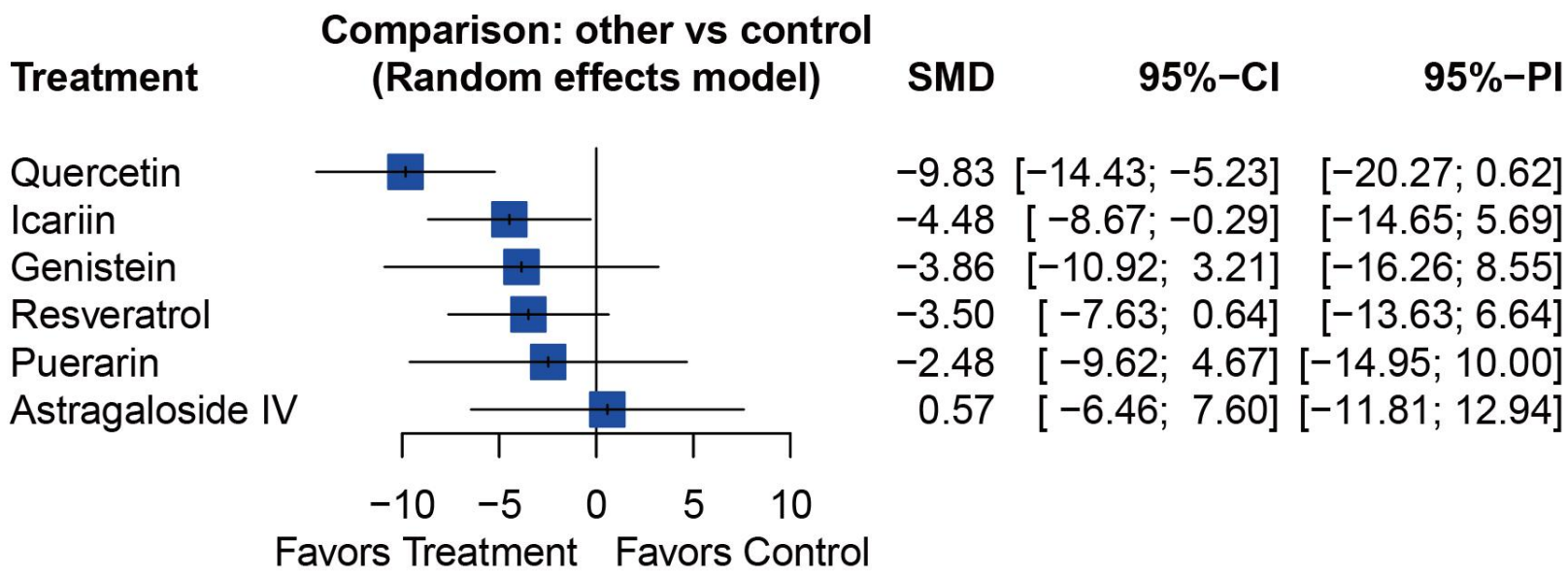

B

**Astragaloside IV**						
4.42 (-5.54,14.39)	**Genistein**					
5.05 (-3.14,13.23)	0.62 (-7.59,8.84)	**Icariin**				
3.05 (-6.98,13.07)	-1.38 (-11.43,8.67)	-2.00 (-10.28,6.28)	**Puerarin**			
**10.40 (1.99,18.80)**	5.97 (-2.46,14.40)	5.35 (-0.88,11.57)	7.35 (-1.15,15.85)	**Quercetin**		
4.06 (-4.10,12.22)	-0.36 (-8.55,7.83)	-0.98 (-6.87,4.91)	1.02 (-7.24,9.27)	**-6.33 (-12.52,-0.14)**	**Resveratrol**	
0.57 (-6.46,7.60)	-3.86 (-10.92,3.21)	**-4.48 (-8.67,-0.29)**	-2.48 (-9.62,4.67)	**-9.83 (-14.43,-5.23)**	-3.50 (-7.63,0.64)	**Control**

Figure S37 Sensitivity analysis of atretic follicle excluding small-sample studies (n < 5 animals per group). (A) Forest plot comparing the effects of TCM monomers with the control group based on a random-effects model. (B) League table summarizing all pairwise comparisons. Data are expressed as SMDs with 95% CIs.
